# Photo-induced oxidant-free oxidative C–H/N–H cross-coupling between arenes and azoles

**DOI:** 10.1038/ncomms14226

**Published:** 2017-02-01

**Authors:** Linbin Niu, Hong Yi, Shengchun Wang, Tianyi Liu, Jiamei Liu, Aiwen Lei

**Affiliations:** 1 College of Chemistry and Molecular Sciences, The Institute for Advanced Studies (IAS), Wuhan University, Hubei 430072, China; 2 State Key Laboratory and Institute of Elemento-Organic Chemistry, Nankai University, Tianjin 300071, China

## Abstract

Direct cross-coupling between simple arenes and heterocyclic amines under mild conditions is undoubtedly important for C–N bonds construction. Selective C(sp^2^)-H amination is more valuable. Herein we show a selective C(sp^2^)-H amination of arenes (alkyl-substituted benzenes, biphenyl and anisole derivatives) accompanied by hydrogen evolution by using heterocyclic azoles as nitrogen sources. The reaction is selective for C(sp^2^)-H bonds, providing a mild route to *N*-arylazoles. The KIE (kinetic isotope effect) experiment reveals the cleavage of C–H bond is not involved in the rate-determining step. Kinetic studies indicate the first-order behaviour with respect to the arene component. It is interesting that this system works without the need for any sacrificial oxidant and is highly selective for C(sp^2^)-H activation, whereas C(sp^3^)-H bonds are unaffected. This study may have significant implications for the functionalization of methylarenes which are sensitive to oxidative conditions.

## Abstract (web summary)

Functionalization of unactivated C–H bonds is an attractive strategy to introduce functionality without the need for pre-functionalized starting materials. Here the authors combine cobalt catalysis with photoredox catalysis, allowing arene-azole cross-coupling under oxidant-free conditions.

N-arylazoles, a particularly prevalent class of nitrogen-containing compounds, have long been privileged structures in agricultural chemical and biopharmaceuticals (Fig. 1a)^1^,^2^. Therefore, preparation of these valuable compounds in a sustainable way is always at the frontier of organic synthesis. The transition metal-catalysed (Pd, Cu and so on) cross-coupling of aryl halides (Ar–X) with azoles have served as a powerful tool for constructing C–N bonds^3^,^4^,^5^,^6^,^7^,^8^. Directly using simple arenes (Ar–H) to replace Ar–X is undoubtedly the most step- and atom-economical method, representing an ideal route for C–N bonds construction. To avoid the separate prefunctionalization and defunctionalization steps^9^,^10^,^11^, the new strategy that directly utilizes C–H/X–H (N, S, O and so on) of two nucleophiles is considered to be an ideal mode for bond formation. In recent years, the analogous technology for the oxidative C–H/N–H cross-coupling to form C–N bonds would be an important synthetic advance^12^,^13^,^14^. However, current strategies required high temperatures or stoichiometric quantities of oxidant, which caused the high consumption of material and energy^15^,^16^,^17^. Hence, more environmental and sustainable perspectives for the alternative catalytic system are therefore highly desirable for C–N construction.

Methylarenes, a group of the most important raw chemical materials, have been widely utilized as acyl, alkyl, carboxylate and/or alcohol sources^18^, providing the selective formation of C–C and C-heteroatom bonds. Due to the fact that activation of methylarenes is always under oxidative conditions, the process is always coupled with the transfer of electrons and protons. For example, *p*-xylene can react with the oxidant to generate the radical cation primarily, which is prossibly transferred into benzyl radical^19^. The benzylic C(sp^3^)-H bonds functionalization has been proceeded via the potentially benzyl radical intermediate in numerous reports^20^,^21^,^22^. In 2013, Zhu and co-workers^23^ achieved the C(sp^3^)-H amination of methylarenes with azoles using *n*-Bu_4_NI as the catalyst and *tert*-butyl hydroperoxide as the oxidant (Fig. 1b). However, limited examples have been developed for the arylation of azoles using simple methylarenes.

Recently, photocatalysis mediated by visible light has been widely developed, serving as a mild and powerful tool for bond formation^24^,^25^,^26^,^27^,^28^,^29^,^30^. As pioneering work for arene C–H functionalization, the visible-light-mediated C(sp^2^)-H amination using azoles as nitrogen sources and O_2_ as terminal oxidant has been achieved recently^31^. Due to the fact that the methyl group of methylarene is sensitive to oxidative conditions, especially to dioxygen in photocatalytic system^32^, developing a new strategy for the selective C(sp^2^)-H functionalization of methylarenes is urgently required. It is always proposed that two-electron and two-proton were lost in traditional oxidative coupling. It demands extra stoichiometric oxidants and proton acceptors as the sacrificial reagents in the cross-coupling reactions, leading to a number of wasteful by-products or oxidative side reactions^9^,^10^,^11^. The oxidant-free condition combining photocatalyst with cobalt complex provides a new strategy for bond formation between two nucleophiles^33^,^34^,^35^. Inputting energy by photocatalysis and employing the cobalt catalyst to take two electrons, we show an external oxidant-free oxidative C(sp^2^)-H amination of arenes, especially the methylarenes, whereas the C(sp^3^)-H bonds of the methylarenes are unaffected (Fig. 1c). Selective C–N formation is achieved accompanying with H_2_ evolution in the same system. A variety of *N*-arylazoles can be successfully afforded under this oxidant-free condition. The arene radical cation species serves the key role for the selective C(sp^2^)-H amination.

## Results

### Investigation of reaction conditions

Initially, we optimized the conditions using the *p*-xylene (**1a**) and pyrazole (**2a**) as model substrates. On the basis of our previous studies in the field of oxidant-free system^34^, we firstly chose Ru(bpy)_3_(PF_6_)_2_ as photocatalyst and Co(dmgH)_2_Cl_2_ as the proton-reduction catalyst. Unfortunately, no desired product was achieved (Table 1, entry 1). Then, different commonly used photocatalysts were screened (Table 1, entries 2–4). To our delight, we successfully achieved our C–N coupling product **3a** in 23% yield using acridinium moiety as the photocatalyst (Supplementary Table 1, entry 2), which has a strong oxidative ability^36^. After investigating the reaction conditions (Supplementary Table 1), we obtained a satisfactory yield of amination product with four equivalent *p*-xylene **1a** and one equivalent pyrazole **2a** as a suitable proportion of substrates (Table 1, entry 4). Almost the same yield of H_2_ was detected by gas chromatography-thermal conductivity detector (see Supplementary Methods for details). Additionally, the control experiments indicated that the cobalt catalyst, photocatalyst and visible light was indispensable for this transformation (Table 1, entries 5–7).

To highlight the advantage of our oxidant-free system, several oxidants were added into the system to replace the cobalt catalyst (Table 2). In general, the photocatalytic systems in the presence of oxidant show low reactivity for this C–N coupling reaction and afforded low yields of amination product **3a**. *tert*-butyl hydroperoxide, K_2_S_2_O_8_, benzoquinone, PhI(OAc)_2_ and TEMPO (2,2,6,6-tetramethylpiperidin-1-yloxyl) were not suitable for this amination process, showing very poor efficiency (Table 2, entries 1–5). Using O_2_ as oxidant, it did not make a satisfactory result, and a relatively small amount of aldehyde **9** was generated concomitantly, while the result was slightly improved (Table 2, entry 6). These results revealed that the oxidant-free system shows great superiority over photocatalyst/oxidant system for this selective C(sp^2^)-H amination.

### Substrate scope

**Figure 2:**
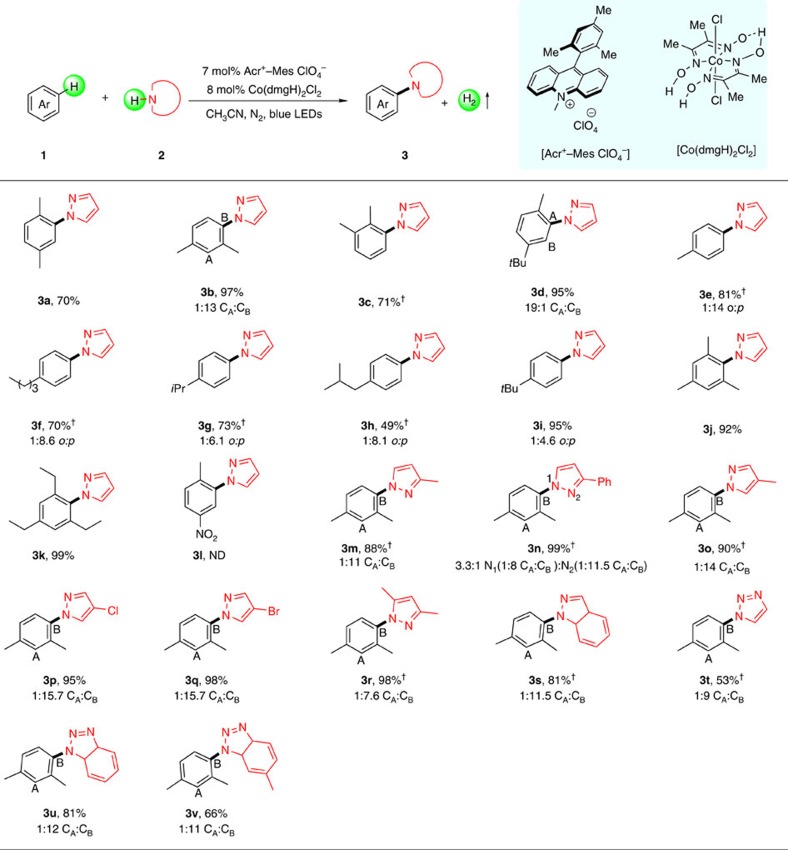
Substrate scope of arenes 1 with azoles 2. Reaction conditions: **1** (1.2 mmol), **2** (0.3 mmol), Acr^+^-Mes ClO_4_^−^ (7 mol%) and Co(dmgH)_2_Cl_2_ (8 mol%) in CH_3_CN (5.0 ml) under N_2_ atmosphere, irradiated by 3 W blue LEDs at 25 °C for 24 h. The ratio of the isomer was determined by NMR. ^†^Arenes **1** (2.0 ml) and CH_3_CN (3.0 ml) were used. Isolated yields are shown. ND, not detected.

Having confirmed the high efficiency and unique advantages of this amination protocol without any sacrificial oxidant, we firstly examined the scope of methylarenes **1** with pyrazole **2a**. To our delight, the current catalytic system was suitable for a wide range of methylarenes (Fig. 2). Other dialkyl-substituted benzenes like *m*-xylene, *o*-xylene and 1-(*tert*-butyl)-4-methylbenzene could be successfully converted into the corresponding amination products in good yields (**3b**–**3d**) and with high site-selectivity, especially for *o*-xylene. It was found that toluene was still effective in our catalytic system and can afford the C–N product in 81% yield (**3e**). Subsequently, a series of monoalkyl-substituted benzenes were examined under standard conditions, in which the functional groups such as methyl, *n*-Bu, *i*-Pr, *i*-Bu and *t*-Bu were surprisingly compatible with satisfactory efficiency (**3f**–**3i**). It is worth noting that trialkyl-substituted benzenes could also be suitable for this system, achieving the C(sp^2^)-H amination product in high efficiency (**3j** and **3k**). In spite of trialkyl-substituted benzenes (mesitylene and 1,3,5-triethylbenzene) containing weak benzylic C–H bonds, which may be prone for benzylic C–H amination, notably only aromatic ring C(sp^2^)-H amination were resolutely observed, showing excellent chemoselectivity of this oxidant-free system. Furthermore, amination process can be conducted with methylarenes loadings as low as one equivalent relative to nitrogen nucleophiles, albeit with a slight decrease in chemical yield to 50% (Supplementary Fig. 55). However, there was no reaction for *p*-nitrotoluene due to the inhibition of nitro group, possibly indicating a radical process (**3l**). At the same time, a series of heterocyclic amines and its tolerance of functional groups were studied to expand the synthetic utility of this methodology. Delightfully, the pyrazoles with different substituent could be converted into the desired products (**3m**–**3r**) in excellent yields. Several functional groups on the pyrazole moiety (Cl and Br) were well tolerated (**3p** and **3q**), which provides an opportunity for further transformation. It is very gratifying to see that 1*H*-indazole could also be transformed into the desired product in 81% yield (**3s**). Moreover, amination products were obtained in high efficiency for 1,2,3-1*H*-triazole and 1*H*-benzotriazole (**3t**–**3v**). Unfortunately, non-cyclic amines such as aniline, diphenylamine, dibenzylamine and *N*-acetylacetamide could not be used as suitable nitrogen sources under the same catalytic system.

**Figure 3:**
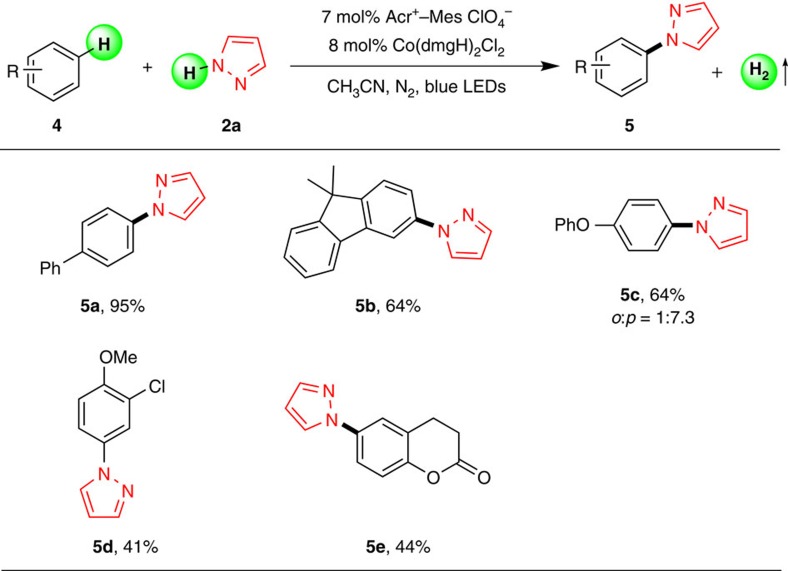
Substrate scope of pyrazole 2a with other aromatic arenes 4. Reaction conditions: **4** (1.2 mmol), **2a** (0.3 mmol), Acr^+^-Mes ClO_4_^−^ (7 mol%) and Co(dmgH)_2_Cl_2_ (8 mol%) in CH_3_CN (5.0 ml) under N_2_ atmosphere, irradiated by 3 W blue LEDs at 25 °C for 24 h. The ratio of the isomer was determined by nuclear magnetic resonance. Isolated yields are shown.

To further expand the potential usage of our oxidant-free amination methodology, a variety of other aromatic rings were also investigated and the results are summarized in Fig. 3. The good yields and high selectivity for biphenyl and 9,9-dimethyl-9*H*-fluorene were effortlessly obtained (**5a** and **5b**). Importantly, the electron-rich arenes such as diphenyl oxide and anisole, which have been regarded as commonly used arenes in C(sp^2^)-H amination, were also suitable for this system and achieved the desired products in moderate yields (**5c** and **5d**). In particular, dihydrocoumarin was smoothly tolerated in this amination protocol (**5e**), which has exhibited interesting biological activities^37^.

## Discussion

**Figure 4:**
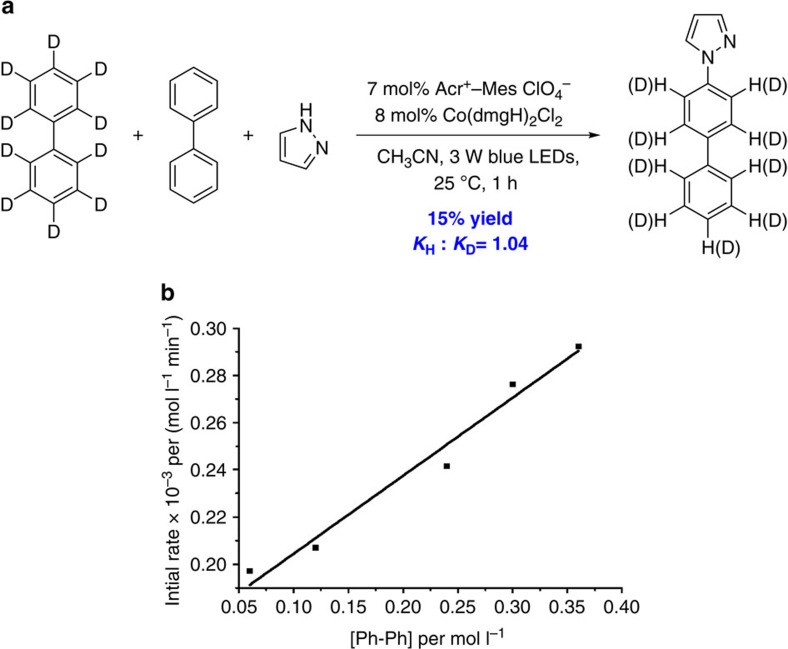
Investigation for mechanistic insights. (**a**) The intermolecular kinetic of isotopic effect experiment. (**b**) Kinetic plots of the reactions with different concentrations of biphenyl **4a.**

An intermolecular kinetic isotope effect experiment was carried out to further study this oxidant-free C–H/N–H cross-coupling reaction. As shown in Fig. 4a, the KIE (kinetic isotope effect) of 1.04 was observed from an intermolecular competition (see Supplementary Fig. 56 for details). It revealed that the C–H bond cleavage of arene might not be involved in the rate-determining step. Next, the further detailed kinetic behaviour of biphenyl was also tested (see Supplementary Methods). As depicted in Fig. 4b, the initial reaction rate was affected by changing the concentration of the biphenyl, demonstrating first-order dependencies on biphenyl.

**Figure 5:**
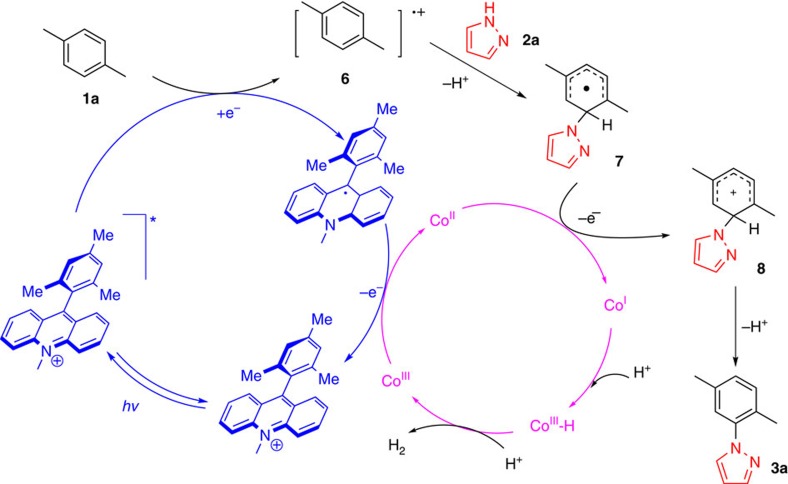
Proposed mechanism for oxidant-free C–H amination. The plausible mechanism involves oxidation of arene to generate arene radical cation, nucleophilic attack of azoles and visible–light photoredox cobalt-catalysed formation of amination product and H_2_.

Based on the above kinetic data and previous reports, a plausible reaction mechanism was illustrated in Fig. 5. The photocatalyst and cobalt synergistic catalysis is hypothesized to be responsible for the electron transfer and proton release. Initially, photocatalyst acridinium is excited to *Mes-Acr-Me^+^ (*E*_1/2 red_=+2.06 V) by blue light-emitting diodes^38^, which is capable of oxidizing methylarenes **1** or biphenyl **4a** (*E*_1/2 red_=+1.96 V) to corresponding radical cation **6**. On the other hand, Co (III) catalyst could oxidize the Mes-Acr-Me radical (*E*_1/2 red_=−0.49 V)^36^ to finish the cycle of the photocatalyst and generate the Co (II) species at the same time. It is considered that the generated aromatic ring radical cation is attacked by nucleophile pyrazole **2a** (*E*_1/2 red_=+2.27 V)^31^, which is difficult to be oxidized by the excited state acridinium, to intermediate **7**. Then, the radical adduct **7** is likely to undergo a single electron transfer with Co (II) catalyst to **8**, which quickly loses one proton and generates the amination product. At the same time, Co (I) could capture the present proton in the reaction system, yielding Co (III)-H. As a result, H_2_ can be released and the Co (III) is regenerated, as well through the protonation of Co (III)-H^39^,^40^.

In conclusion, we have developed an oxidant-free and selective C(sp^2^)-H bond amination of arenes utilizing a photocatalyst and cobalt oxime complex. A series of alkyl-substituted benzenes, biphenyl and anisole derivatives under current catalytic system could be well tolerated, providing a new way to *N*-arylazoles synthesis. The KIE experiment and kinetic study provide important mechanistic insights. Our further efforts in this study using another nucleophile for the C(sp^2^)-H functionalization are currently underway.

## Methods

### General procedure (**3a**)

A solution of *p*-xylene **1a** (1.2 mmol), 1*H*-pyrazole **2a** (0.3 mmol), photocatalyst (7 mol%) and Co^III^(dmgH)_2_Cl_2_ (8 mol%) in degassed CH_3_CN (5.0 ml) were stirred under nitrogen atmosphere and irradiated by 3 W blue light-emitting diodes at 25 °C for 24 h. After completion of the reaction, H_2_ was detected by gas chromatography-thermal conductivity detector. The aqueous solution was extracted with ethyl acetate (3 × 10 ml) and the combined extracts were dried with anhydrous Na_2_SO_4_. The solvent was removed under reduced pressure by rotary evaporation, and then the pure product was obtained by flash column chromatography on silica gel (eluent: petroleum ether/ethyl acetate=25:1), directly to give the desired product **3a** in 70% yield as a colourless liquid. For ^1^H NMR and ^13^C NMR spectra of compounds **3a**–**3k**, **3m**–**3v** and **5a**–**5e** see Supplementary Fig. 1–54. Full experimental details can be found in the Supplementary Methods.

### Data availability

The authors declare that the data supporting the findings of this study are available within the article and its Supplementary Information Files.

## Additional information

**How to cite this article:** Niu, L. *et al*. Photo-induced oxidant-free oxidative C–H/N–H cross-coupling between arenes and azoles. *Nat. Commun.* **8,** 14226 doi: 10.1038/ncomms14226 (2017).

**Publisher's note:** Springer Nature remains neutral with regard to jurisdictional claims in published maps and institutional affiliations.

## Supplementary Material

Supplementary Information Supplementary figures, supplementary table, supplementary methods and supplementary references.

## Figures and Tables

**Figure 1:**
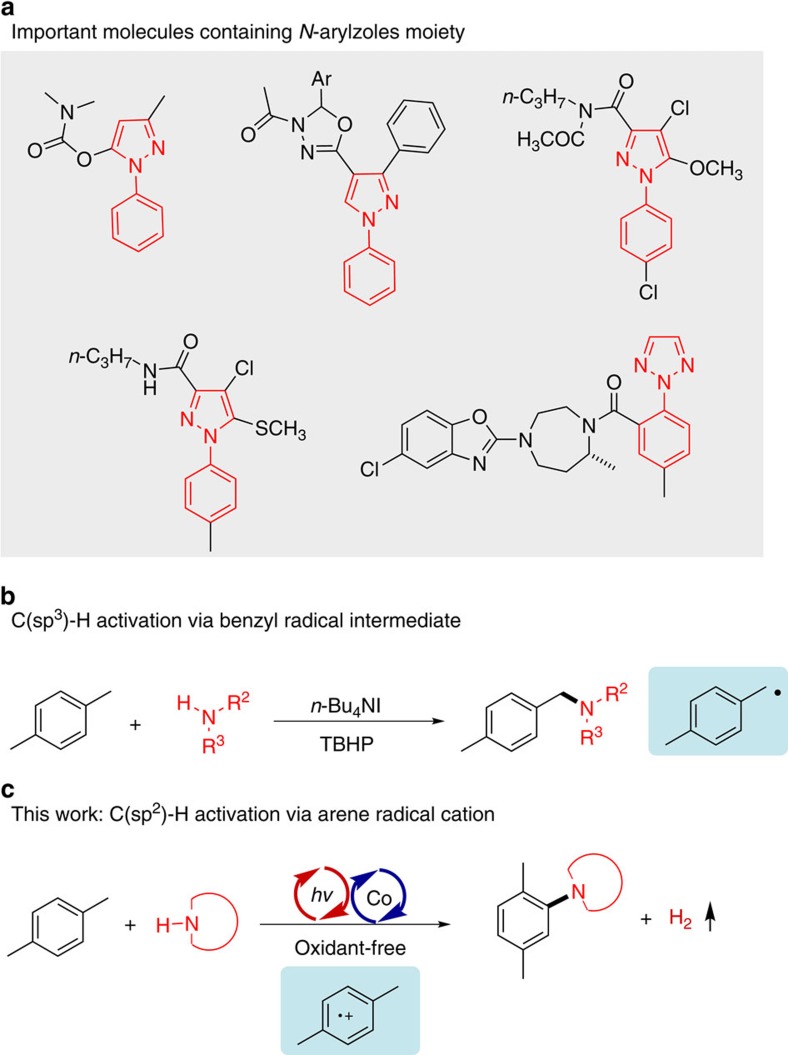
Reaction design. (**a**) Important molecules containing *N*-arylzoles moiety. (**b**) Amination of benzylic C-H bond by Zhu and co-workers^23^. (**c**) The C(sp^2^)-H amination of methylarenes under oxidant-free conditions.

**Table 1:**
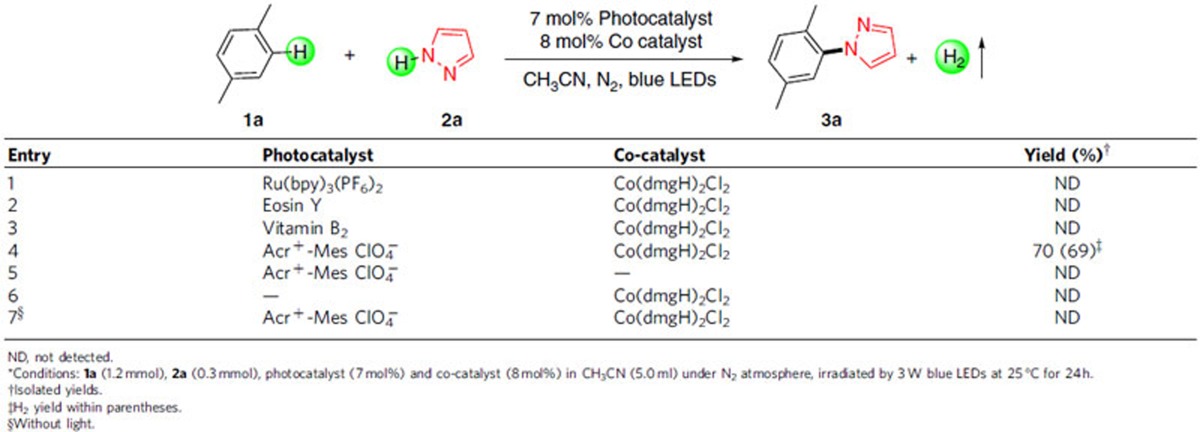
Optimization of the reaction conditions^*^.

**Table 2:**
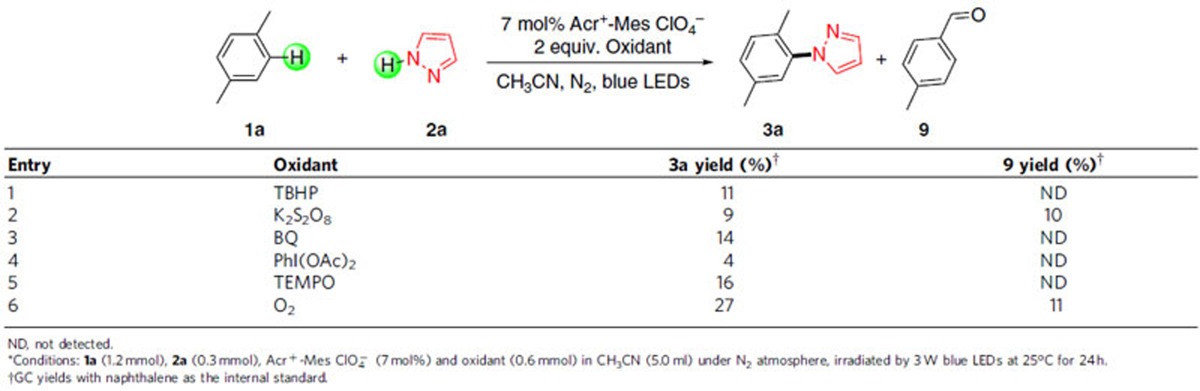
Comparison between photo/cobalt system and photo/oxidant system.^*^
