# Supplementary material for: Photo-induced oxidant-free oxidative C–H/N–H cross-coupling between arenes and azoles
Source: Nat Commun. 2017 Feb 1;8:14226. doi: 10.1038/ncomms14226 (PMC5296647; doi:10.1038/ncomms14226)
Supplement: Supplementary Information — Supplementary figures, supplementary table, supplementary methods and supplementary references. [file ncomms14226-s1.pdf]

Supplementary Figures

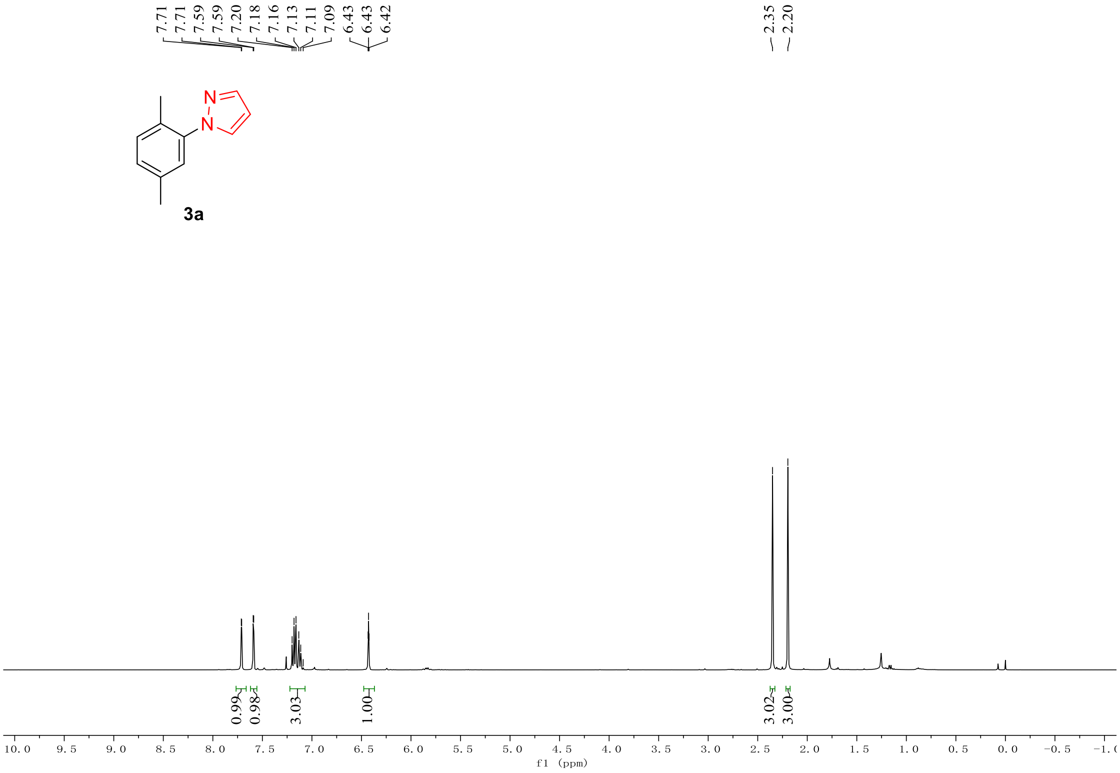

Supplementary Figure 1. ^{1}H NMR (400 MHz, $CDCl_3$) spectrum of 3a

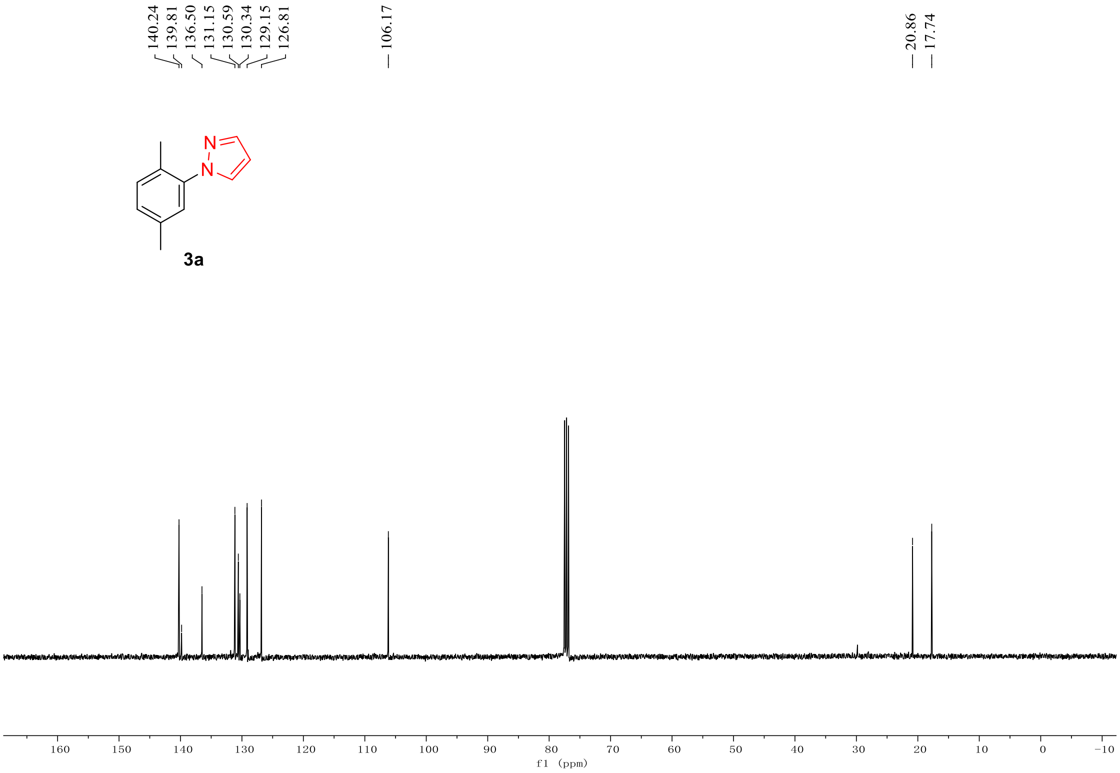

Supplementary Figure 2. ^{13}C NMR (101MHz, $CDCl_3$) spectrum of 3a

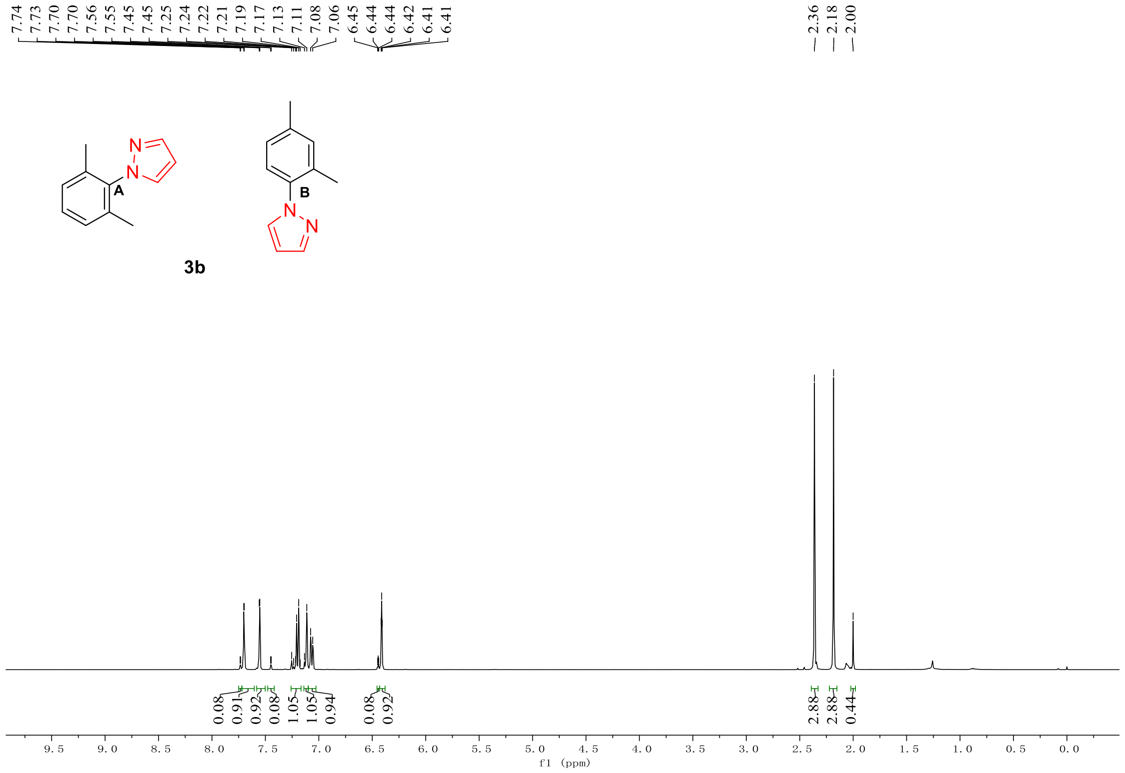

Supplementary Figure 3. ^{1}H NMR (400 MHz, $CDCl_3$) spectrum of 3b

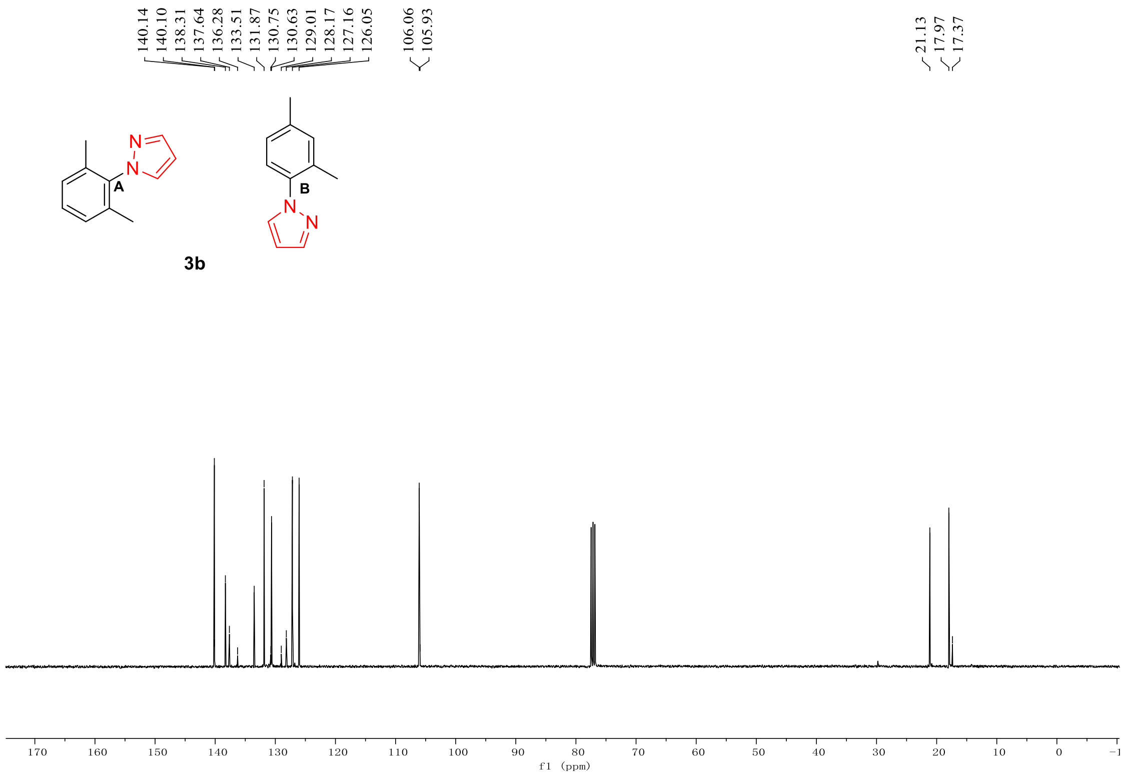

Supplementary Figure 4. ^{13}C NMR (101MHz, $CDCl_3$) spectrum of 3b

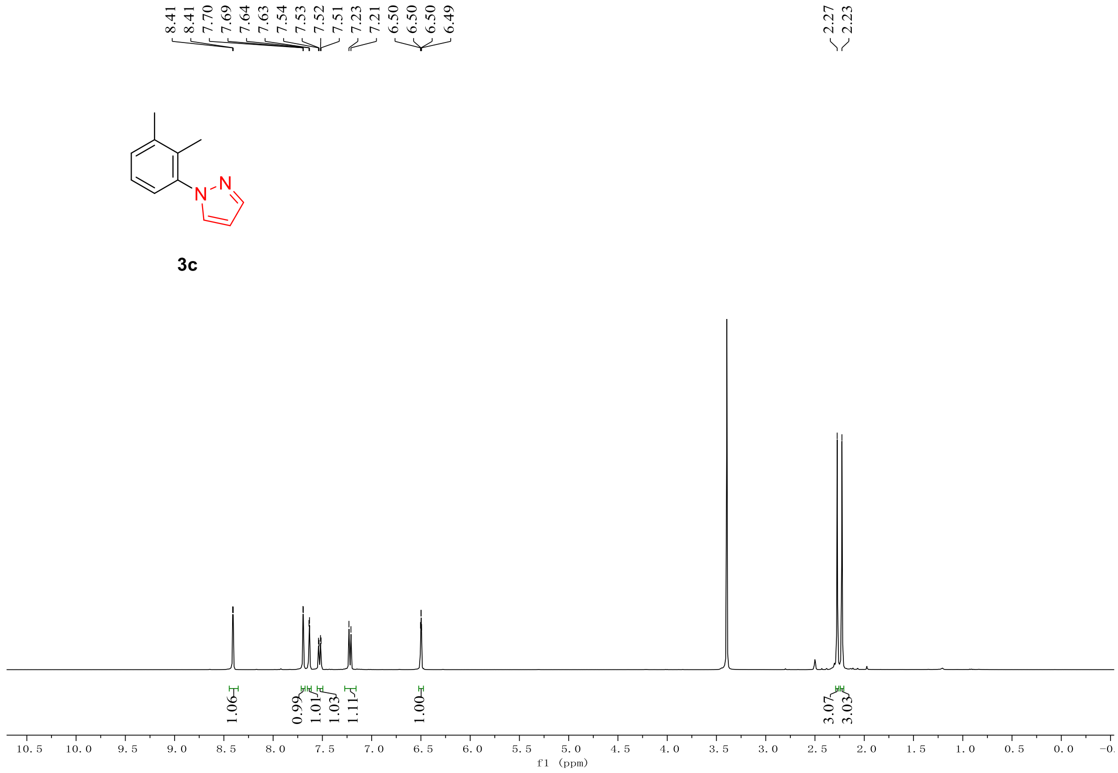

Supplementary Figure 5. ^{1}H NMR (400 MHz, DMSO) spectrum of 3c

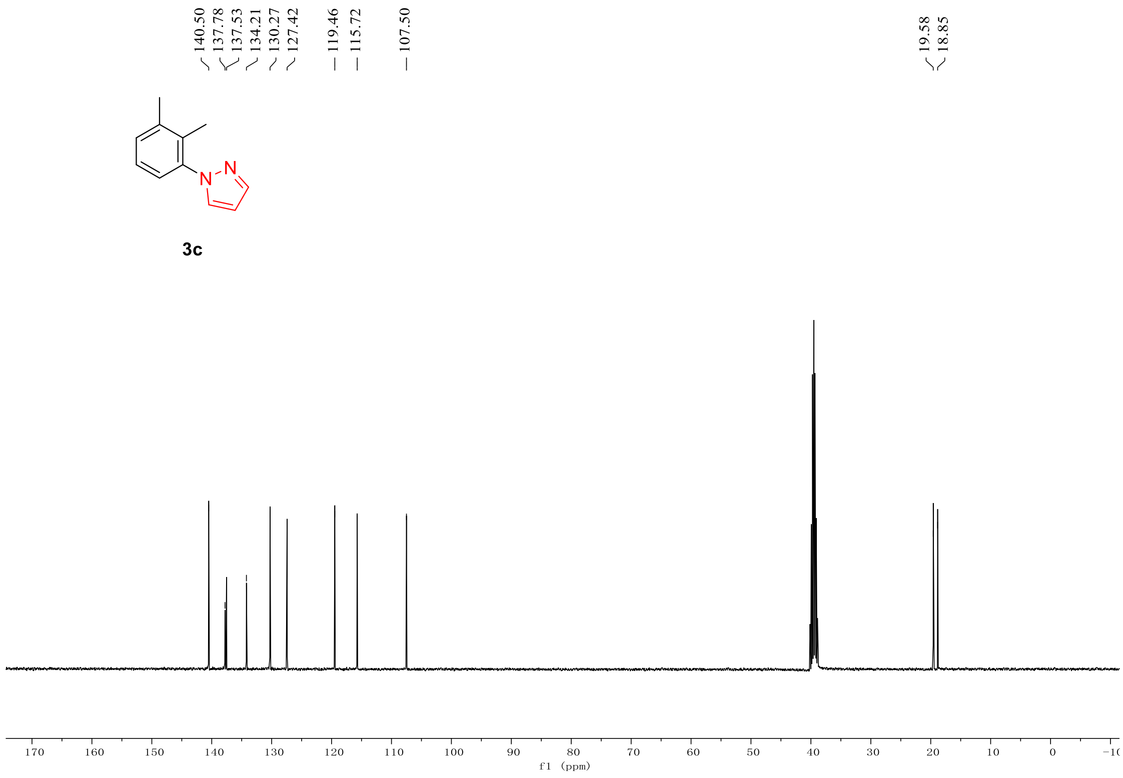

Supplementary Figure 6. ^{13}C NMR (101MHz, DMSO) spectrum of 3c

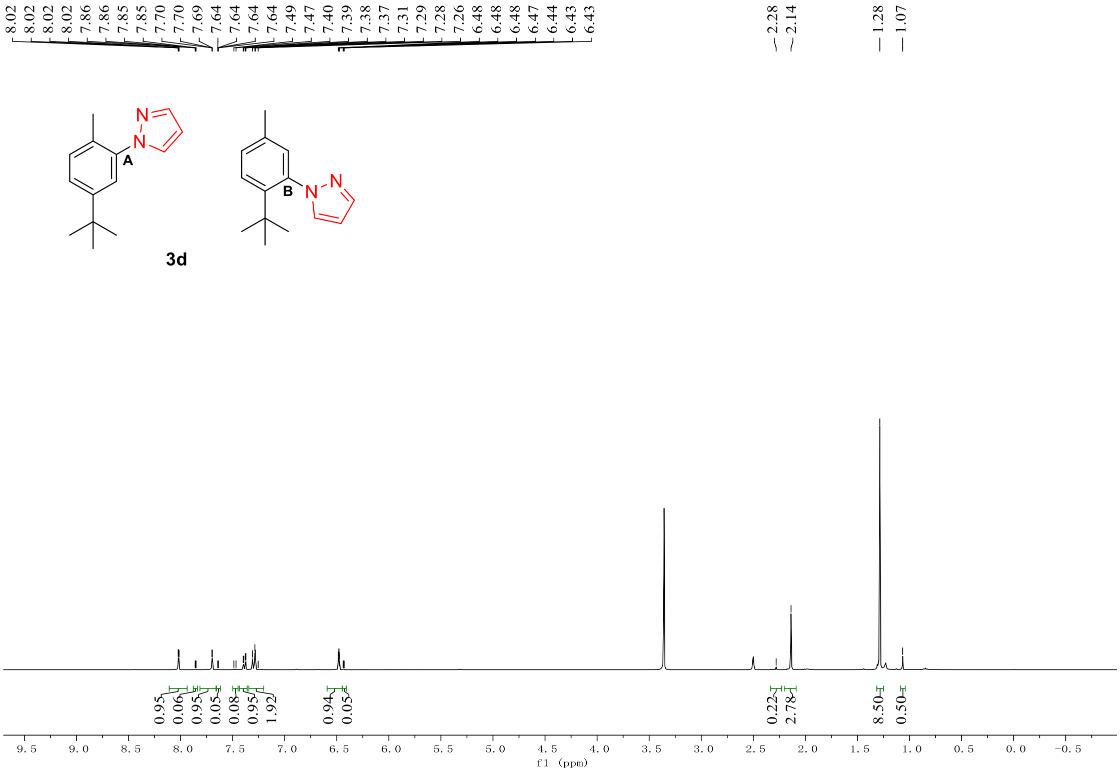

Supplementary Figure 7. **^{1}H NMR (400 MHz, DMSO) spectrum of 3d**

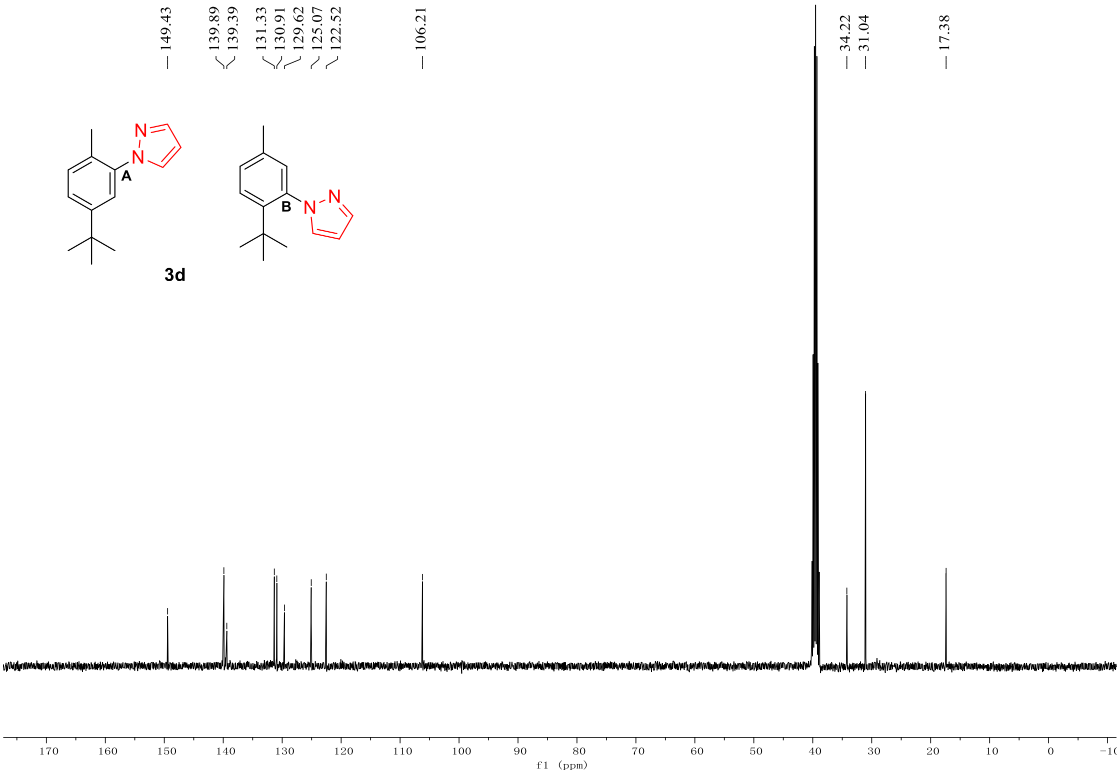

Supplementary Figure 8. **^{13}C NMR (101MHz, DMSO) spectrum of 3d**

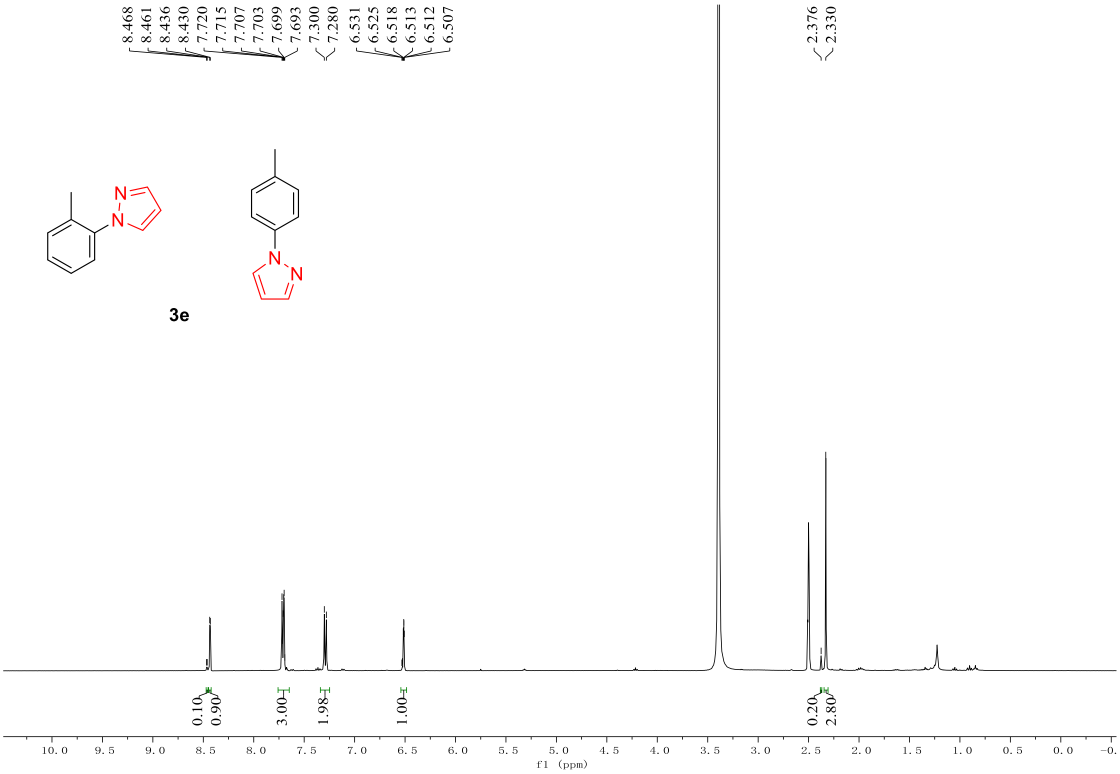

Supplementary Figure 9. ^{1}H NMR (400 MHz, DMSO) spectrum of 3e

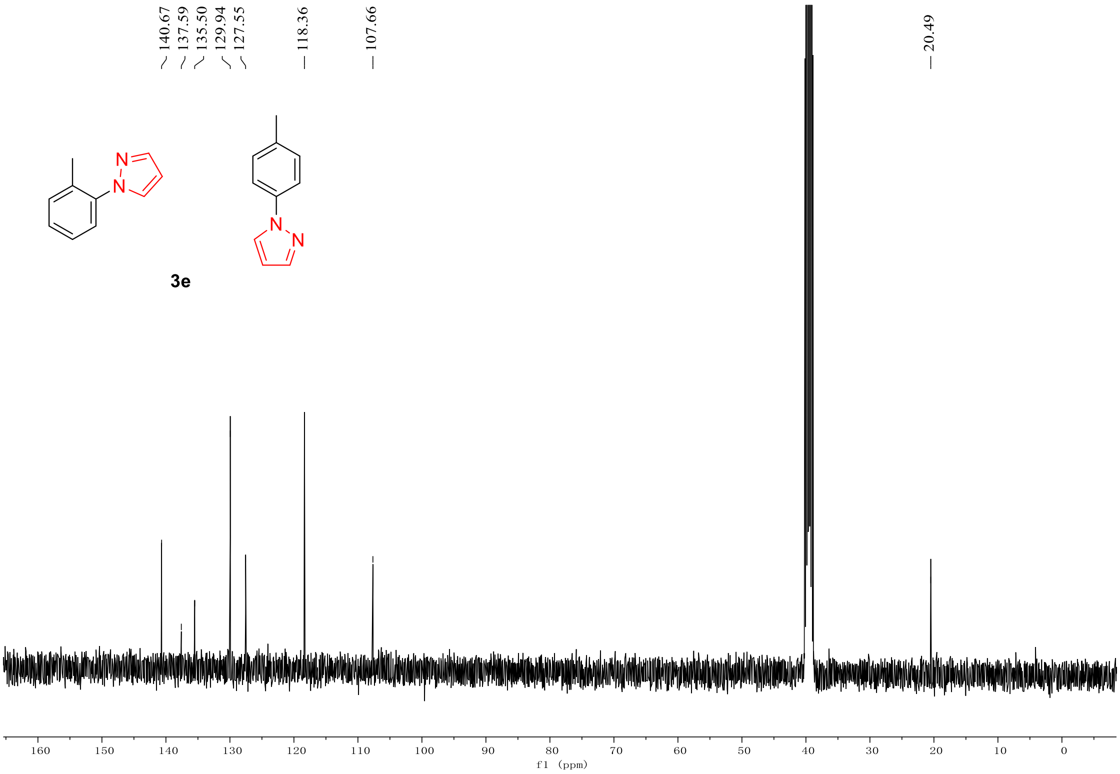

Supplementary Figure 10. ^{13}C NMR (101MHz, DMSO) spectrum of 3e

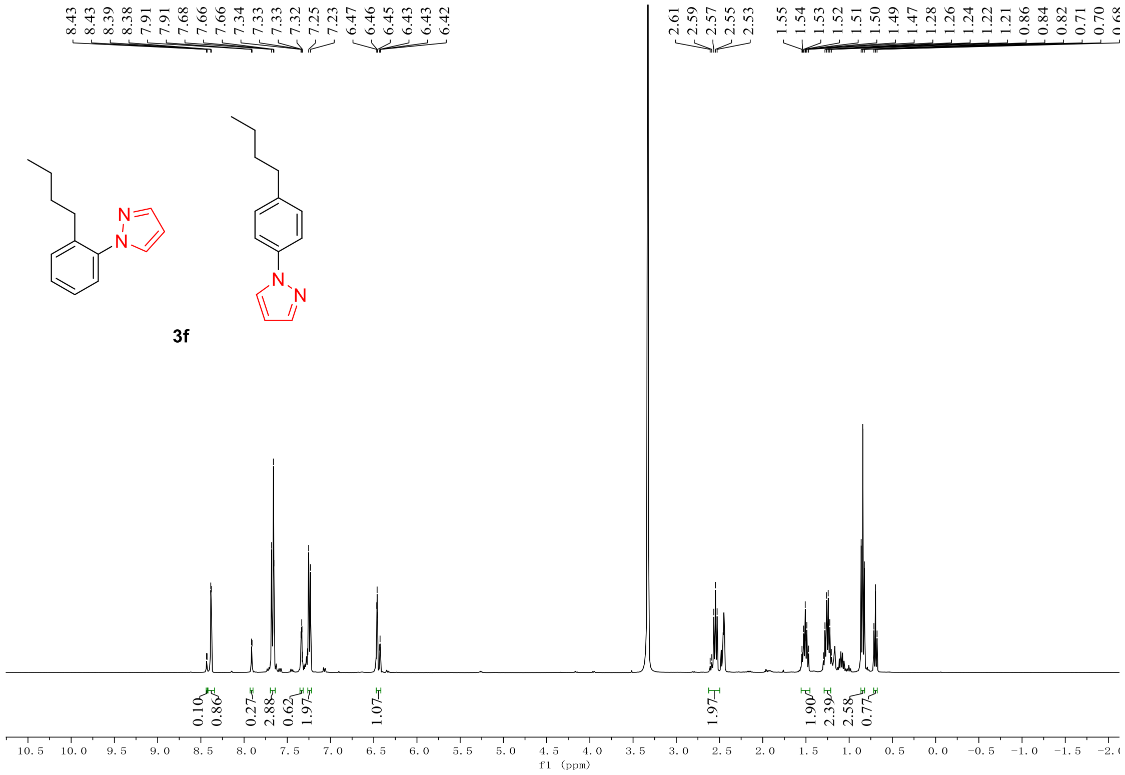

Supplementary Figure 11. ^{1}**H NMR (400 MHz, DMSO) spectrum of 3f**

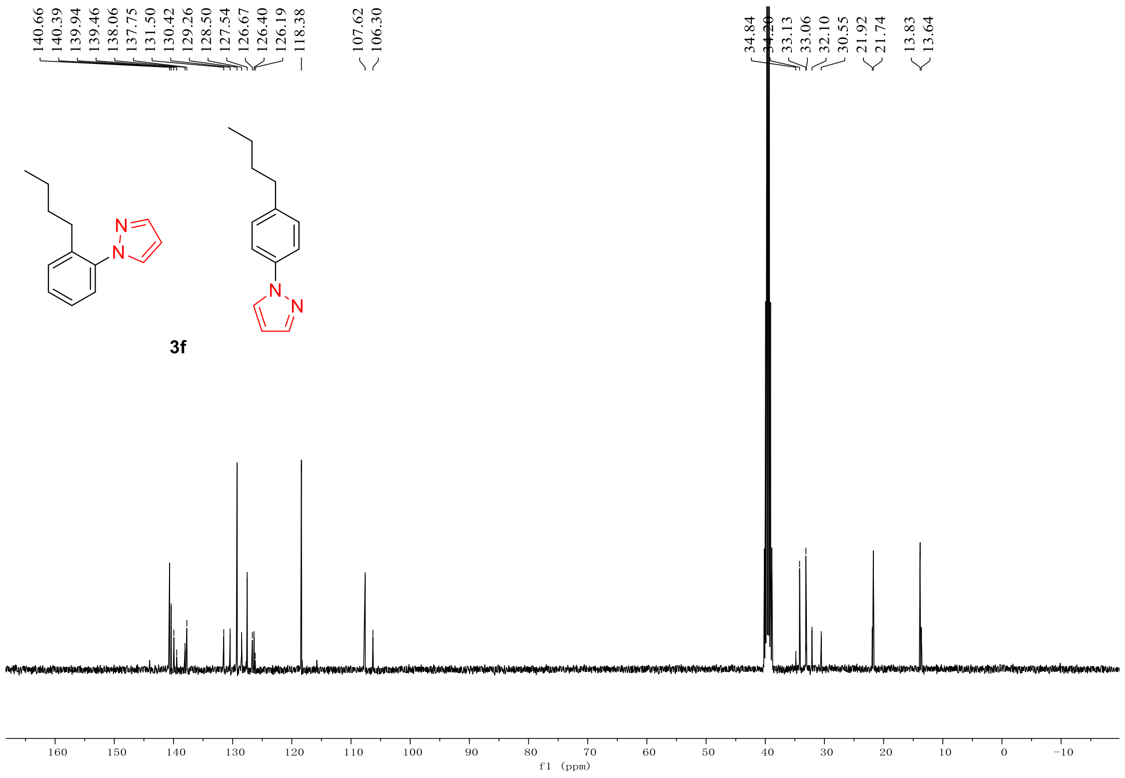

Supplementary Figure 12. ^{13}**C NMR (101MHz, DMSO) spectrum of 3f**

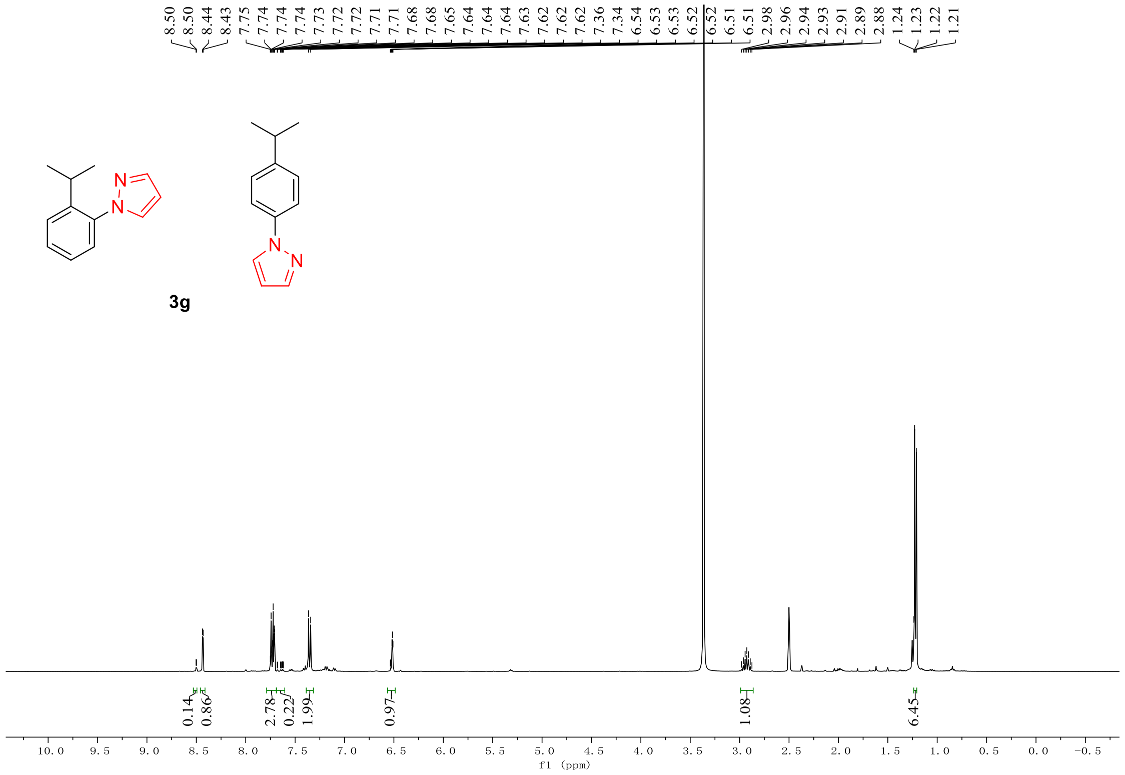

Supplementary Figure 13. ^{1}H NMR (400 MHz, DMSO) spectrum of 3g

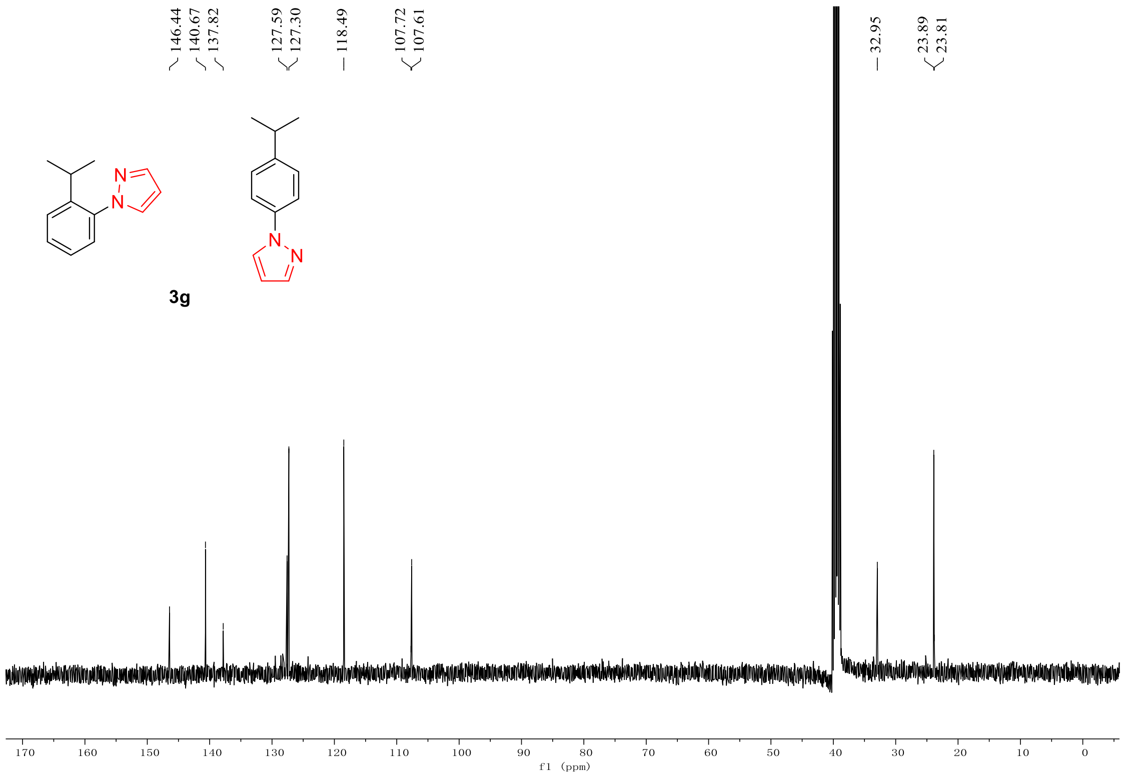

Supplementary Figure 14. ^{13}C NMR (101MHz, DMSO) spectrum of 3g

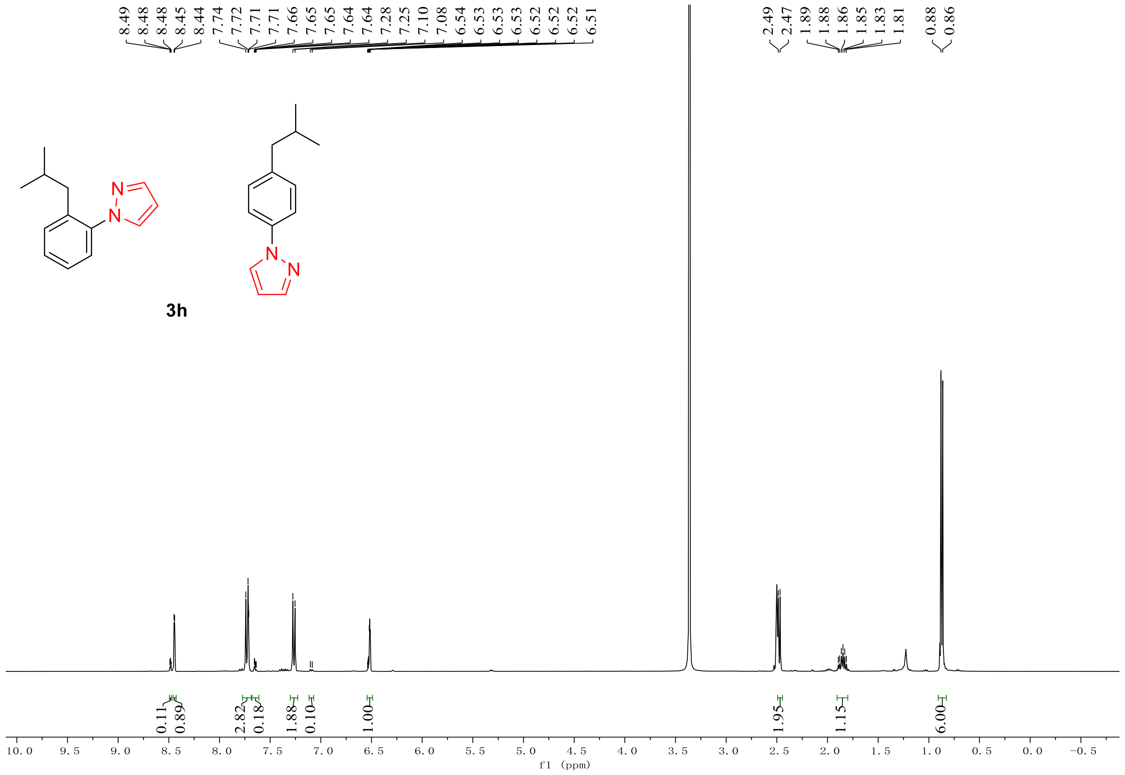

Supplementary Figure 15. ^{1}H NMR (400 MHz, DMSO) spectrum of 3h

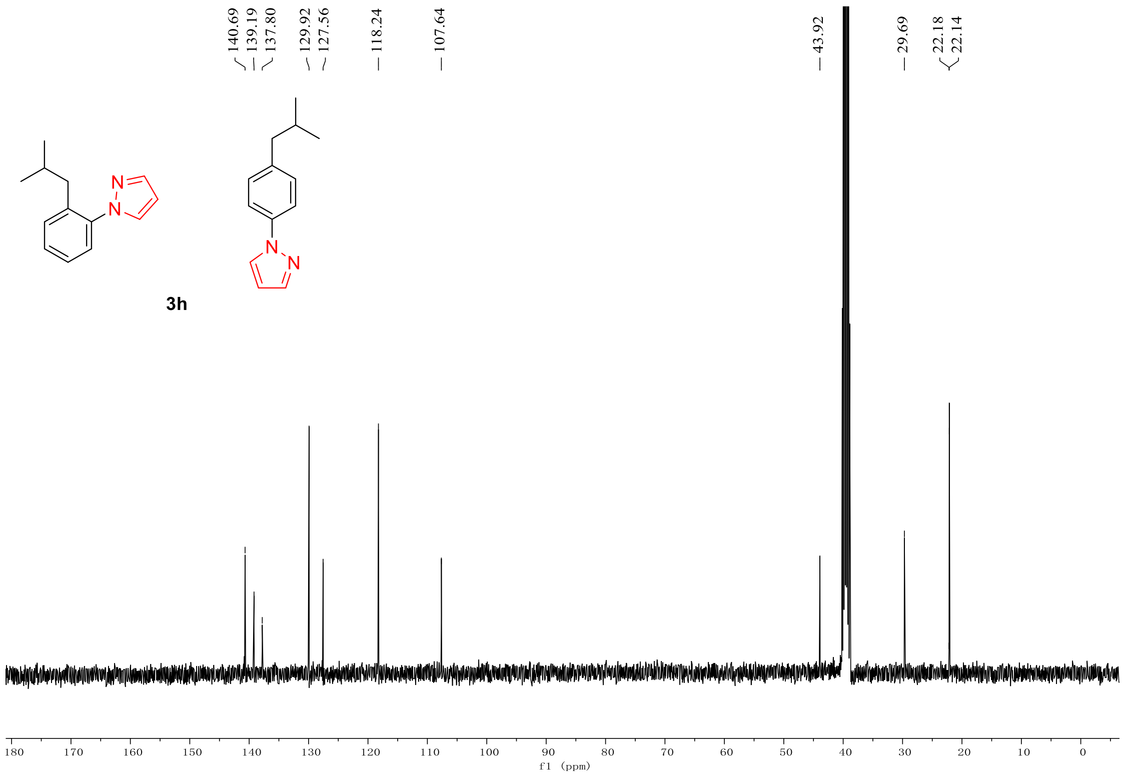

Supplementary Figure 16. ^{13}C NMR (101MHz, DMSO) spectrum of 3h

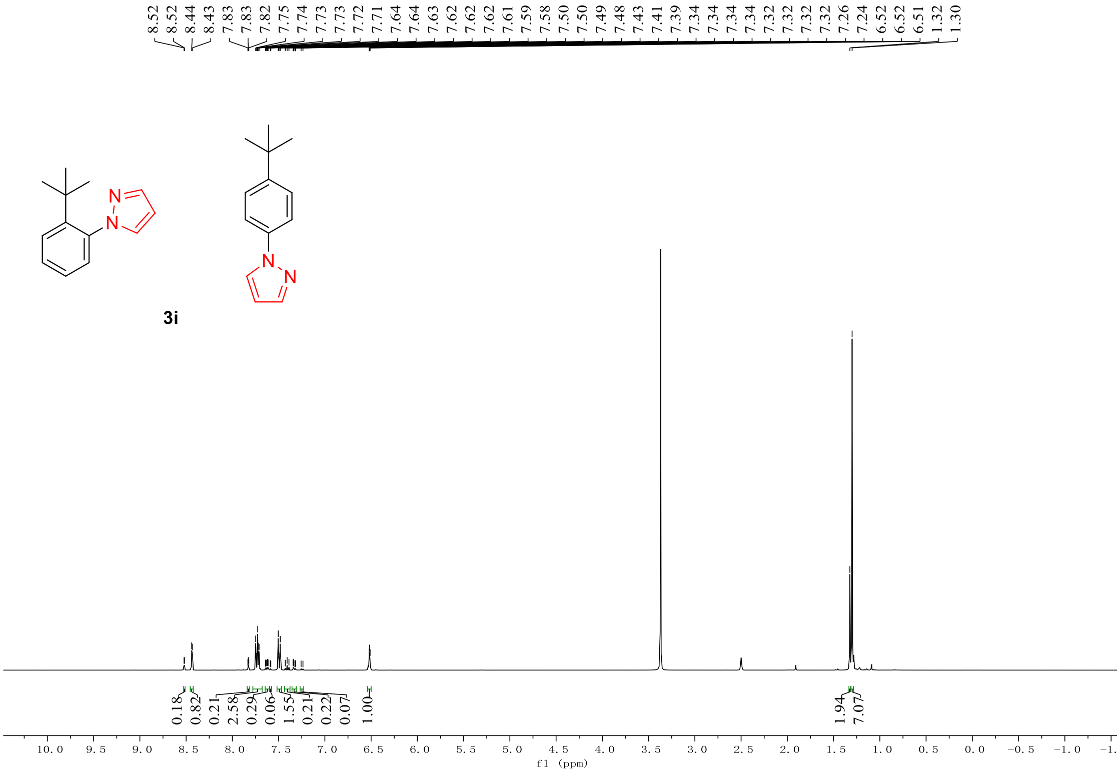

Supplementary Figure 17. ^{1}H **NMR (400 MHz, DMSO) spectrum of 3i**

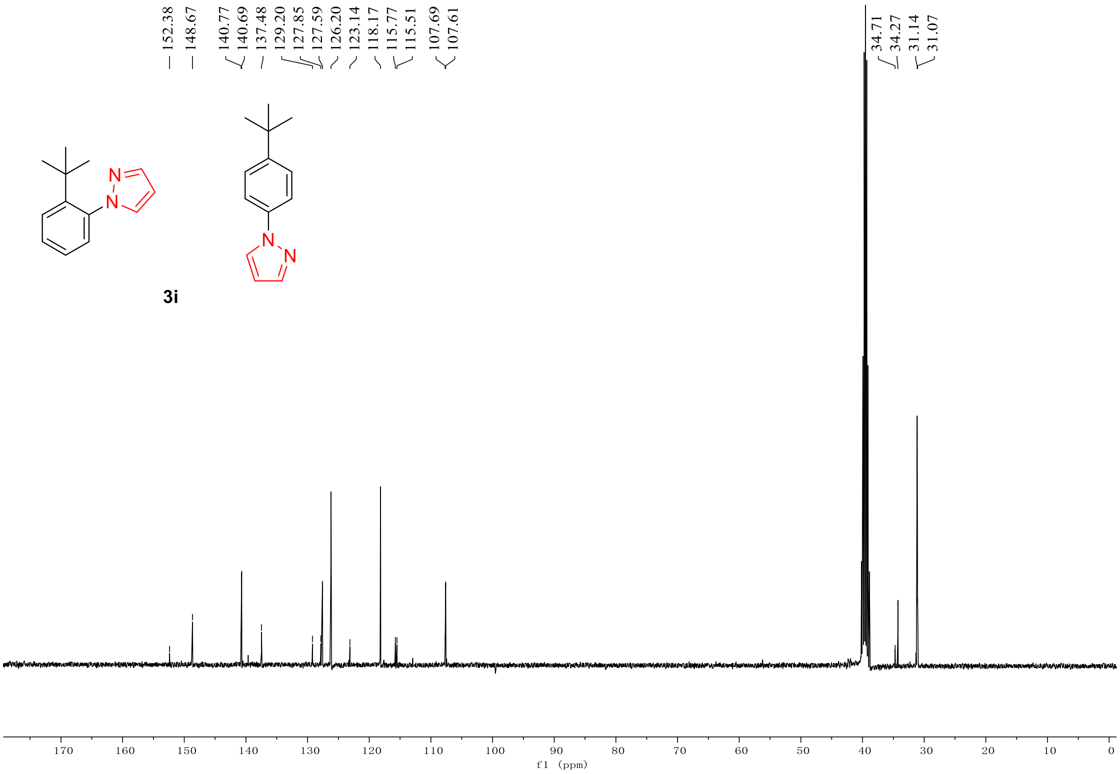

Supplementary Figure 18. ^{13}C **NMR (101MHz, DMSO) spectrum of 3i**

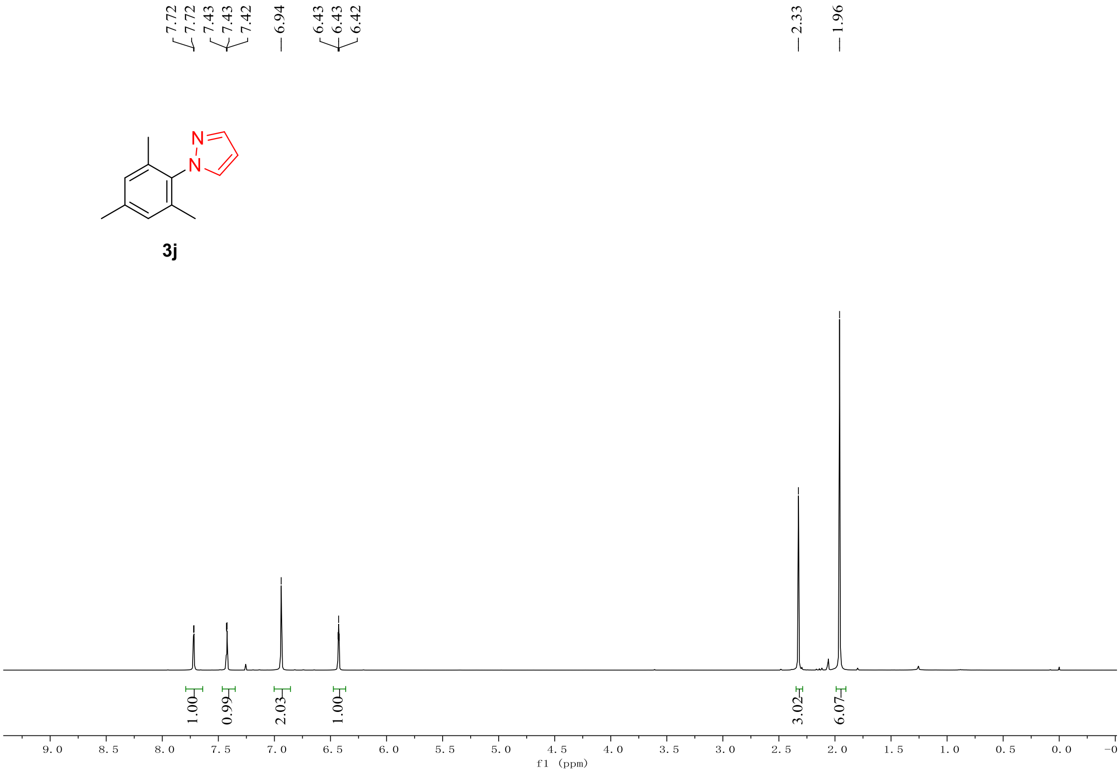

Supplementary Figure 19. ^{1}H NMR (400 MHz, $CDCl_3$) spectrum of 3j

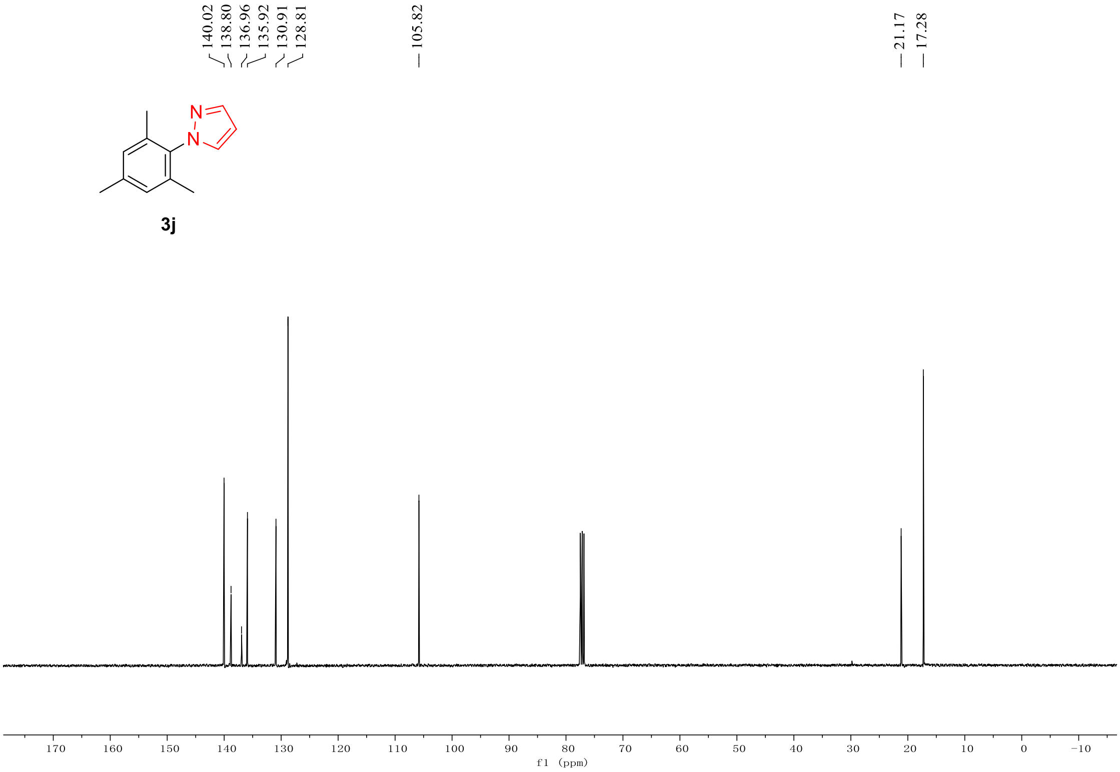

Supplementary Figure 20. ^{13}C NMR (101MHz, $CDCl_3$) spectrum of 3j

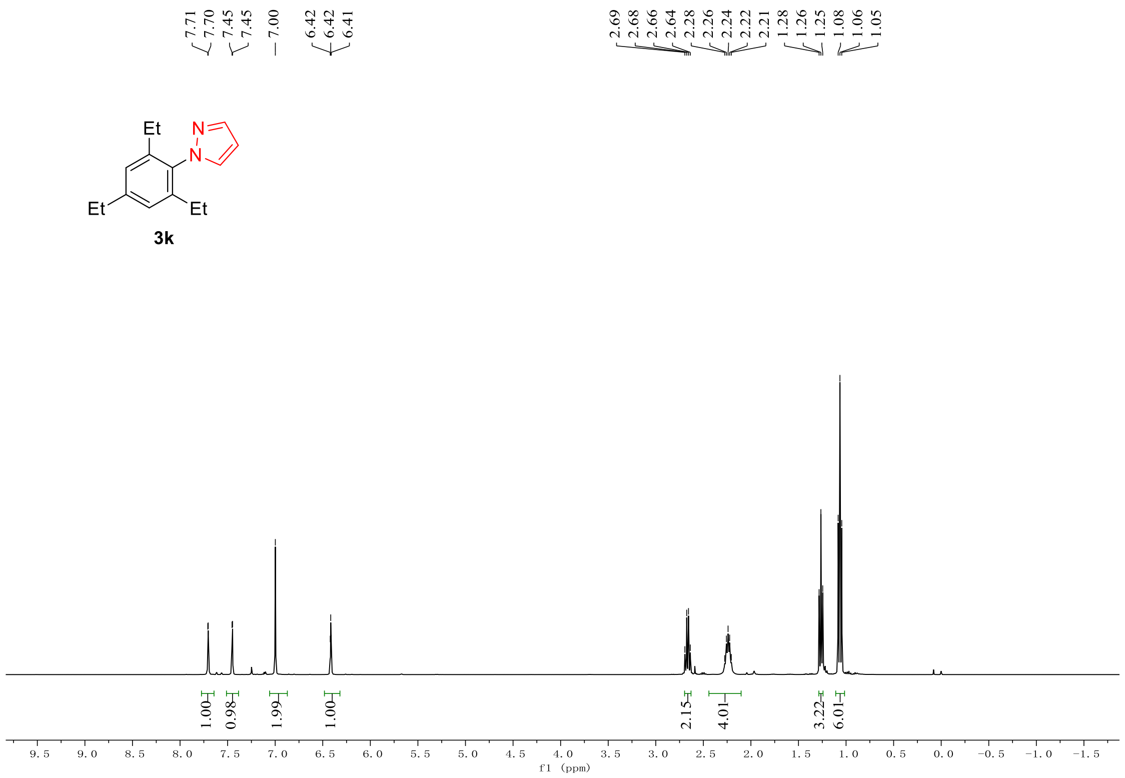

Supplementary Figure 21. **^{1}H NMR (400 MHz, $CDCl_3$) spectrum of 3k**

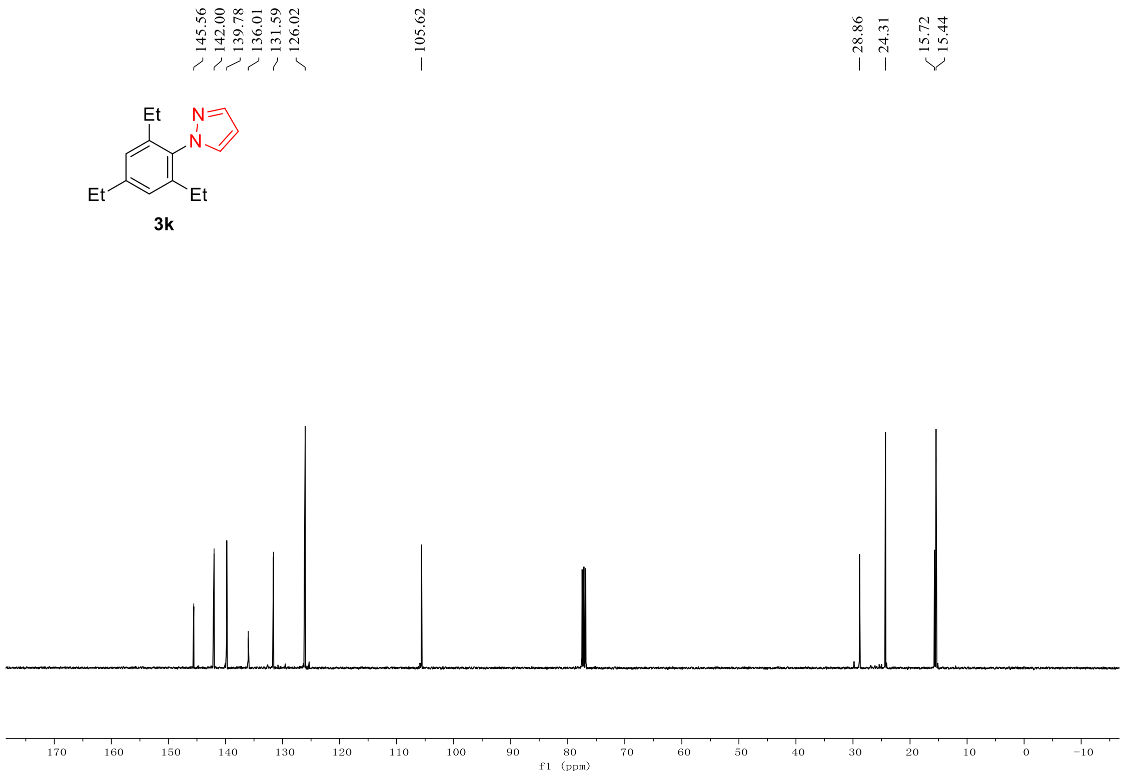

Supplementary Figure 22. **^{13}C NMR (101MHz, $CDCl_3$) spectrum of 3k**

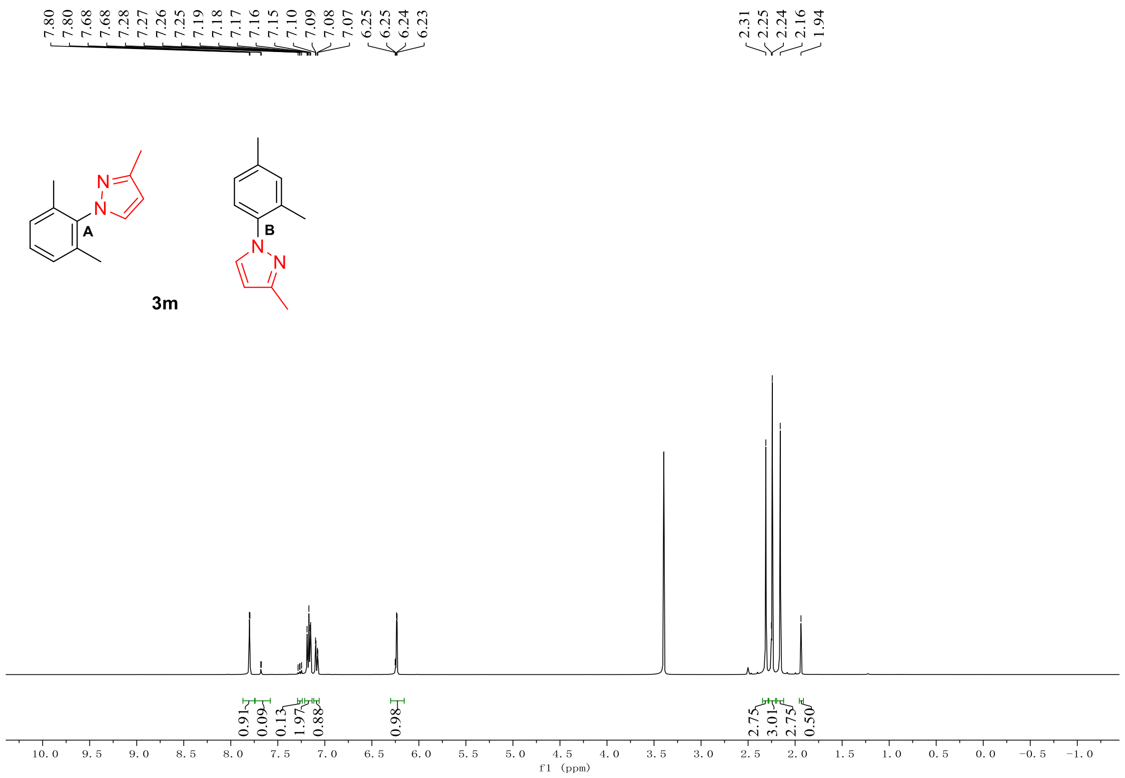

Supplementary Figure 23. ^{1}H NMR (400 MHz, DMSO) spectrum of 3m

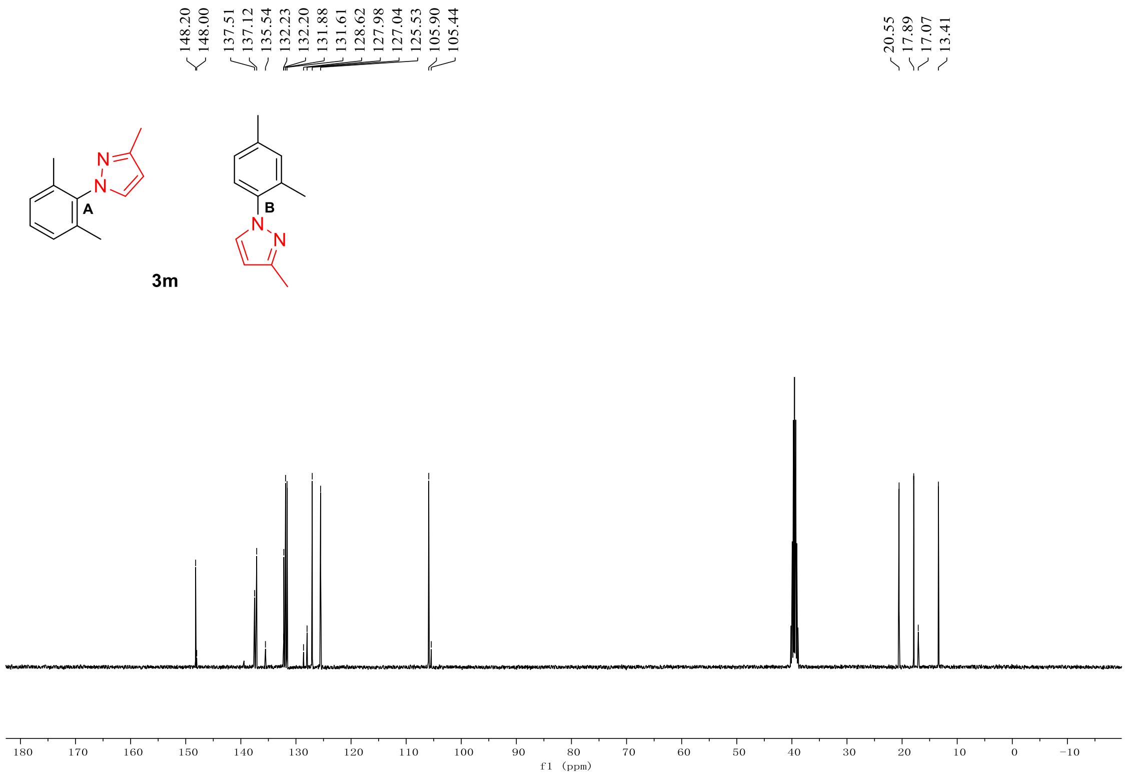

Supplementary Figure 24. ^{13}C NMR (101MHz, DMSO) spectrum of 3m

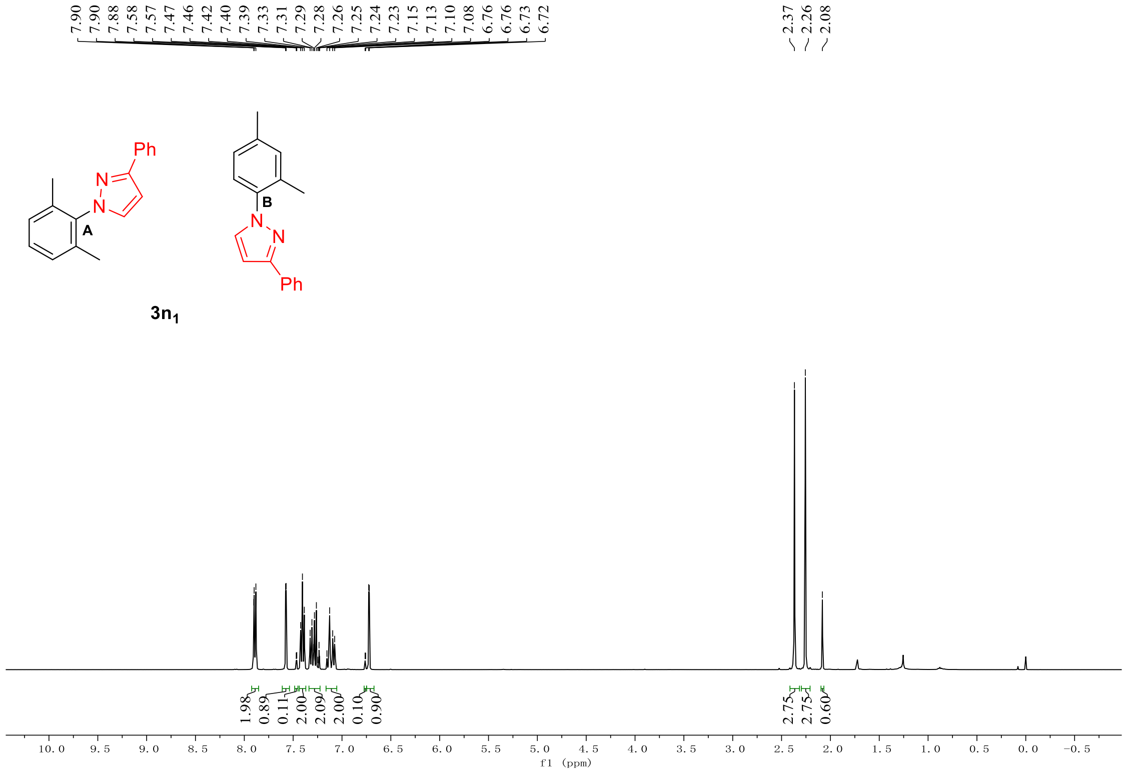

Supplementary Figure 25. ^{1}**H NMR (400 MHz, CDCl$_3$) spectrum of 3n$_1$**

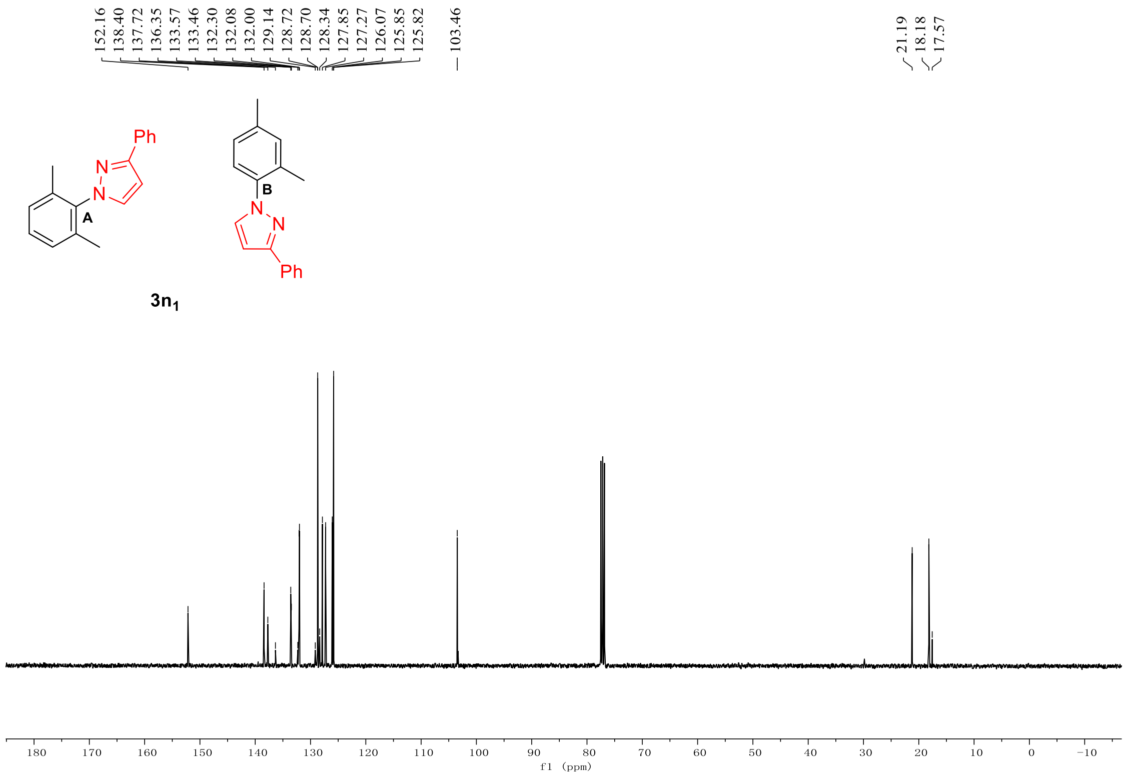

Supplementary Figure 26. ^{13}**C NMR (101MHz, CDCl$_3$) spectrum of 3n$_1$**

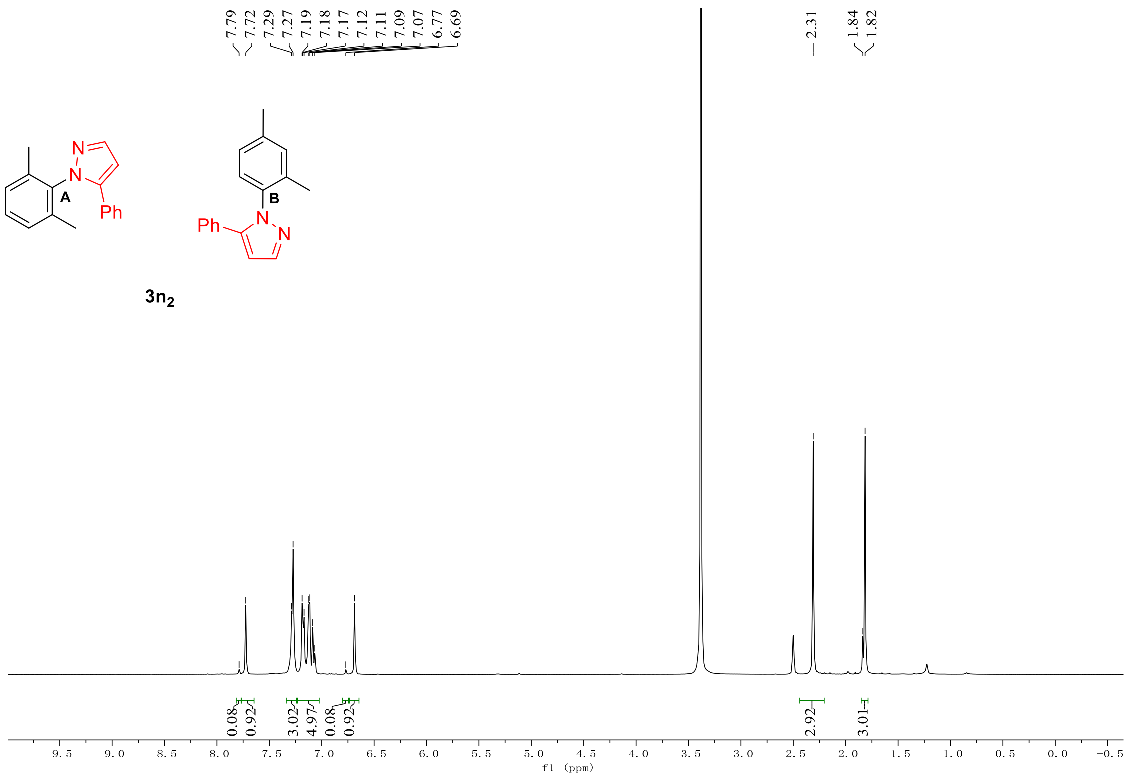

Supplementary Figure 27. ^{1}H NMR (400 MHz, DMSO) spectrum of 3n$_2$

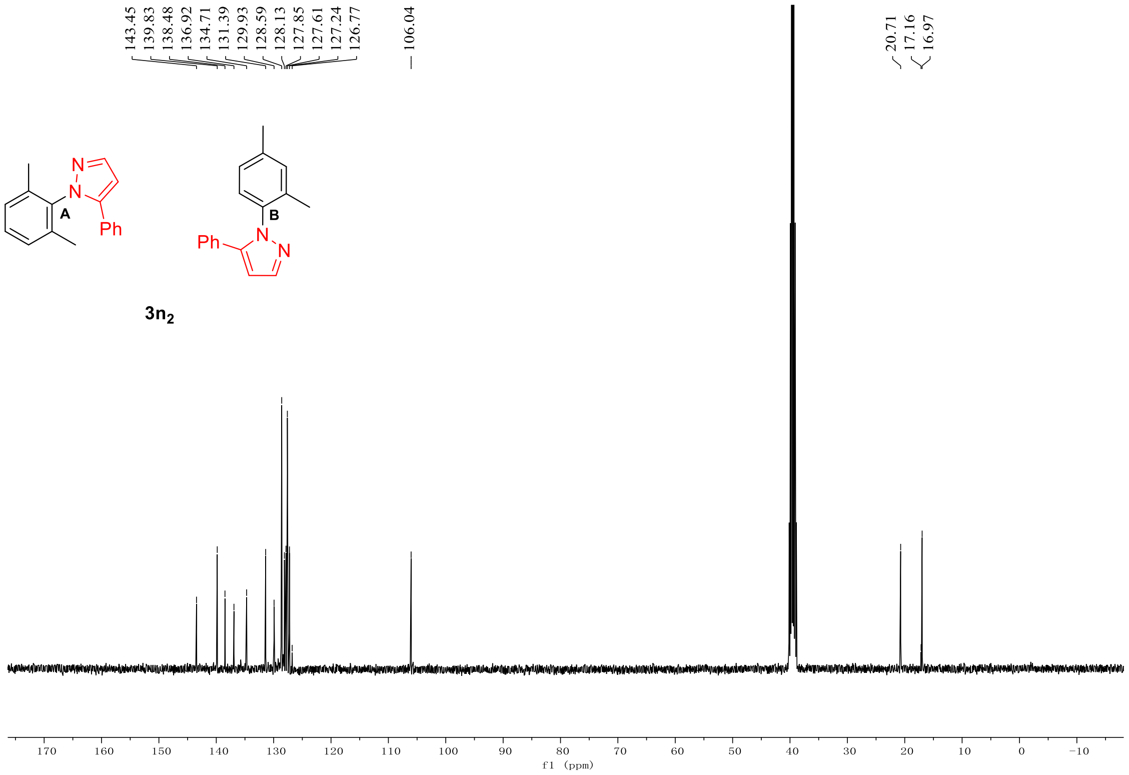

Supplementary Figure 28. ^{13}C NMR (101MHz, DMSO) spectrum of 3n$_2$

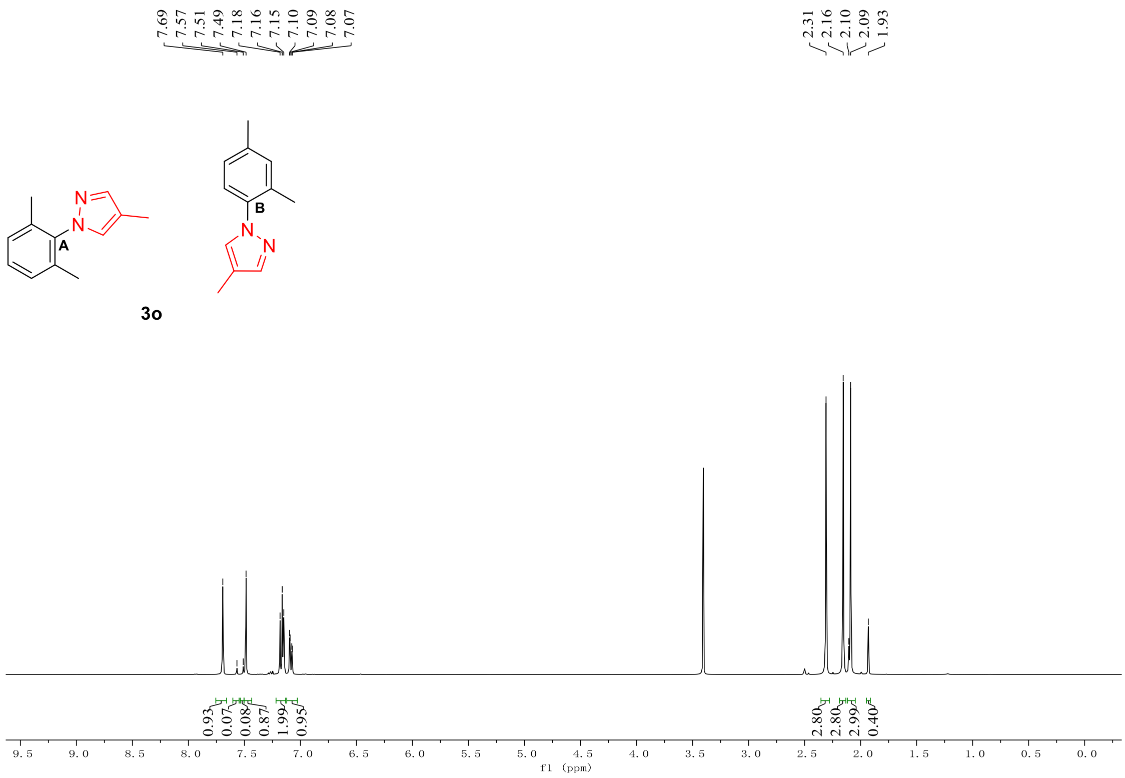

Supplementary Figure 29. ^{1}H NMR (400 MHz, DMSO) spectrum of 3o

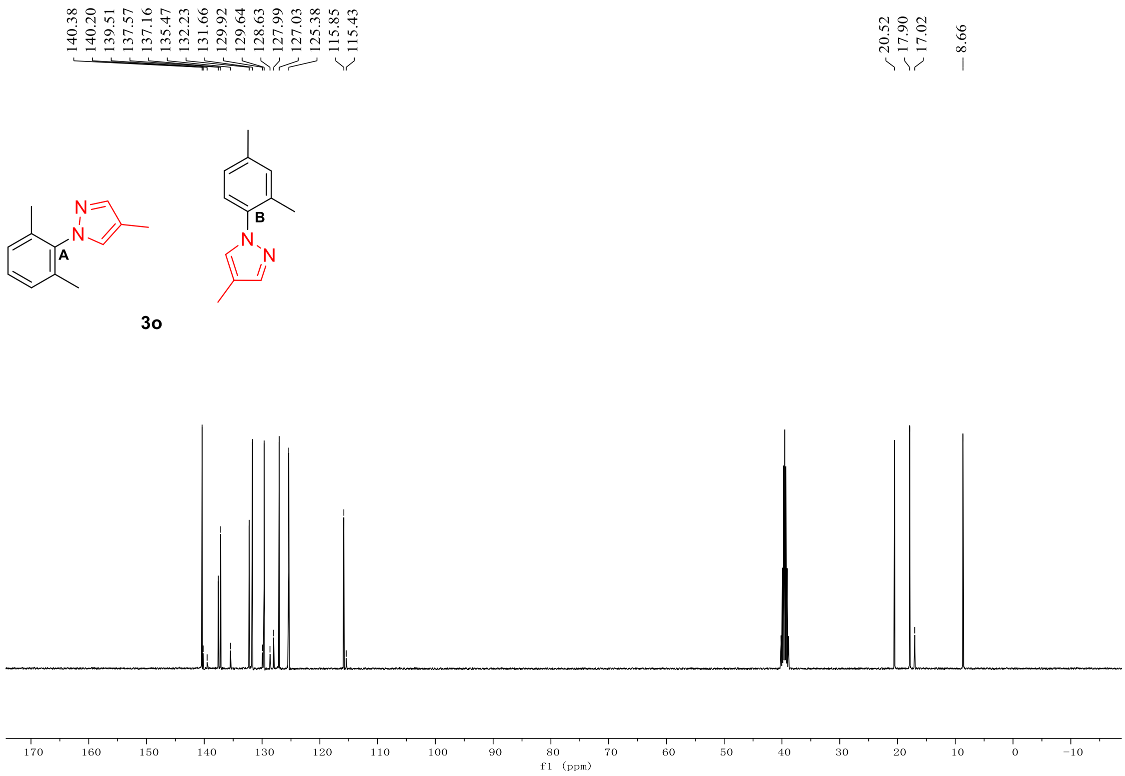

Supplementary Figure 30. ^{13}C NMR (101MHz, DMSO) spectrum of 3o

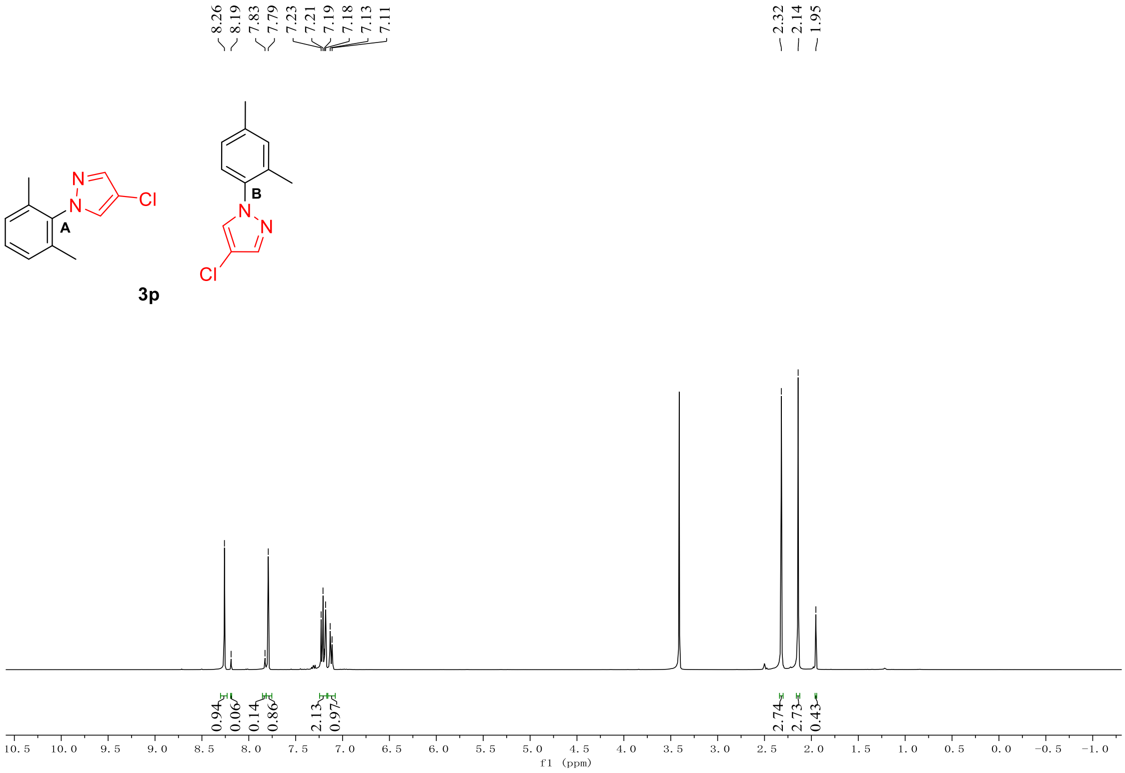

Supplementary Figure 31. ¹H NMR (400 MHz, DMSO) spectrum of 3p

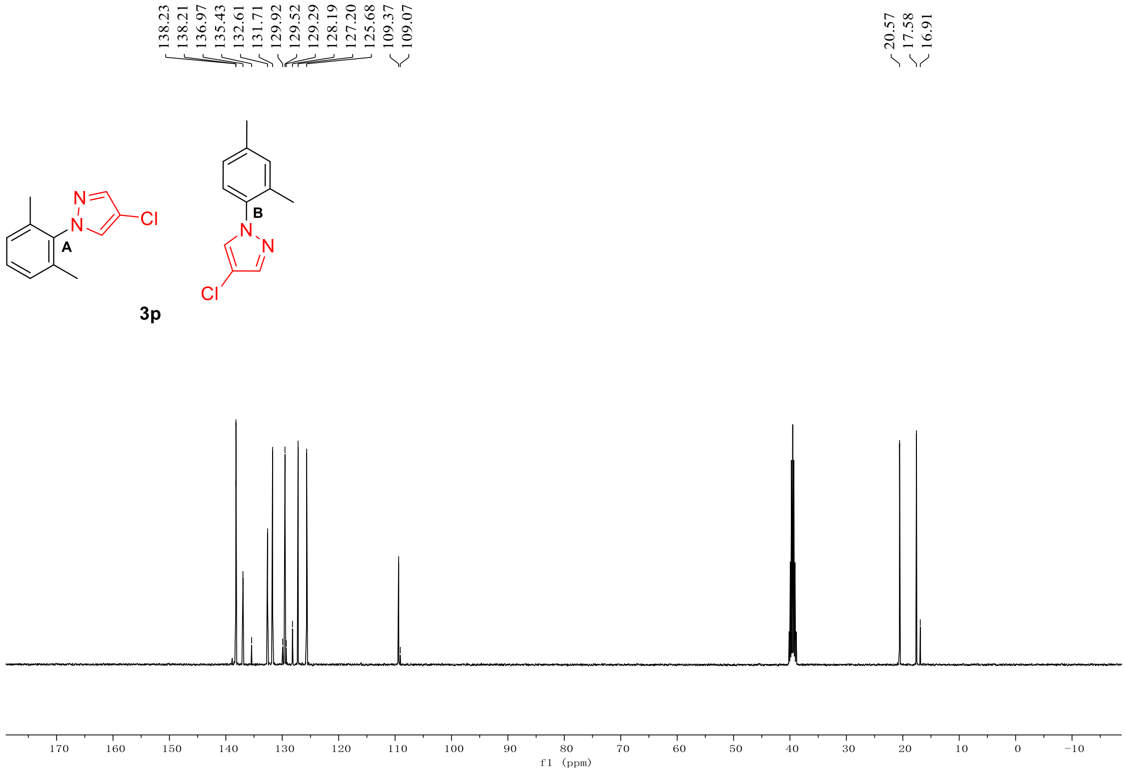

Supplementary Figure 32. ¹³C NMR (101MHz, DMSO) spectrum of 3p

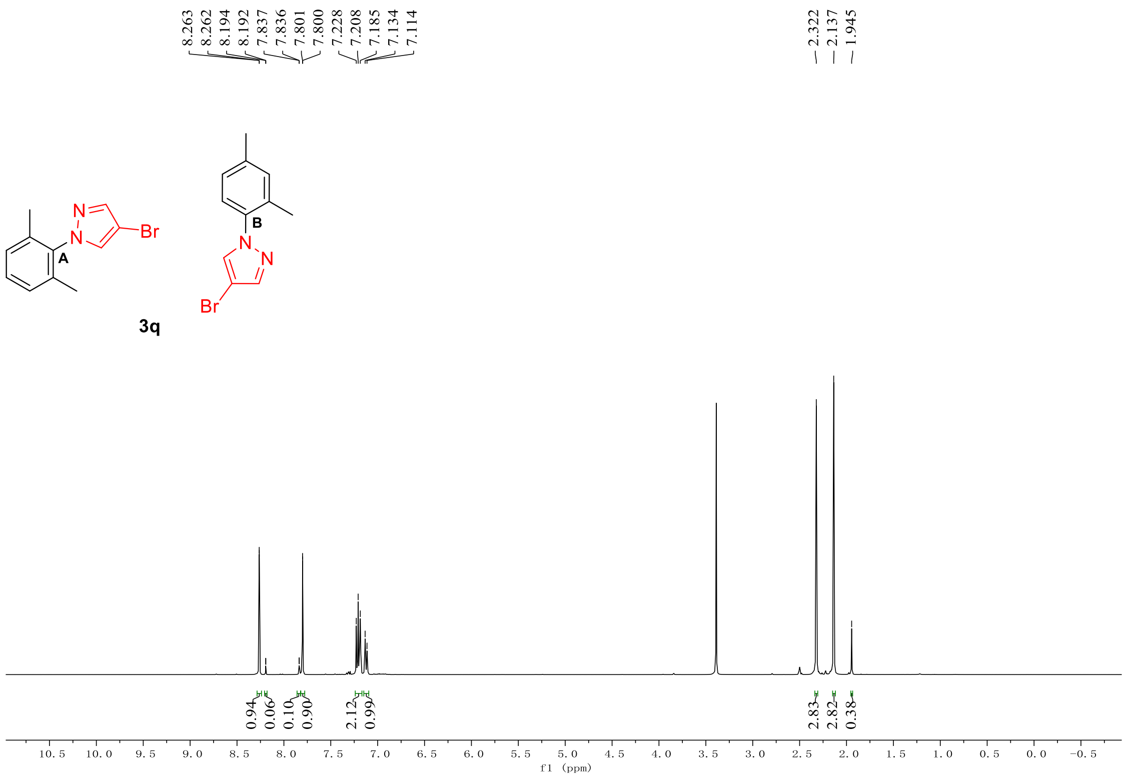

Supplementary Figure 33. ^{1}H NMR (400 MHz, DMSO) spectrum of 3q

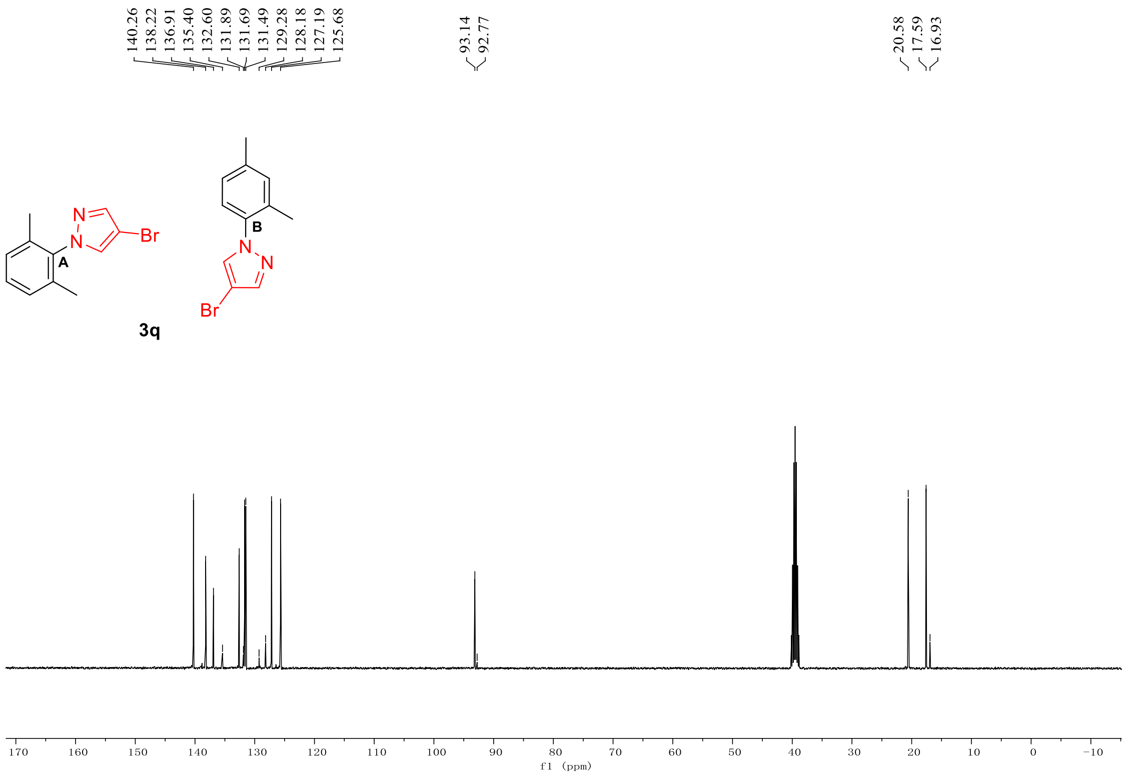

Supplementary Figure 34. ^{13}C NMR (101MHz, DMSO) spectrum of 3q

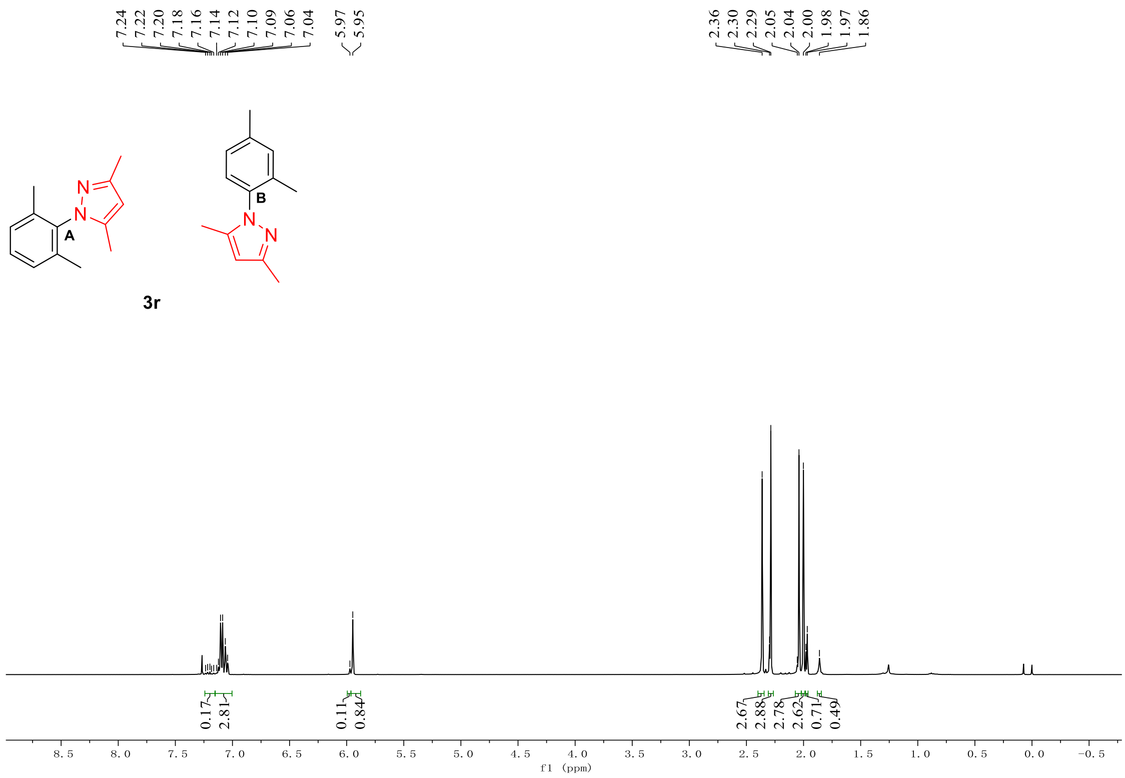

Supplementary Figure 35. ^{1}H **NMR (400 MHz,** $CDCl_3$**) spectrum of 3r**

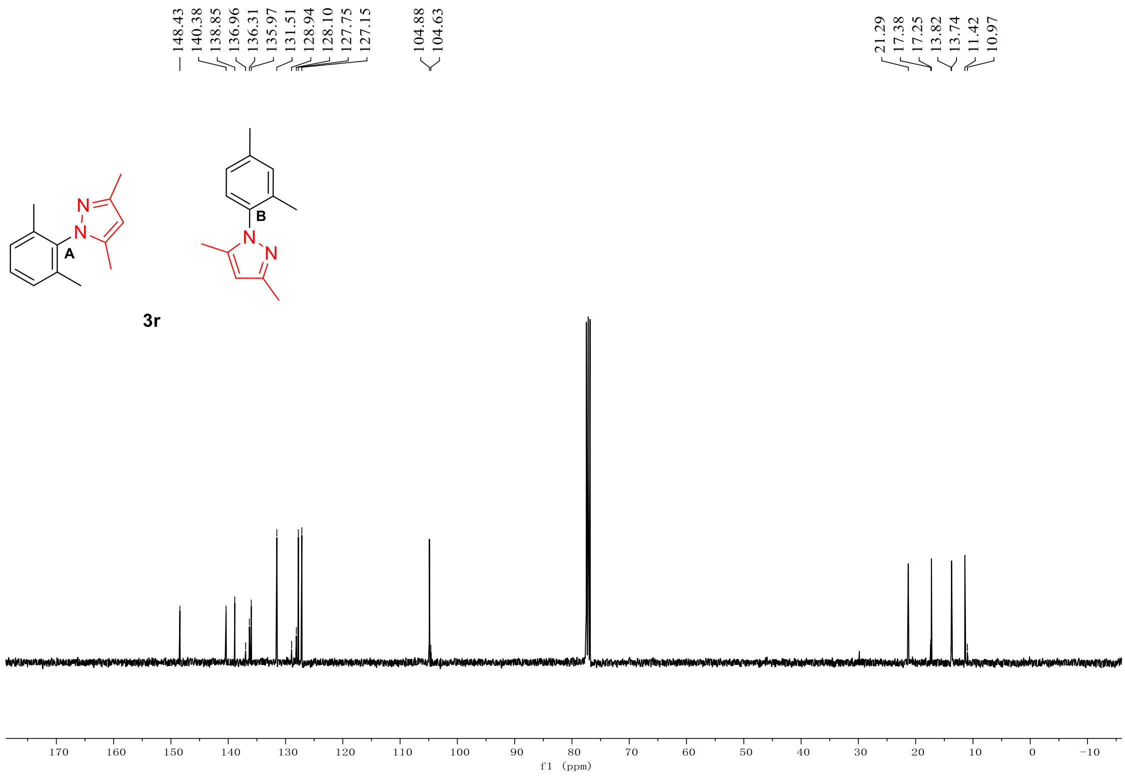

Supplementary Figure 36. ^{13}C **NMR (101MHz,** $CDCl_3$**) spectrum of 3r**

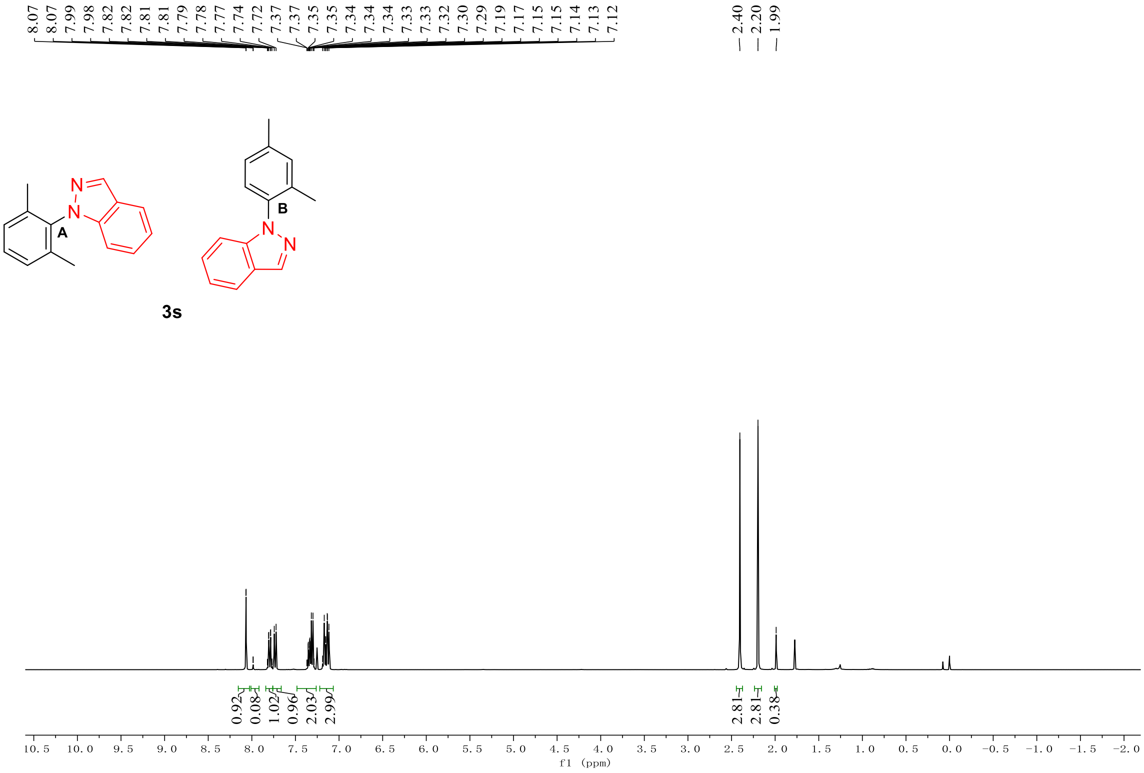

Supplementary Figure 37. ^{1}H NMR (400 MHz, $CDCl_3$) spectrum of 3s

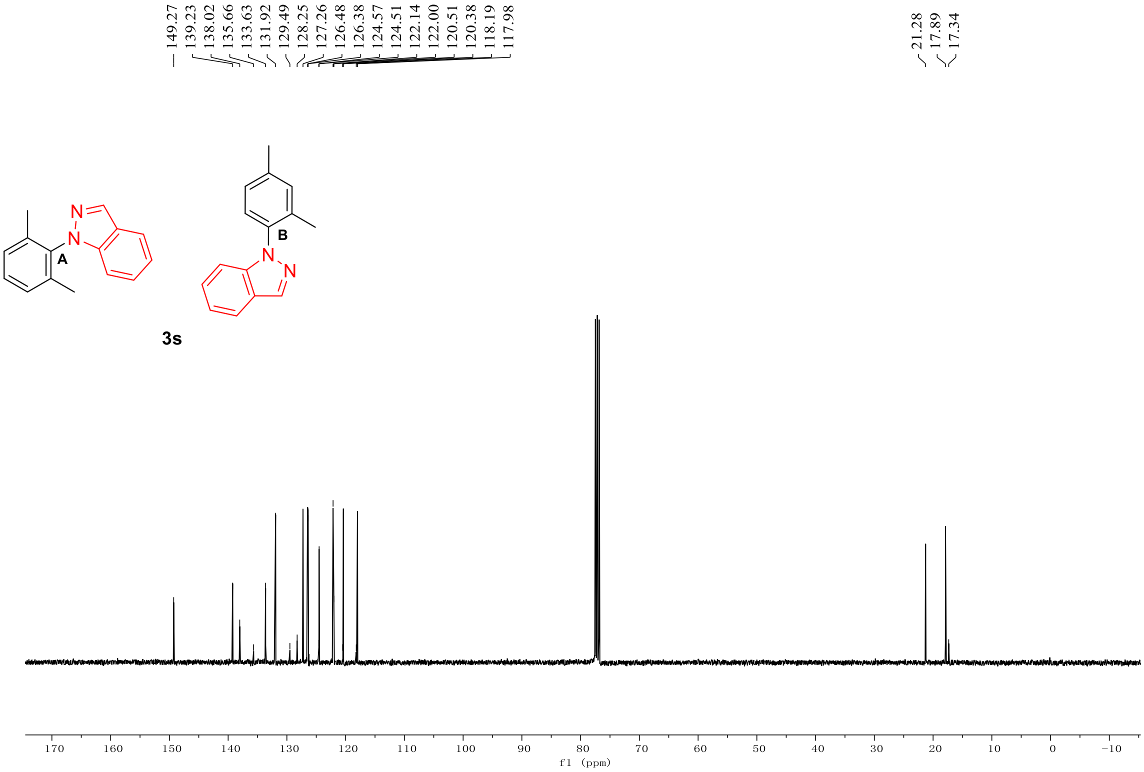

Supplementary Figure 38. ^{13}C NMR (101MHz, $CDCl_3$) spectrum of 3s

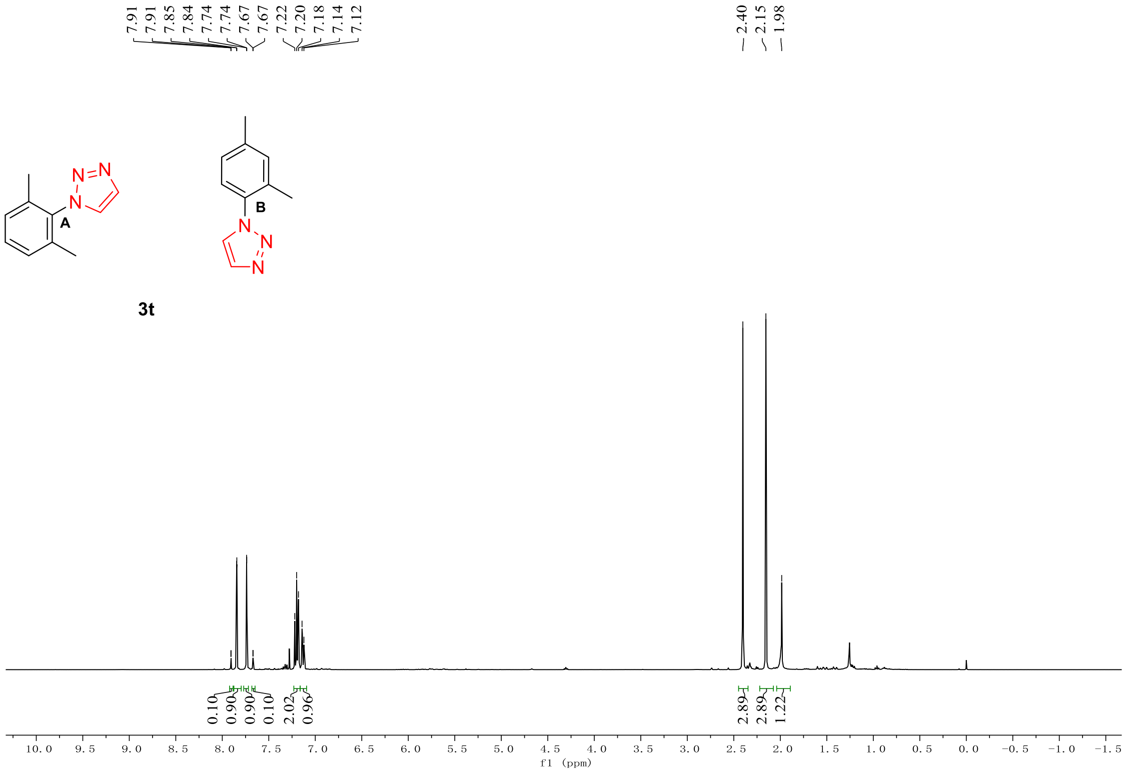

Supplementary Figure 39. ^{1}H NMR (400 MHz, $CDCl_3$) spectrum of 3t

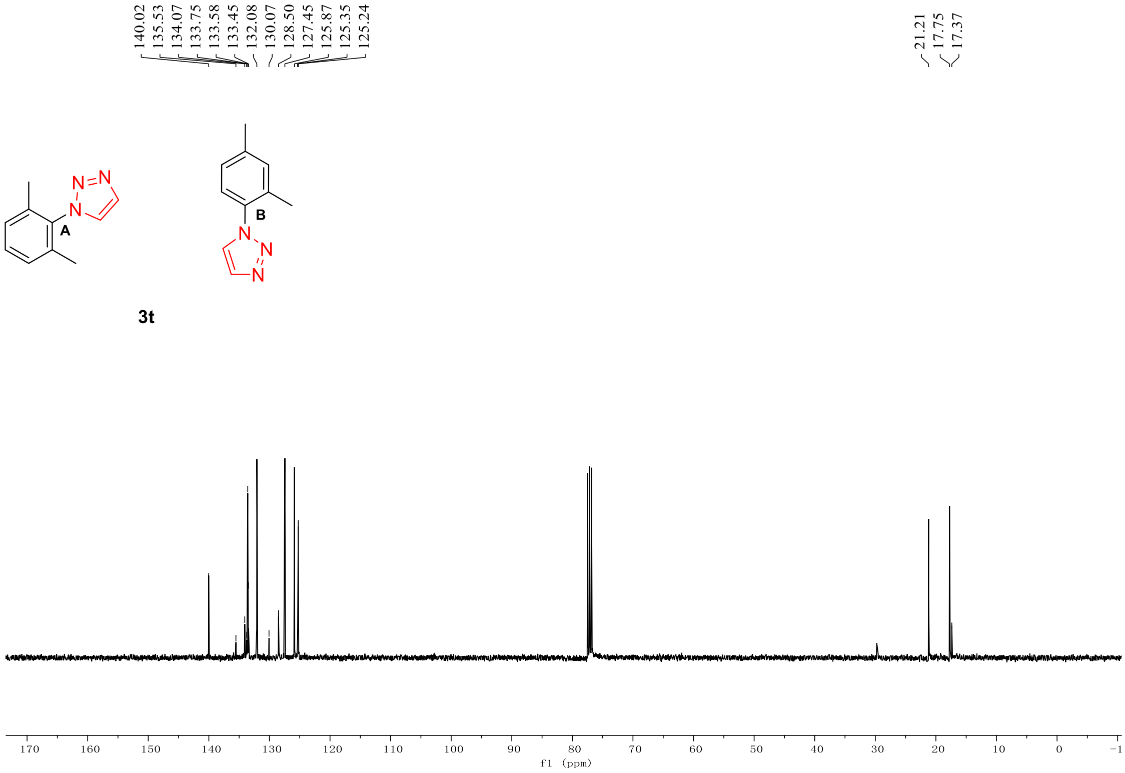

Supplementary Figure 40. ^{13}C NMR (101MHz, $CDCl_3$) spectrum of 3t

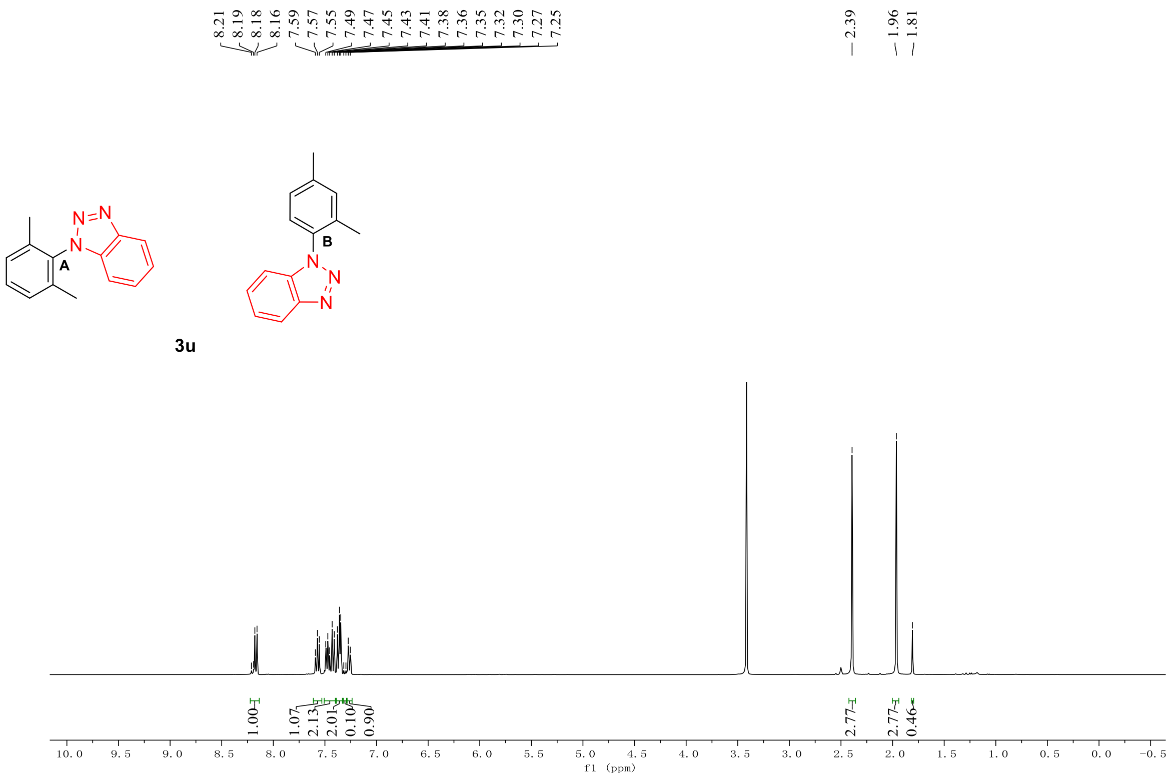

Supplementary Figure 41. ^{1}H NMR (400 MHz, DMSO) spectrum of 3u

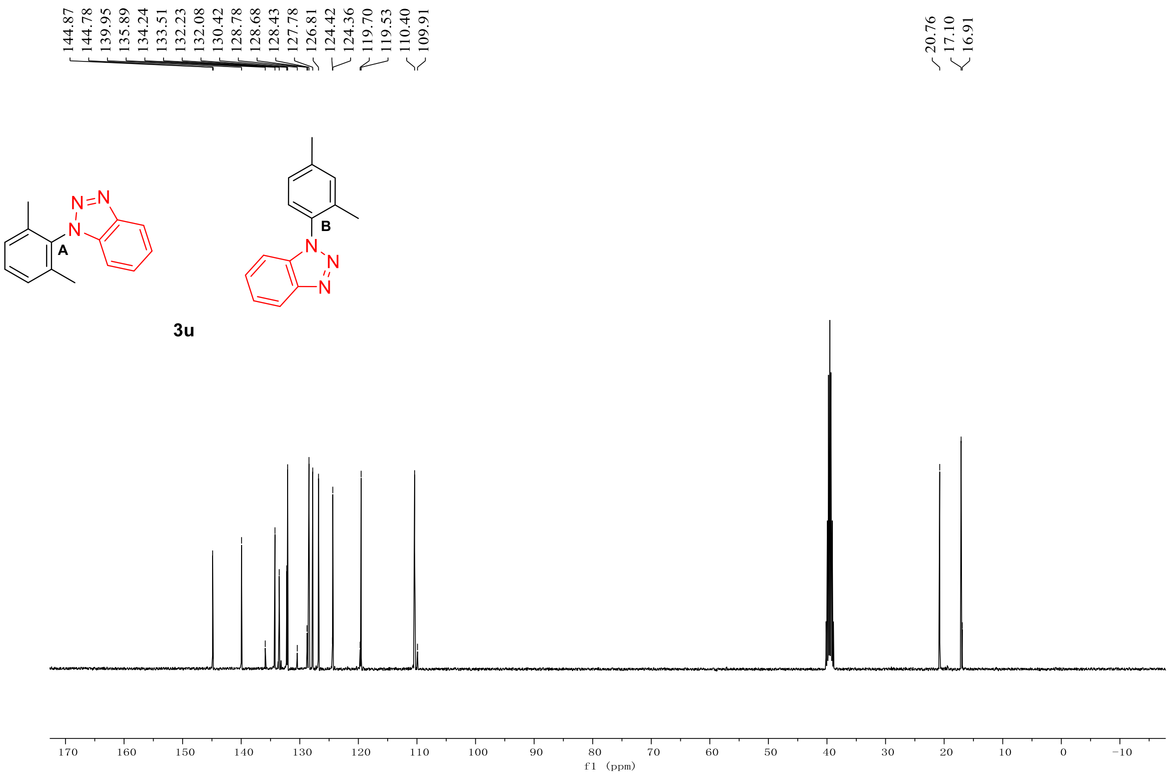

Supplementary Figure 42. ^{13}C NMR (101MHz, DMSO) spectrum of 3u

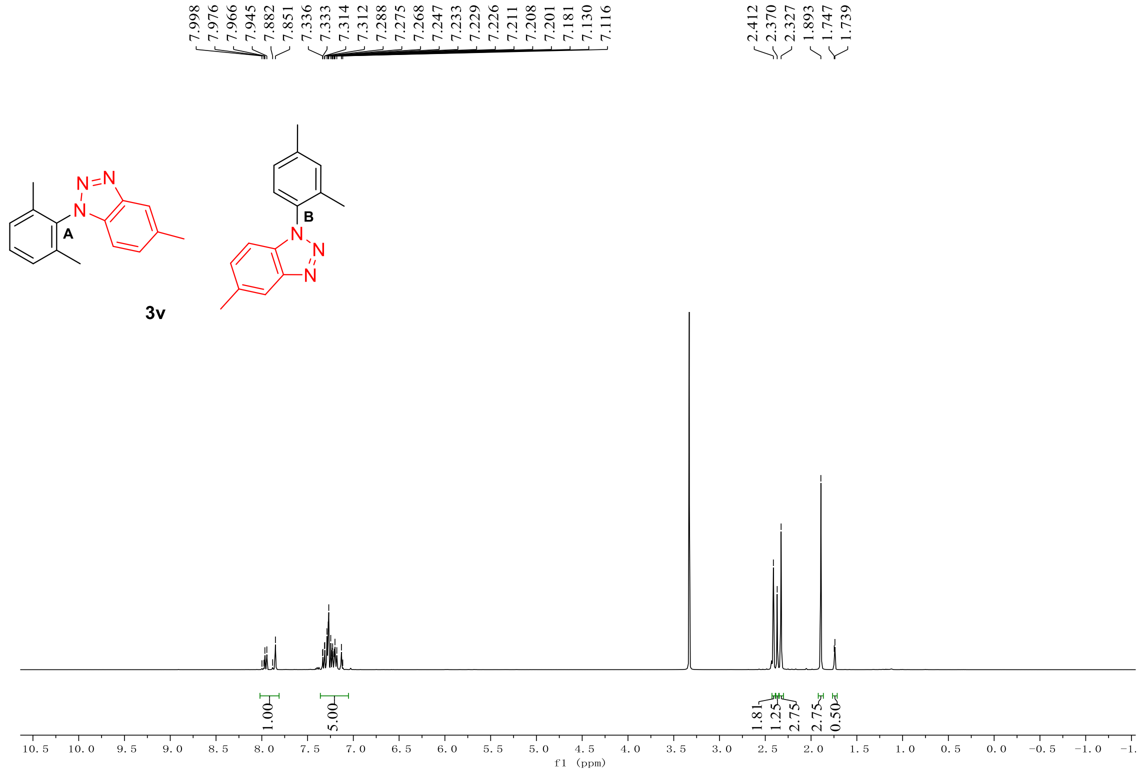

Supplementary Figure 43. ^{1}H NMR (400 MHz, DMSO) spectrum of 3v

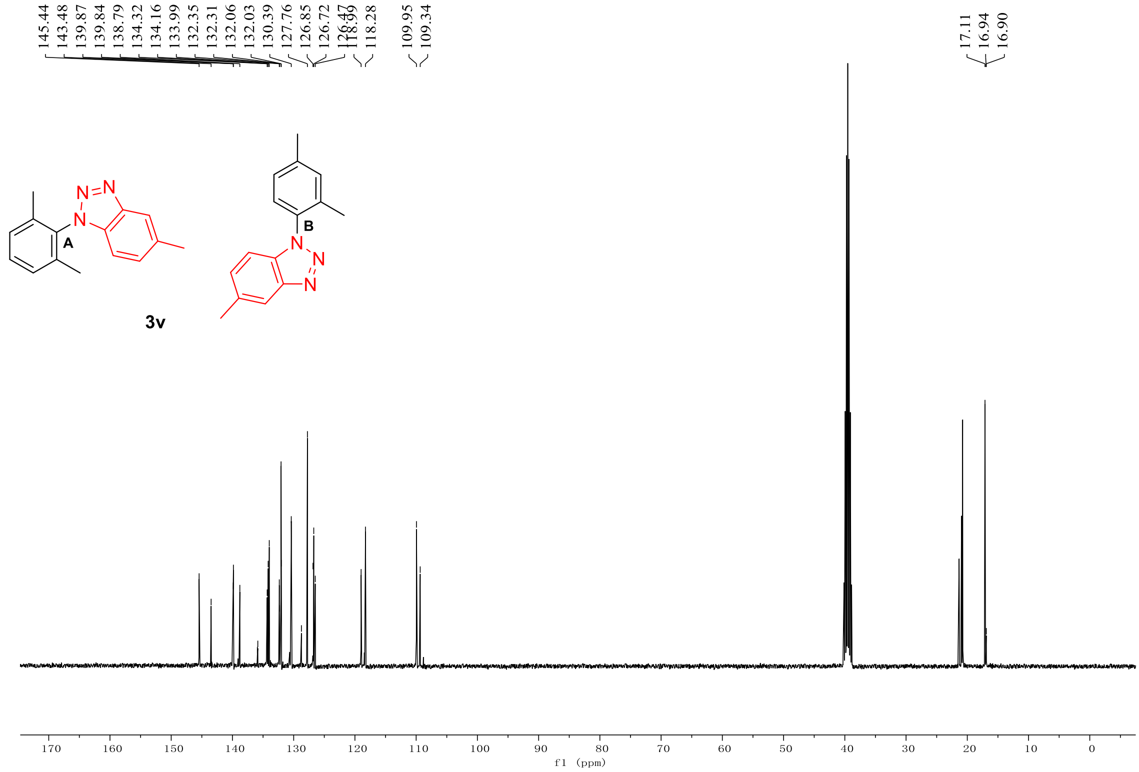

Supplementary Figure 44. ^{13}C NMR (101MHz, DMSO) spectrum of 3v

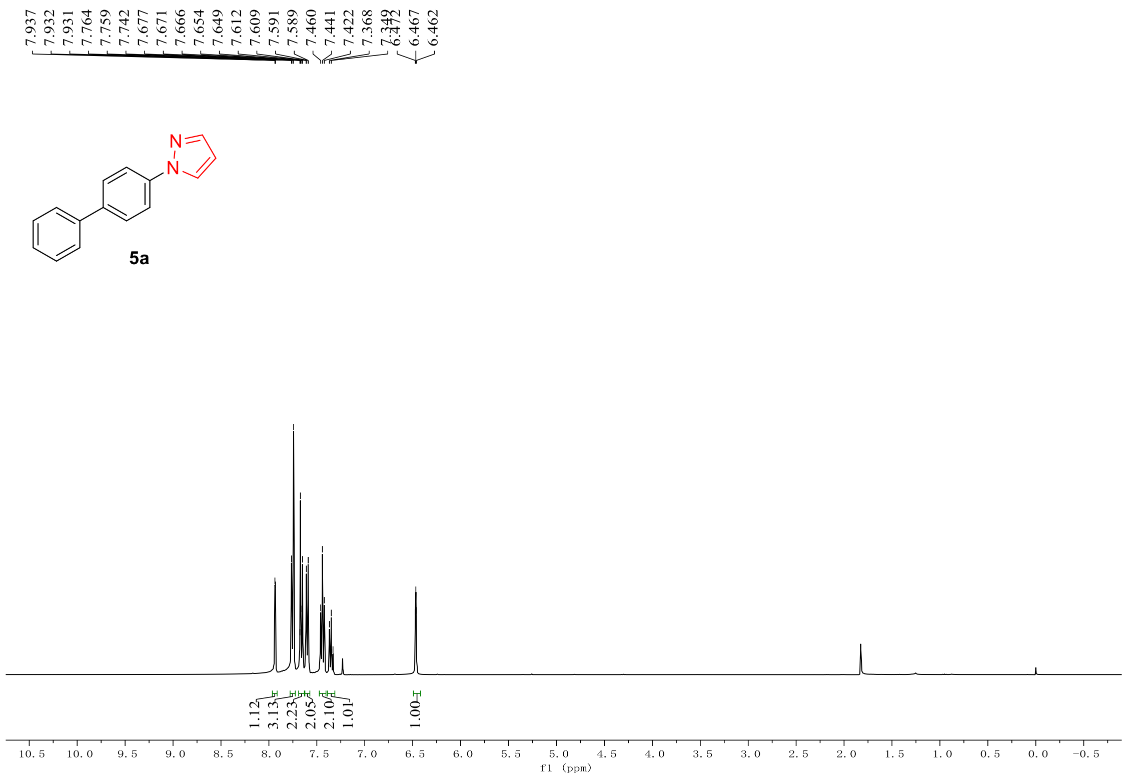

Supplementary Figure 45. ^{1}H NMR (400 MHz, $CDCl_3$) spectrum of 5a

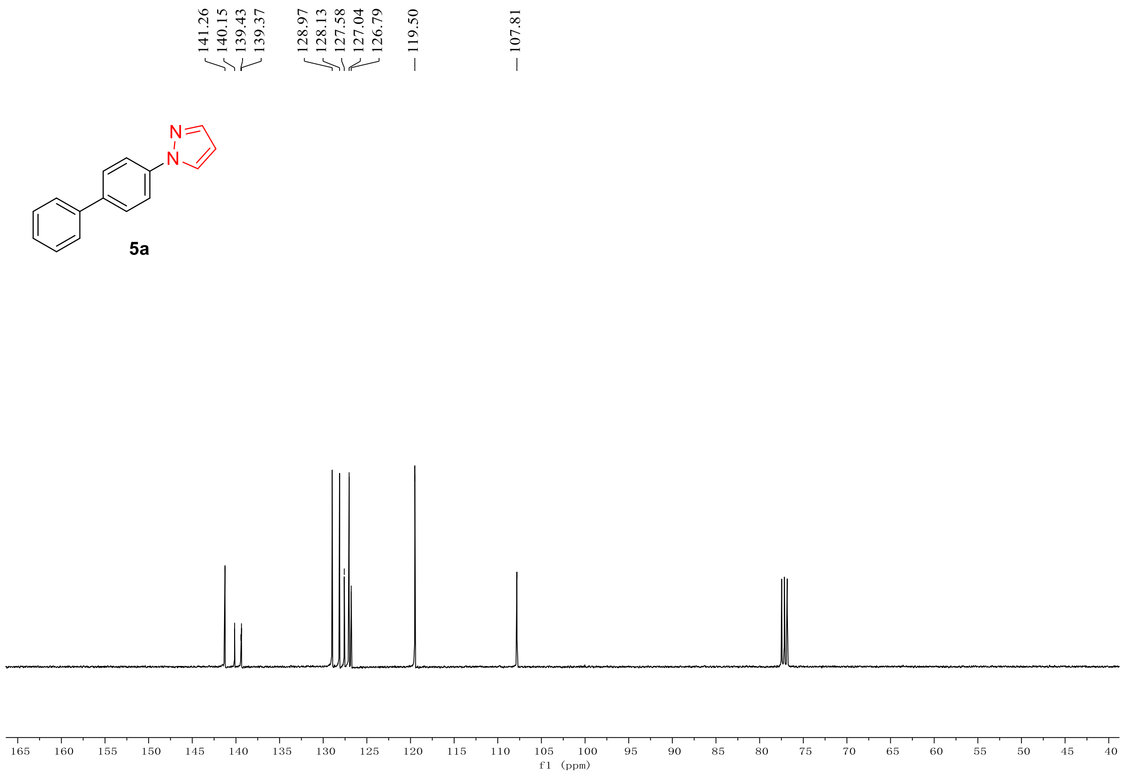

Supplementary Figure 46. ^{13}C NMR (101MHz, $CDCl_3$) spectrum of 5a

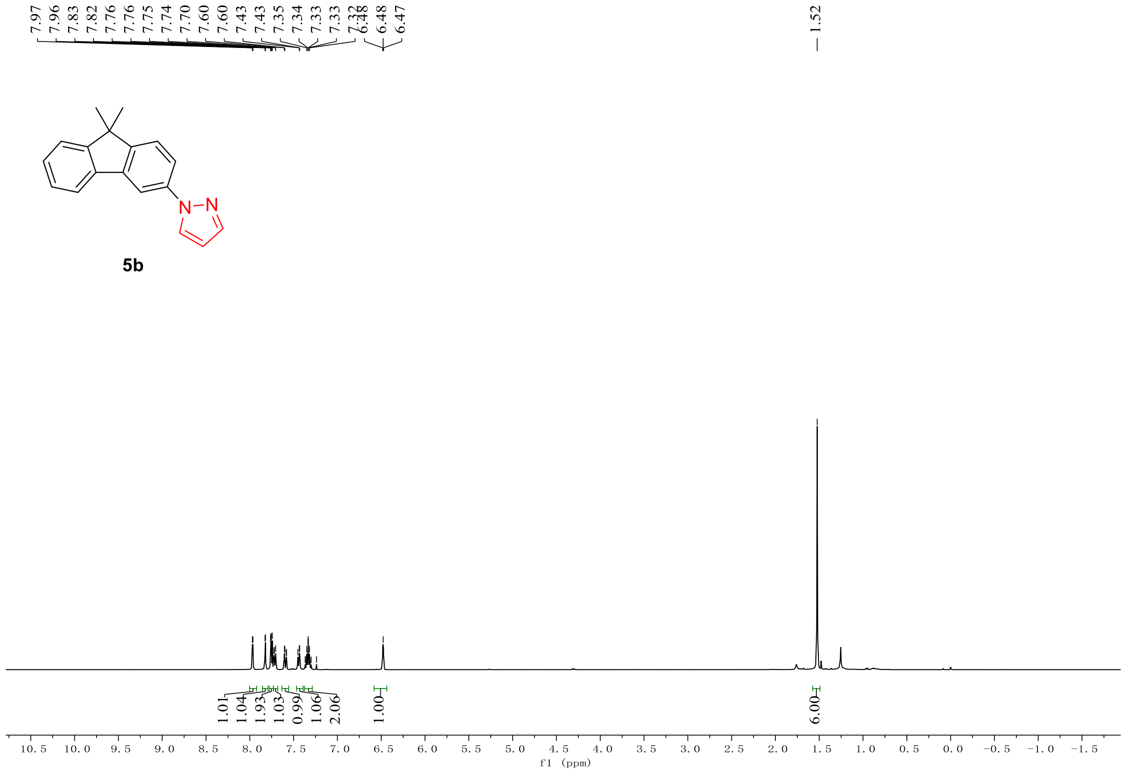

Supplementary Figure 47. ^{1}H NMR (400 MHz, $CDCl_3$) spectrum of 5b

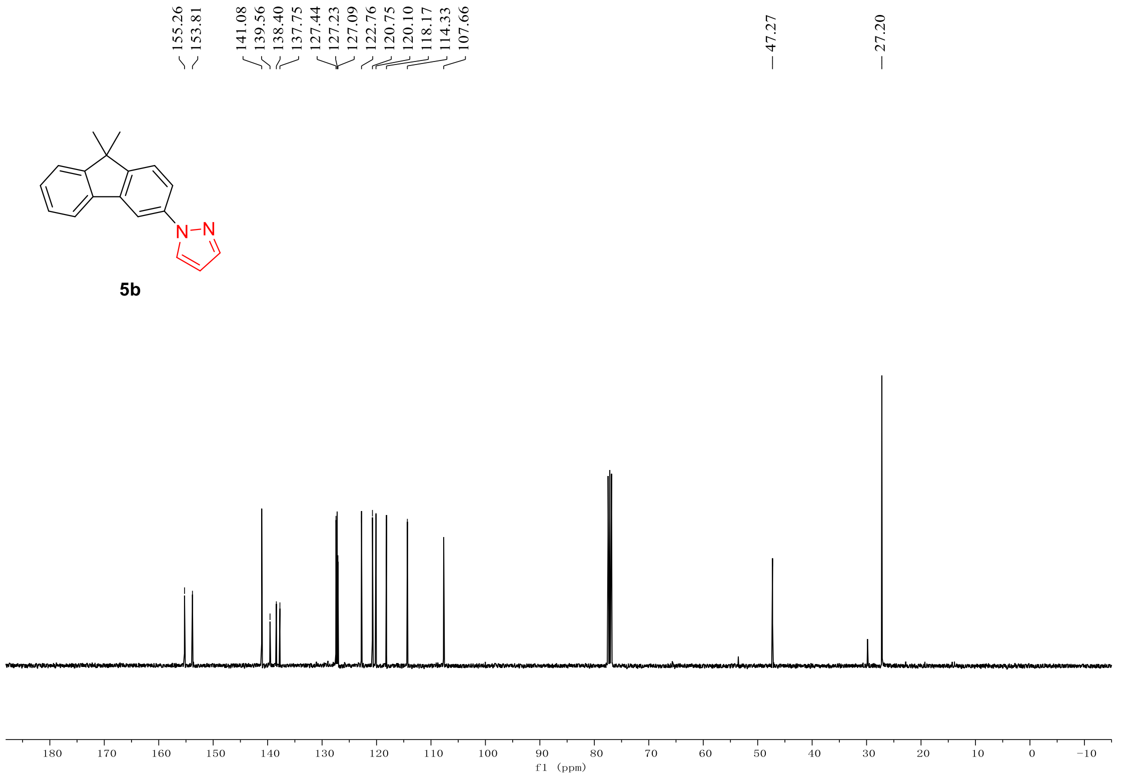

Supplementary Figure 48. ^{13}C NMR (101MHz, $CDCl_3$) spectrum of 5b

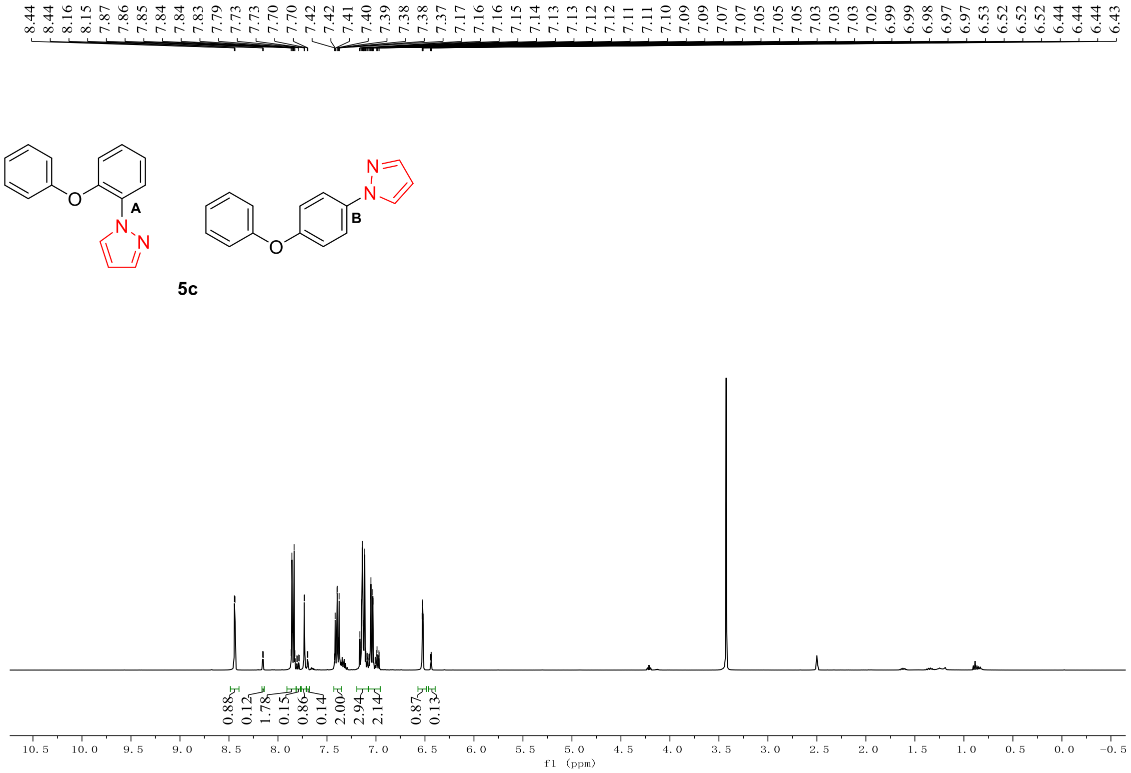

Supplementary Figure 49. ^{1}H NMR (400 MHz, DMSO) spectrum of 5c

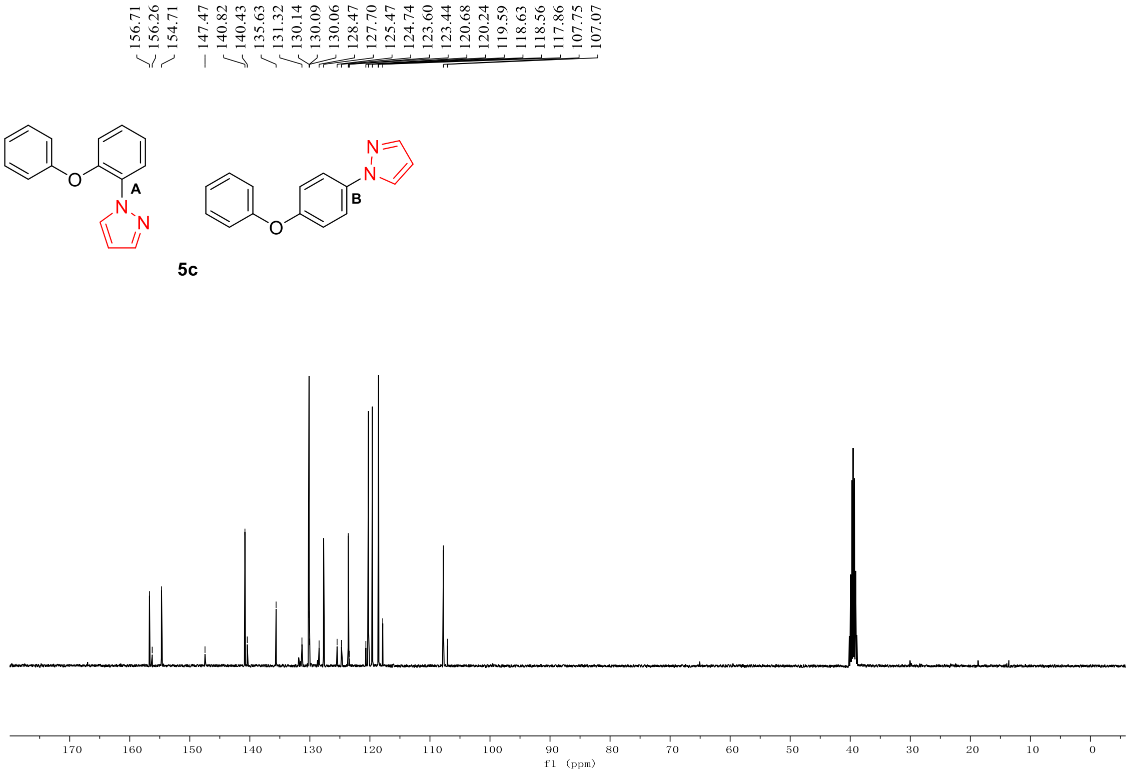

Supplementary Figure 50. ^{13}C NMR (101MHz, DMSO) spectrum of 5c

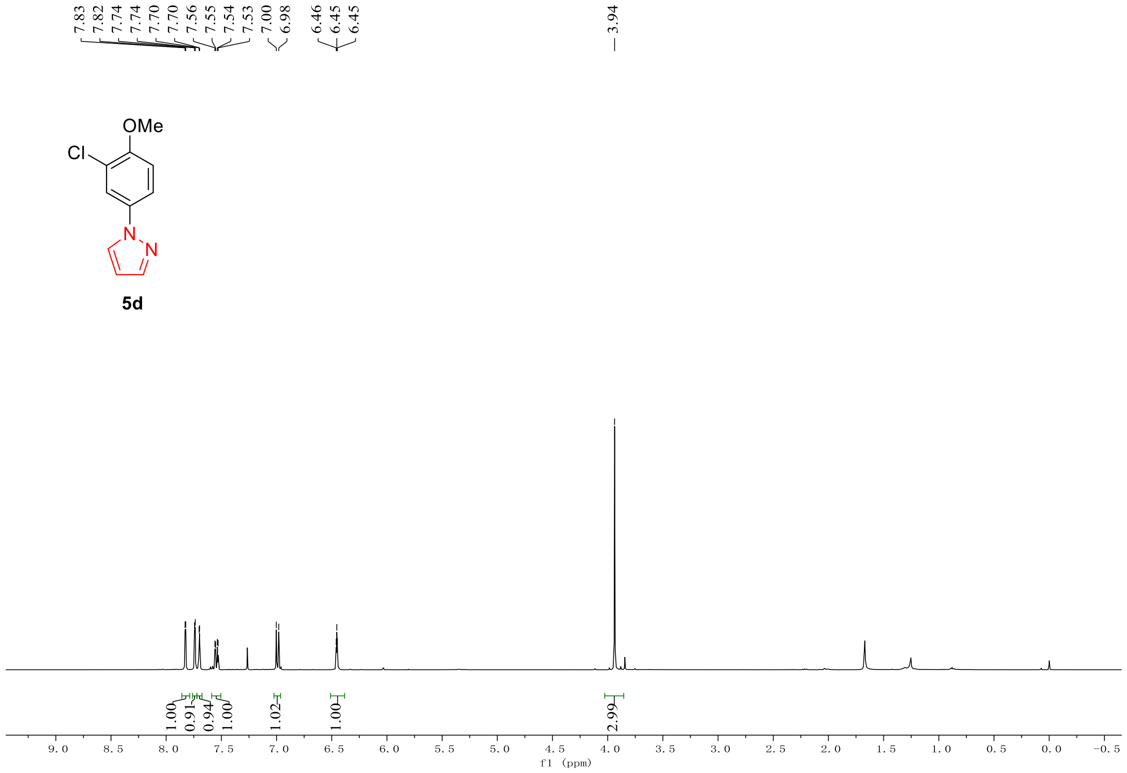

Supplementary Figure 51. ^{1}H NMR (400 MHz, $CDCl_3$) spectrum of 5d

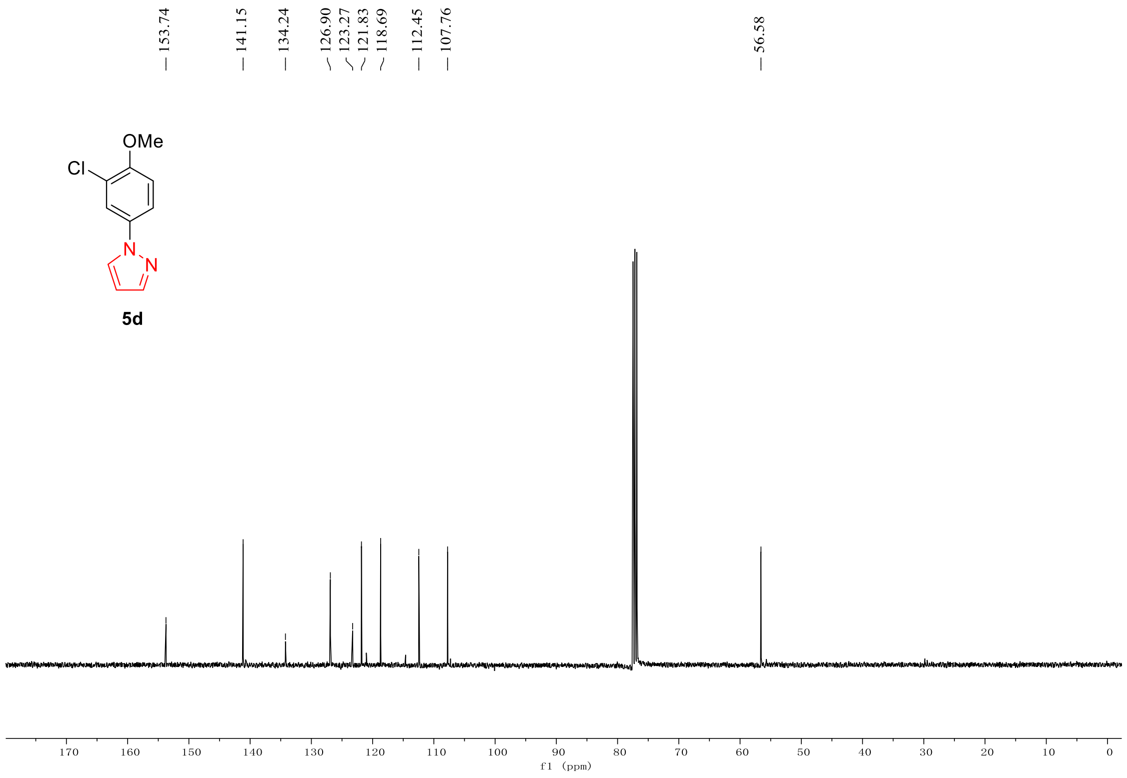

Supplementary Figure 52. ^{13}C NMR (101MHz, $CDCl_3$) spectrum of 5d

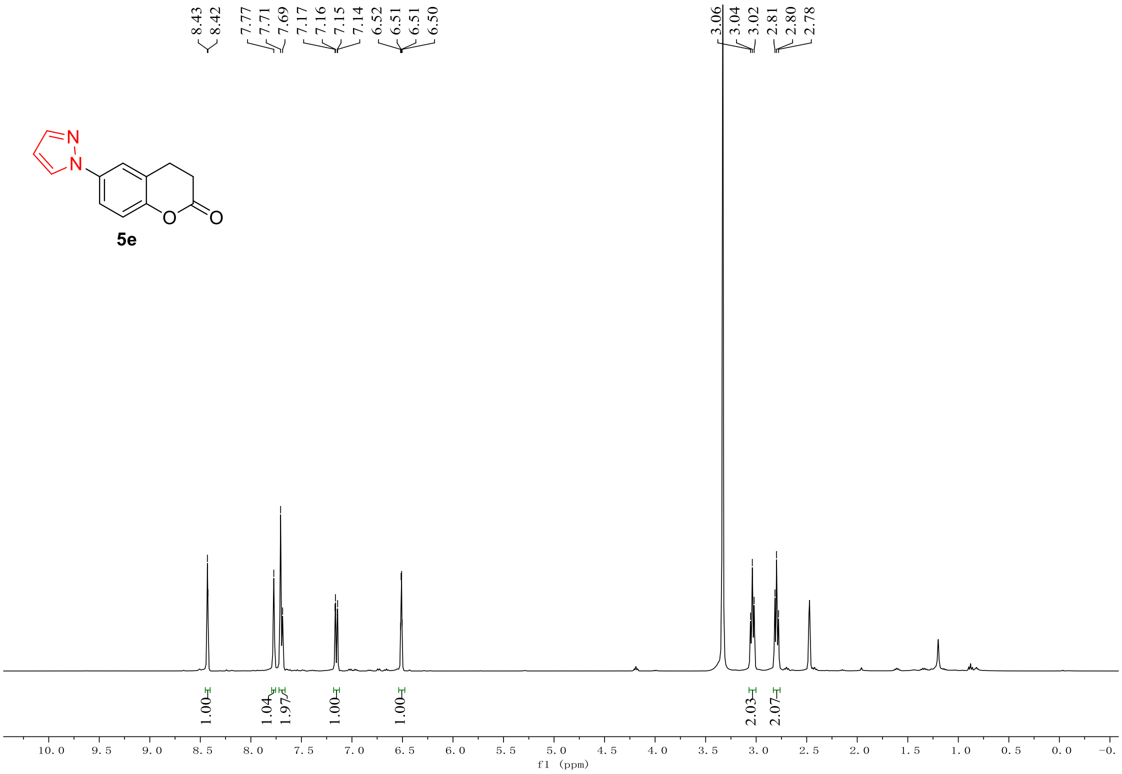

Supplementary Figure 49. ^{1}H NMR (400 MHz, DMSO) spectrum of 5e

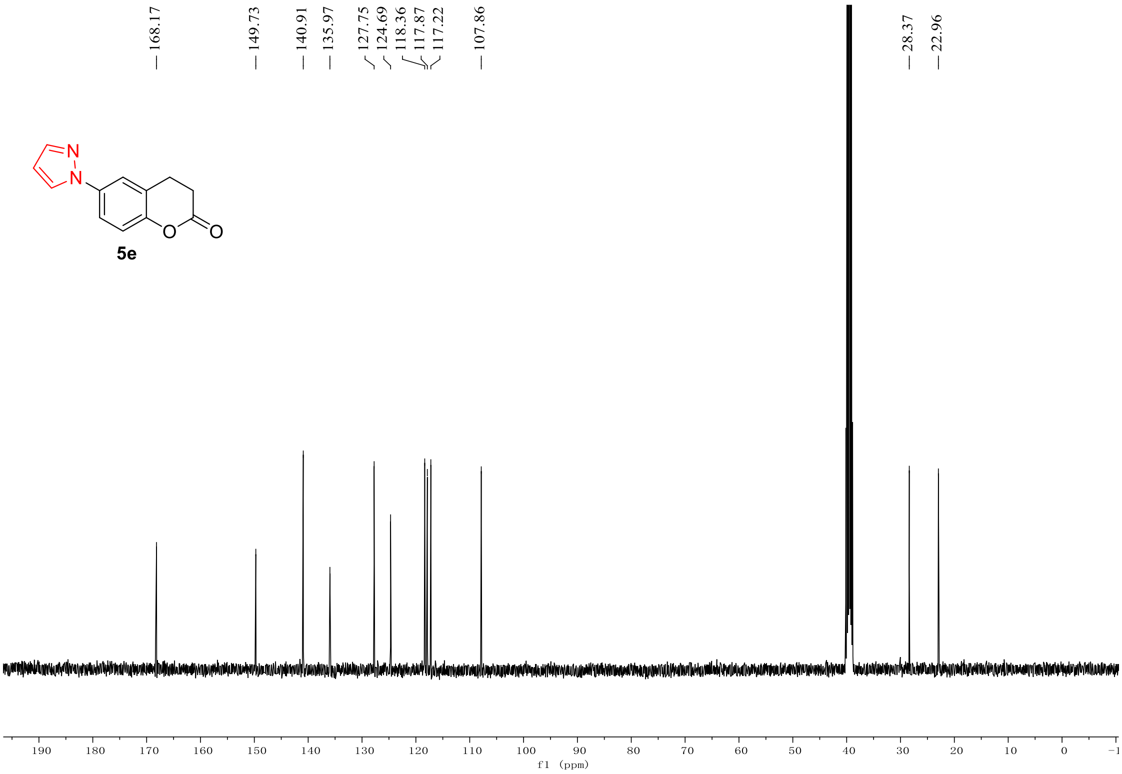

Supplementary Figure 54. ^{13}C NMR (101MHz, DMSO) spectrum of 5e

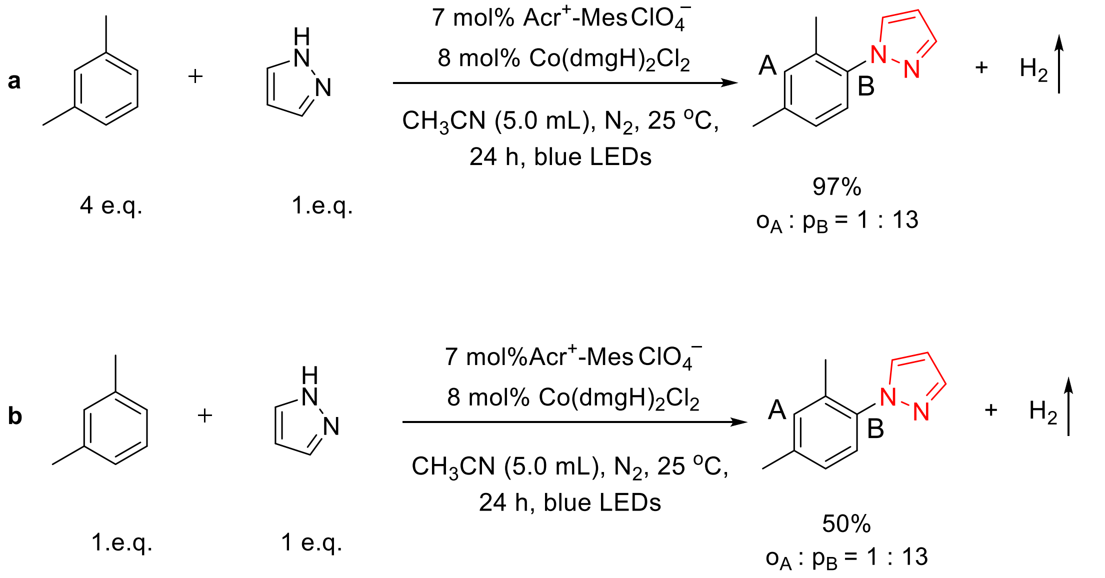

Supplementary Figure 55. The comparison of equivalents of aromatic hydrocarbons. (**a**) Reaction was proceeded with 1.2 mmol of *p*-xylene. (**b**) Reaction was proceeded with 0.3 mmol of *p*-xylene.

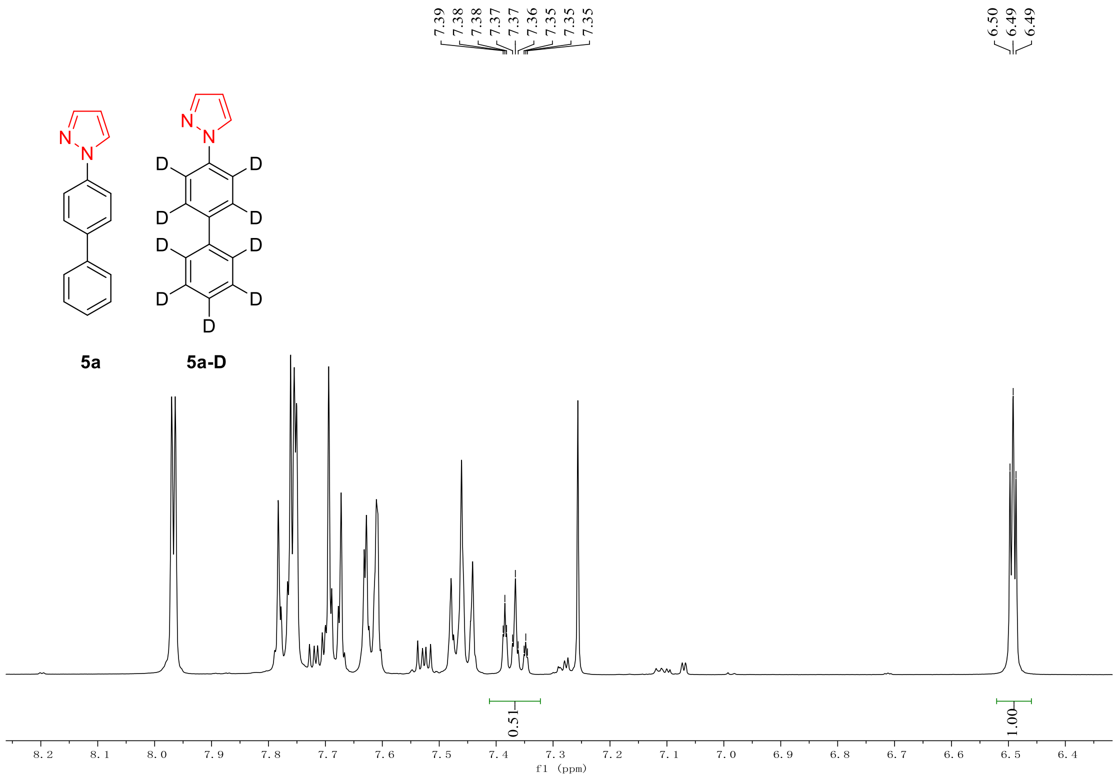

Supplementary Figure 56. ^{1}H NMR spectrum of desired product from KIE experiment

Supplementary Tables

Supplementary Table 1. Conditions screening

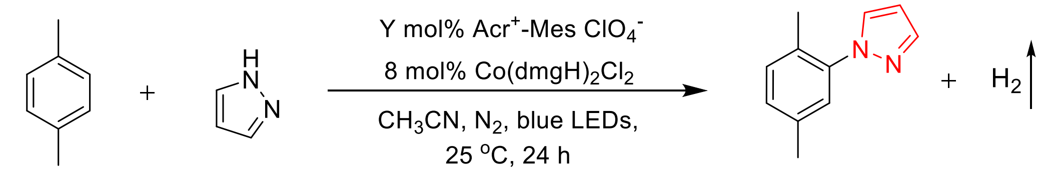

Entry	X	Y	CH_3CN (mL)	GC yield (%)
1	1	3	4.0	17
2	2	3	4.0	23
3	3	3	4.0	28
4	4	3	4.0	31
5	4	5	4.0	36
6	4	7	4.0	44
7	4	7	1.0	30
8	4	7	2.0	36
9	4	7	3.0	43
10	4	7	5.0	**65**

Supplementary Methods

General information: All manipulations were carried out by standard Schlenk techniques. Unless otherwise stated, analytical grade solvents and commercially available reagents were used to conduct the reactions. Thin layer chromatography (TLC) employed glass 0.25 mm silica gel plates. Flash chromatography columns were packed with 200-300 mesh silica gel in petroleum ether (bp. 60−90 ºC). Gradient flash chromatography was conducted eluting with a continuous gradient from petroleum ether to the ethyl acetate. All new compounds were characterized by ^{1}H NMR, ^{13}C NMR and HRMS. The known compounds were characterized by ^{1}H NMR and ^{13}C NMR. The ^{1}H and ^{13}C NMR spectra were recorded on a Bruker 400 MHz NMR spectrometer. High resolution mass spectra (HRMS) were measured with a Waters Micromass GCT instrument and accurate masses were reported for the molecular ion + Hydrogen (M+H). Hydrogen gas content was analyzed by gas chromatography (7890-II, Tianmei, China, TCD, nitrogen as a carrier gas and 5 Å molecular sieve column, a thermal conductivity detector).

Conditions Screening: A solution of *p*-xylene **1a** (X equiv), 1*H*-pyrazole **2a** (0.3 mmol), photocatalyst (Y mol%) and $Co^{III}(dmgH)_2Cl_2$ (8 mol%) in degassed dry CH_3CN were stirred under nitrogen atmosphere and irradiated by 3W blue LEDs at 25 ^oC for 24 h. After completion of the reaction, the yield of desired product **3a** was determined by GC with naphthalene as the internal standard. The results were summarized in Supplementary Table 1.

Reaction conditions for Substrate Scope: A solution of arenes **1** (1.2 mmol or 2.0 mL), azoles **2** (0.3 mmol), photocatalyst (7 mol%) and $Co^{III}(dmgH)_2Cl_2$ (8 mol%) in degassed dry CH_3CN (5.0 mL or 3.0 mL) were stirred under nitrogen atmosphere and irradiated by 3W blue LEDs at 25 ^oC for 24 h. After completion of the reaction, H_2 was detected by GC-TCD. The aqueous solution was extracted with ethyl acetate (3 × 10 mL) and the combined extracts were dried with anhydrous Na_2SO_4. The solvent was removed under reduced pressure by rotary evaporation. Then, the pure product was obtained by flash column chromatography on silica gel (eluent: petroleum ether/ethyl acetate= 25:1).

Procedure for Kinetic Isotope Effect (KIE) Experiment: A solution of biphenyl (0.6 mmol) and 1,1'-biphenyl-d_{10} (0.6 mmol), 1*H*-pyrazole **2a** (0.3 mmol), Acr^+-Mes ClO_4^- (7 mol%) and $Co^{III}(dmgH)_2Cl_2$ (8 mol%) in degassed dry CH_3CN (5.0 mL) were stirred under nitrogen atmosphere and irradiated by 3W blue LEDs at 25 ^oC for 1h. After completion of the reaction, H_2 was detected by GC-TCD. The aqueous solution was extracted with ethyl acetate (3 × 10 mL) and the combined extracts were dried with anhydrous Na_2SO_4. The solvent was removed under reduced pressure by rotary evaporation. Then, the pure product was obtained by flash column chromatography on silica gel (eluent: petroleum ether/ethyl acetate= 25:1) to afford 15% combined amination products. The ratio was K_H: K_D =1.04 determined by ^1H NMR spectrum. (See Supplementary Fig. 56).

Procedure for Kinetic Order in biphenyl substrate: The order in substrate arene was determined

by studying the initial rate of reaction with different concentrations of arene. Under nitrogen atmosphere, biphenyl (0.3 ~ 1.8 mmol, 0.3 mmol, 0.6mmol, 1.2 mmol, 1.5 mmol, 1.8 mmol), 1*H*-pyrazole **2a** (0.3 mmol), Acr^+-Mes ClO_4^- (0.021 mmol, 7 mol %), $Co^{III}(dmgH)_2Cl_2$ (8 mol%) and degassed dry CH_3CN (5.0 mL) were added to the reaction vessel one by one. The reaction was carried out under irradiation by a commercially available blue LEDs at 25 °C for 1 h. The GC yields were determined using naphthalene as an internal standard. Finally, the initial rate for different concentration of biphenyl vs relative concentrations could be obtained.

Analytical Data of Compounds

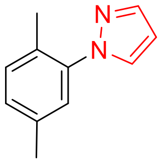

3a

Amination of *p*-xylene with pyrazole[1]: Reaction was proceeded with 1.2 mmol *p*-xylene and 5.0 mL CH_3CN. After 24 h, the reaction mixture was purified by silica gel column chromatography to afford the product **3a**. 36.2 mg colorless liquid (yield: 70%, 0.3 mmol scale).

^1H NMR (400 MHz, $CDCl_3$) δ 7.71 (d, *J* = 1.6 Hz, 1H), 7.59 (d, *J* = 2.3 Hz, 1H), 7.23 – 7.03 (m, 3H), 6.44 – 6.41 (m, 1H), 2.35 (s, 3H), 2.20 (s, 3H). ^{13}C NMR (101 MHz, $CDCl_3$) δ 140.24, 139.81, 136.50, 131.15, 130.59, 130.34, 129.15, 126.81, 106.17, 20.86, 17.74. HRMS (ESI) calcd for $C_{11}H_{13}N_2^+$, $[M+H]^+$, 173.1073, found 173.1072.

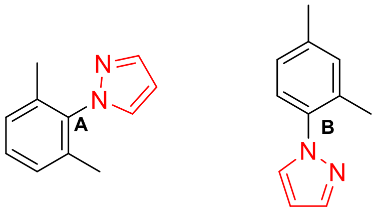

3b

Amination of *m*-xylene with pyrazole[2]: Reaction was proceeded with 1.2 mmol *m*-xylene and 5.0 mL CH_3CN. After 24 h, the reaction mixture was purified by silica gel column chromatography to afford the product **3b** as an inseparable mixture. The ratio of the mixture was 1:13 (C_A:C_B) as determined by ^1H NMR. 50.1 mg colorless liquid (yield: 97%, 0.3 mmol scale).

^{1}H NMR (400 MHz, $CDCl_3$) δ 7.73 (d, J = 1.7 Hz, 0.08H), 7.70 (d, J = 1.6 Hz, 0.91H), 7.55 (d, J = 2.3 Hz, 0.92H), 7.45 (d, J = 2.2 Hz, 0.08H), 7.27 – 7.17 (m, 1H), 7.12 (d, J = 8.7 Hz, 1H), 7.07 (d, J = 8.0 Hz, 1H), 6.45 – 6.43 (m, 0.08H), 6.42 – 6.40 (m, 0.92H), 2.36 (s, 2.88H), 2.18 (s, 2.88H), 2.00 (s, 0.44H). ^{13}C NMR (101 MHz, $CDCl_3$) δ 140.14, 140.10, 138.31, 137.64, 136.28, 133.51, 131.87, 130.75, 130.63, 129.01, 128.17, 127.16, 126.05, 106.06, 105.93, 21.13, 17.97, 17.37. HRMS (ESI) calcd for $C_{11}H_{13}N_2^+$, $[M+H]^+$, 173.1073, found 173.1068.

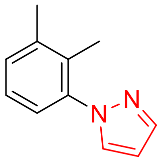

3c

Amination of *o*-xylene with pyrazole: Reaction was proceeded with 2.0 mL *o*-xylene and 3.0 mL CH_3CN. After 24 h, the reaction mixture was purified by silica gel column chromatography to afford the product **3c**. 36.4 mg colorless liquid (yield: 71%, 0.3 mmol scale).

^{1}H NMR (400 MHz, DMSO) δ 8.41 (d, J = 2.3 Hz, 1H), 7.70 (d, J = 1.4 Hz, 1H), 7.63 (d, J = 2.0 Hz, 1H), 7.53 (dd, J = 8.1, 2.3 Hz, 1H), 7.22 (d, J = 8.2 Hz, 1H), 6.51 – 6.49 (m, 1H), 2.27 (s, 3H), 2.23 (s, 3H). ^{13}C NMR (101 MHz, DMSO) δ 140.50, 137.78, 137.53, 134.21, 130.27, 127.42, 119.46, 115.72, 107.50, 19.58, 18.85. HRMS (ESI) calcd for $C_{11}H_{13}N_2^+$, $[M+H]^+$, 173.1073, found 173.1067.

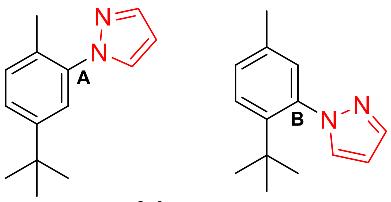

3d

Amination of 1-(*tert*-butyl)-4-methylbenzene with pyrazole: Reaction was proceeded with 1.2 mmol 1-(*tert*-butyl)-4-methylbenzene and 5.0 mL CH_3CN. After 24 h, the reaction mixture was purified by silica gel column chromatography to afford the product **3d** as an inseparable mixture. The ratio of the mixture was 19:1 (C_A:C_B) determined by ^{1}H NMR. 61.1 mg colorless liquid (yield: 95%, 0.3 mmol scale).

^{1}H NMR (400 MHz, DMSO) δ 8.08 – 7.98 (m, 0.95H), 7.90 – 7.83 (m, 0.06H), 7.80 – 7.61 (m, 0.95H), 7.65 – 7.63 (m, 0.05H), 7.48 (d, J = 8.2 Hz, 0.08H), 7.41 – 7.36 (m, 1H), 7.33 – 7.24 (m, 1.92H), 6.49 – 6.44 (m, 0.94H), 6.45 – 6.40 (m, 0,05H), 2.28 (s, 0.22H), 2.14 (s, 2.78H), 1.28 (s, 8.50H), 1.07 (s, 0.50H). ^{13}C NMR (101 MHz, DMSO) δ

149.43, 139.89, 139.39, 131.33, 130.91, 129.62, 125.07, 122.52, 106.21, 34.22, 31.04, 17.38. HRMS (ESI) calcd for $C_{14}H_{19}N_2^+$, $[M+H]^+$, 215.1543, found 215.1538.

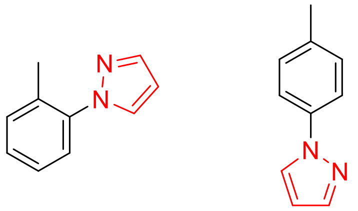

3e

Amination of toluene with pyrazole[1,3]: Reaction was proceeded with 2.0 mL toluene and 3.0 mL CH_3CN. After 24 h, the reaction mixture was purified by silica gel column chromatography to afford the product **3e** as an inseparable mixture. The ratio of the mixture was 1:14 (*ortho:para)* as determined by ^1H NMR. 38.0 mg pale yellow liquid (yield: 81%, 0.3 mmol scale).

^1H NMR (400 MHz, DMSO) δ 8.46 (d, J = 2.5 Hz, 0.10H), 8.43 (d, J = 2.5 Hz, 0.90H), 7.74 – 7.69 (m, 3H), 7.29 (d, J = 8.1 Hz, 2H), 6.54 – 6.49 (m, 1H), 2.38 (s, 0.20H), 2.33 (s, 2.80H). ^{13}C NMR (101 MHz, DMSO) δ 140.67, 137.59, 135.50, 129.94, 127.55, 118.36, 107.66, 20.49.

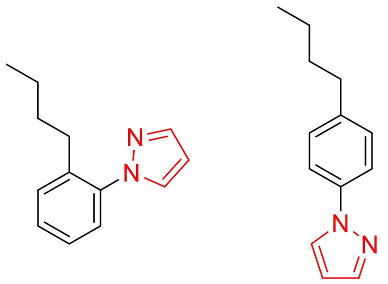

3f

Amination of *n*-butylbenzene with pyrazole: Reaction was proceeded with 2.0 mL of *n*-butylbenzene and 3.0 mL CH_3CN. After 24 h, the reaction mixture was purified by silica gel column chromatography to afford the product **3f** as an inseparable mixture. The ratio of the mixture was 1:8.6 (*ortho:para*) as determined by ^1H NMR. 42.1 mg colorless liquid (yield: 70%, 0.3 mmol scale).

^1H NMR (400 MHz, DMSO) δ 8.43 (d, J = 2.4 Hz, 0.10H), 8.38 (d, J = 2.4 Hz, 0.86H), 7.91 (d, J = 2.3 Hz, 0.27H), 7.73 – 7.63 (m, 2,88H), 7.35 – 7.32 (m, 0.62H), 7.24 (d, J = 8.5 Hz, 2H), 6.48 – 6.40 (m, 1H), 2.64 – 2.50 (m, 2H), 1.56 – 1.45 (m, 2H), 1.30 – 1.20 (m, 2H), 0.84 (t, J = 7.4 Hz, 2.58H), 0.69 (t, J = 7.3 Hz, 0.77H). ^{13}C NMR (101 MHz, DMSO) δ 140.66, 140.39, 139.94, 139.46, 138.06, 137.75, 131.50, 130.42, 129.26, 128.50, 127.54, 126.67,

126.40, 126.19, 118.38, 107.62, 106.30, 34.84, 34.20, 33.13, 33.06, 32.10, 30.55, 21.92, 21.74, 13.83, 13.64. HRMS (ESI) calcd for $C_{13}H_{17}N_2^+$, $[M+H]^+$, 201.1386, found 201.1382.

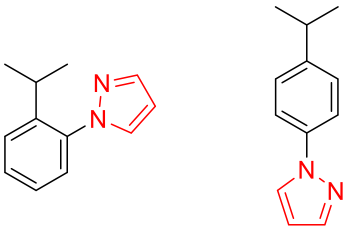

3g

Amination of cumene with pyrazole[4]: Reaction was proceeded with 2.0 mL cumene and 3.0 mL CH_3CN. After 24 h, the reaction mixture was purified by silica gel column chromatography to afford the product **3h** as an inseparable mixture. The ratio of the mixture was 1:6.1 (*ortho:para)* as determined by ^1H NMR. 40.7 mg colorless liquid (yield: 73%, 0.3 mmol scale).

^1H NMR (400 MHz, DMSO) δ 8.50 (d, *J* = 2.4 Hz, 0.14H), 8.43 (d, *J* = 2.4 Hz, 0.86H), 7.76 – 7.70 (m, 2.78H), 7.69 – 7.61 (m, 0.22H), 7.35 (d, *J* = 8.5 Hz, 2H), 6.55 – 6.50 (m, 1H), 2.93 (hept, *J* = 7.1 Hz, 1H), 1.33 – 1.11 (m, 6H). ^{13}C NMR (101 MHz, DMSO) δ 146.44, 140.67, 137.82, 127.59, 127.30, 118.49, 107.72, 107.61, 32.95, 23.89, 23.81. HRMS (ESI) calcd for $C_{12}H_{15}N_2^+$, $[M+H]^+$, 187.1230, found 187.1227.

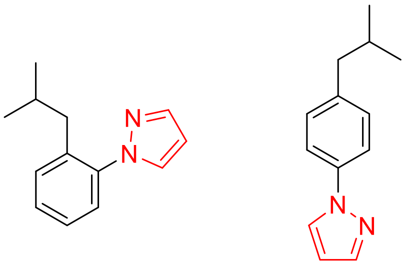

3h

Amination of isobutylbenzene with pyrazole: Reaction was proceeded with 2.0 mL isobutylbenzene and 3.0 mL CH_3CN. After 24 h, the reaction mixture was purified by silica gel column chromatography to afford the product **3g** as an inseparable mixture. The ratio of the mixture was 1:8.1 (*ortho:para)* as determined by ^1H NMR. 29.4 mg colorless liquid (yield: 49%, 0.3 mmol scale).

^1H NMR (400 MHz, DMSO) δ 8.48 (t, *J* = 2.5 Hz, 0.11H), 8.45 (d, *J* = 2.4 Hz, 0.89H), 7.75 – 7.69 (m, 2.82H), 7.67 – 7.63 (m, 0.18H), 7.27 (d, *J* = 8.5 Hz, 1.87H), 7.09 (d, *J* = 7.6 Hz, 0.1H), 6.55 – 6.49 (m, 1H), 2.48 (d, *J* = 7.2 Hz, 2H), 1.91 – 1.80 (m, 1H), 0.87 (d, *J* = 6.6 Hz, 6H). ^{13}C NMR (101 MHz, DMSO) δ 140.69, 139.19, 137.80, 129.92,

127.56, 118.24, 107.64, 43.92, 29.69, 22.18, 22.14. HRMS (ESI) calcd for $C_{13}H_{17}N_2^+$, $[M+H]^+$, 201.1386, found 201.1383.

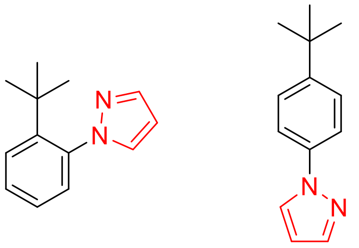

3i

Amination of *tert*-butylbenzene with pyrazole: Reaction was proceeded with 1.2 mmol *tert*-butylbenzene and 5.0 mL CH_3CN. After 24 h, the reaction mixture was purified by silica gel column chromatography to afford the product **3i** as an inseparable mixture. The ratio of the mixture was 1:4.6 (*ortho:para)* as determined by ^1H NMR. 57.1 mg colorless liquid (yield: 95%, 0.3 mmol scale).

^1H NMR (400 MHz, DMSO) δ 8.52 (d, J = 2.4 Hz, 0.18H), 8.44 (d, J = 2.4 Hz, 0.82H), 7.83 (t, J = 1.9 Hz, 0.21H), 7.77 – 7.69 (m, 2.58H), 7.66 – 7.60 (m, 0.29H), 7.59 (d, J = 2.1 Hz, 0.06H), 7.53 – 7.46 (m, 1.55H), 7.41 (t, J = 7.9 Hz, 0.21H), 7.35 – 7.31 (m, 0.22H), 7.25 (d, J = 8.2 Hz, 0.07H), 6.55 – 6.50 (m, 1H), 1.32 (s, 1.80H), 1.30 (s, 7.20H). ^{13}C NMR (101 MHz, DMSO) δ 152.38, 148.67, 140.77, 140.69, 137.48, 129.20, 127.85, 127.59, 126.20, 123.14, 118.17, 115.77, 115.51, 107.69, 107.61, 34.71, 34.27, 31.14, 31.07. HRMS (ESI) calcd for $C_{13}H_{17}N_2^+$, $[M+H]^+$, 201.1386, found 201.1382.

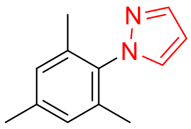

3j

Amination of mesitylene with pyrazole[2]: Reaction was proceeded with 1.2 mmol mesitylene and 5.0 mL CH_3CN. After 24 h, the reaction mixture was purified by silica gel column chromatography to afford the product **3j**. 51.4 mg colorless liquid (yield: 92%, 0.3 mmol scale).

^1H NMR (400 MHz, $CDCl_3$) δ 7.72 (d, J = 1.6 Hz, 1H), 7.47 – 7.36 (m, 1H), 6.94 (s, 2H), 6.43 (t, J = 2.1 Hz, 1H), 2.33 (s, 3H), 1.96 (s, 6H). ^{13}C NMR (101 MHz, $CDCl_3$) δ 140.02, 138.80, 136.96, 135.92, 130.91, 128.81, 105.82, 21.17, 17.28. HRMS (ESI) calcd for $C_{12}H_{15}N_2^+$, $[M+H]^+$, 187.1230, found 187.1232.

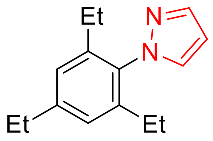

3k

Amination of 1,3,5-triethylbenzene with pyrazole: Reaction was proceeded with 1.2 mmol 1,3,5-triethylbenzene and 5.0 mL CH_3CN. After 24 h, the reaction mixture was purified by silica gel column chromatography to afford the product **3k**. 67.6 mg colorless liquid (yield: 99%, 0.3 mmol scale).

^{1}H NMR (400 MHz, $CDCl_3$) δ 7.71 (d, *J* = 1.7 Hz, 1H), 7.45 (d, *J* = 2.2 Hz, 1H), 7.00 (s, 2H), 6.42 (t, *J* = 2.1 Hz, 1H), 2.67 (q, *J* = 7.6 Hz, 2H), 2.31 – 2.13 (m, 4H), 1.26 (t, *J* = 7.6 Hz, 3H), 1.06 (t, *J* = 7.6 Hz, 6H). ^{13}C NMR (101 MHz, $CDCl_3$) δ 145.56, 142.00, 139.78, 136.01, 131.59, 126.02, 105.62, 28.86, 24.31, 15.72, 15.44. HRMS (ESI) calcd for $C_{15}H_{21}N_2^+$, $[M+H]^+$, 229.1699, found 229.1694.

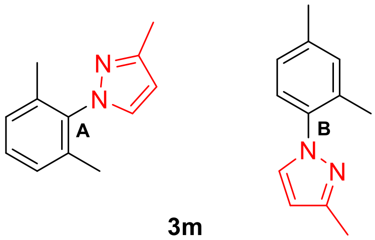

3m

Amination of *m*-xylene with 3-methyl-1*H*-pyrazole: Reaction was proceeded with 2.0 mL *m*-xylene and 3.0 mL CH_3CN. After 24 h, the reaction mixture was purified by silica gel column chromatography to afford the product **3l** as an inseparable mixture. The ratio of the mixture was 1:11 (C_A:C_B) as determined by ^{1}H NMR. 49.2 mg colorless liquid (yield: 88%, 0.3 mmol scale).

^{1}H NMR (400 MHz, DMSO) δ 7.80 (d, *J* = 2.2 Hz, 0.91H), 7.68 (d, *J* = 2.2 Hz, 0.09H), 7.29 – 7.24 (m, 0.13H), 7.20 – 7.13 (m, 2H), 7.11 – 7.07 (m, 0.88H), 6.25 – 6.23 (m, 1H), 2.31 (s, 2.75H), 2.24 (s, 3H), 2.16 (s, 2.75H), 1.94 (s, 0.50H). ^{13}C NMR (101 MHz, DMSO) δ 148.20, 148.00, 137.51, 137.12, 135.54, 132.23, 132.20, 131.88, 131.61, 128.62, 127.98, 127.04, 125.53, 105.90, 105.44, 20.55, 17.89, 17.07, 13.41. HRMS (ESI) calcd for $C_{12}H_{15}N_2^+$, [M+H]+, 187.1230, found 187.1225.

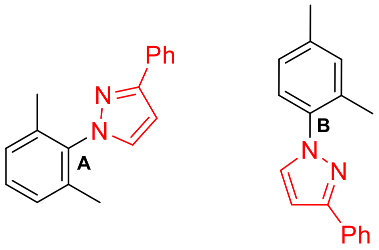

$3n_1$

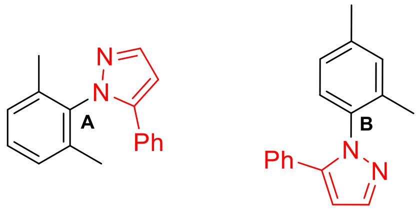

$3n_2$

Amination of *m*-xylene with 3-phenyl-1*H*-pyrazole: Reaction was proceeded with 2.0 mL *m*-xylene and 3.0 mL CH_3CN. After 24 h, the reaction mixture was purified by silica gel column chromatography to afford the N isomerous products **$3n_1$** 56.6 mg colorless liquid (yield: 76%, 0.3 mmol scale) and **$3n_2$** 17.1 mg colorless liquid (yield: 23%, 0.3 mmol scale). The *ortho:para* ratio of the **$3n_1$** was 1:8 (C_A:C_B) and the *ortho:para* ratio of the **$3n_2$** was 1:11.5 (C_A:C_B) as determined by ^1H NMR.

$3n_1$: ^1H NMR (400 MHz, $CDCl_3$) δ 7.94 – 7.83 (m, 2H), 7.57 (d, *J* = 2.4 Hz, 0.89H), 7.46 (d, *J* = 2.3 Hz, 0.11H), 7.40 (t, *J* = 7.5 Hz, 2H), 7.35 – 7.21 (m, 2H), 7.17 – 7.03 (m, 2H), 6.76 (d, *J* = 2.3 Hz, 0.10H), 6.72 (d, *J* = 2.4 Hz, 0.90H), 2.37 (s, 2.75H), 2.26 (s, 2.75H), 2.08 (s, 0.60H). ^{13}C NMR (101 MHz, $CDCl_3$) δ 152.16, 138.40, 137.72, 136.34, 133.57, 133.46, 132.30, 132.07, 132.00, 129.14, 128.70, 128.34, 127.85, 127.27, 126.07, 125.82, 103.46, 103.33, 21.19, 18.18, 17.57. HRMS (ESI) calcd for $C_{17}H_{17}N_2^+$, $[M+H]^+$, 249.1386, found 249.1381.

$3n_2$: ^1H NMR (400 MHz, DMSO) δ 7.79 (s, 0.08H), 7.72 (s, 0.92H), 7.28 (d, *J* = 5.4 Hz, 3H), 7.22 – 6.99 (m, 5H), 6.77 (s, 0.08H), 6.69 (s, 0.92H), 2.31 (s, 3H), 1.83 (d, *J* = 8.4 Hz, 3H). ^{13}C NMR (101 MHz, DMSO) δ 143.45, 139.83, 138.48, 136.92, 134.71, 131.39, 129.93, 128.59, 128.13, 127.85, 127.61, 127.24, 126.77, 106.04, 20.71, 17.16, 16.97. HRMS (ESI) calcd for $C_{17}H_{17}N_2^+$, $[M+H]^+$, 249.1386, found 249.1379.

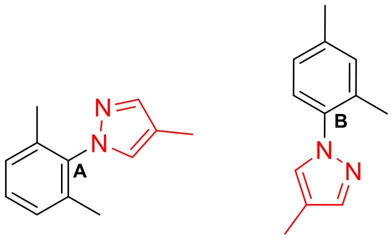

3o

Amination of *m*-xylene with 4-methyl-1*H*-pyrazole: Reaction was proceeded with 2.0 mL *m*-xylene and 3.0 mL CH_3CN. After 24 h, the reaction mixture was purified by silica gel column chromatography to afford the product **3o** as an inseparable mixture. The *ortho:para* ratio of the mixture was 1:14 (C_A:C_B) as determined by ^1H NMR. 50.3mg colorless liquid (yield: 90%, 0.3 mmol scale).

^{1}H NMR (400 MHz, DMSO) δ 7.69 (s, 0.93H), 7.57 (s, 0.07H), 7.51 (s, 0.08H), 7.49 (s, 0.87H), 7.20 – 7.13 (m, 2H), 7.11 – 7.07 (m, 1H), 2.31 (s, 2.80H), 2.16 (s, 2.80H), 2.11 – 2.07 (m, 3H), 1.93 (s, 0.40H). ^{13}C NMR (101 MHz, DMSO) δ 140.38, 140.20, 139.51, 137.57, 137.16, 135.47, 132.23, 131.66, 129.93, 129.64, 128.64, 128.00, 127.04, 125.38, 115.85, 115.44, 20.52, 17.91, 17.03, 8.66. HRMS (ESI) calcd for $C_{12}H_{15}N_2^+$, $[M+H]^+$, 187.1230, found 187.1226.

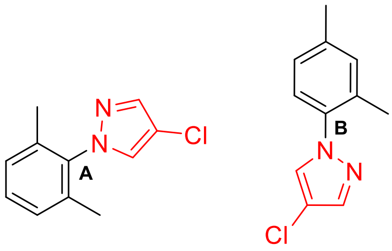

3p

Amination of *m*-xylene with 4-chloro-1*H*-pyrazole: Reaction was proceeded with 1.2 mmol *m*-xylene and 5.0 mL CH_3CN. After 24 h, the reaction mixture was purified by silica gel column chromatography to afford the product **3p** as an inseparable mixture. The ratio of the mixture was 1:15.7 (C_A:C_B) as determined by ^{1}H NMR. 58.9 mg colorless liquid (yield: 95%, 0.3 mmol scale).

^{1}H NMR (400 MHz, DMSO) δ 8.26 (s, 0.94H), 8.19 (s, 0.06H), 7.83 (s, 0.14H), 7.79 (s, 1.86H), 7.24 – 7.17 (m, 2H), 7.12 (d, J = 8.0 Hz, 1H), 2.32 (s, 2.74H), 2.14 (s, 2.73H), 1.95 (s, 0.43H). ^{13}C NMR (101 MHz, DMSO) δ 138.23, 138.21, 136.97, 135.43, 132.61, 131.71, 129.92, 129.52, 129.29, 128.19, 127.20, 125.68, 109.37, 109.07, 20.57, 17.58, 16.91. HRMS (ESI) calcd for $C_{11}H_{12}ClN_2^+$, $[M+H]^+$, 207.0684, found 207.0680.

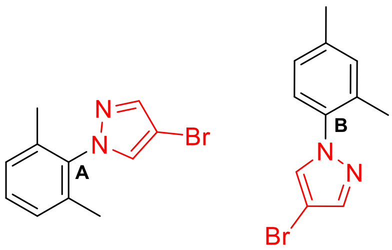

3q

Amination of *m*-xylene with 4-bromo-1*H*-pyrazole: Reaction was proceeded with 1.2 mmol *m*-xylene and 5.0 mL CH_3CN. After 24 h, the reaction mixture was purified by silica gel column chromatography to afford the product **3q** as an inseparable mixture. The ratio of the mixture was 1:15.7 (C_A:C_B) as determined by ^{1}H NMR. 73.8 mg colorless liquid (yield: 98%, 0.3 mmol scale).

^{1}H NMR (400 MHz, DMSO) δ 8.26 (d, *J* = 0.4 Hz, 0.94H), 8.19 (d, *J* = 0.7 Hz, 0.06H), 7.84 (d, *J* = 0.4 Hz, 0.10H), 7.80 (d, *J* = 0.4 Hz, 0.90H), 7.25 – 7.17 (m, 2H), 7.12 (d, *J* = 8.0 Hz, 1H), 2.32 (s, 2.83H), 2.14 (s, 2.82H), 1.95 (s, 0.38H). ^{13}C NMR (101 MHz, DMSO) δ 140.26, 138.22, 136.91, 135.40, 132.60, 131.89, 131.69, 131.49, 129.28, 128.18, 127.19, 125.68, 93.14, 92.77, 20.58, 17.59, 16.93. HRMS (ESI) calcd for $C_{11}H_{12}BrN_2^+$, $[M+H]^+$, 251.0178, found 251.0172.

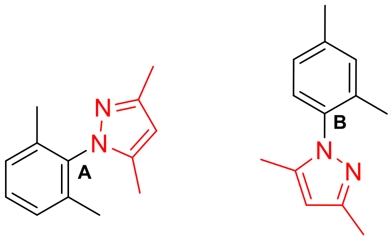

3r

Amination of *m*-xylene with 3,5-dimethyl-1*H*-pyrazole: Reaction was proceeded with 2.0 mL *m*-xylene and 3.0 mL CH_3CN. After 24 h, the reaction mixture was purified by silica gel column chromatography to afford the product **3r** as an inseparable mixture. The ratio of the mixture was 1:7.6 (C_A:C_B) as determined by ^{1}H NMR. 58.9 mg colrless liquid (yield: 98%, 0.3 mmol scale).

^{1}H NMR (400 MHz, $CDCl_3$) δ 7.24 – 7.16 (m, 0.17H), 7.15 – 7.01 (m, 2.81H), 5.97 (s, 0.11H), 5.95 (s, 0.84H), 2.36 (s, 2.67H), 2.29 (d, *J* = 4.5 Hz, 2.88H), 2.05 (d, *J* = 5.9 Hz, 2.78H), 2.00 (s, 2.62H), 1.97 (d, *J* = 4.7 Hz, 0.71H), 1.86 (s, 0.49H). ^{13}C NMR (101 MHz, $CDCl_3$) δ 148.43, 140.38, 138.85, 136.96, 136.31, 135.97, 131.51, 128.94, 128.10, 127.75, 127.15, 104.88, 104.63, 21.29, 17.38, 17.25, 13.82, 13.74, 11.42, 10.97. HRMS (ESI) calcd for $C_{13}H_{17}N_2^+$, $[M+H]^+$, 201.1386, found 201.1379.

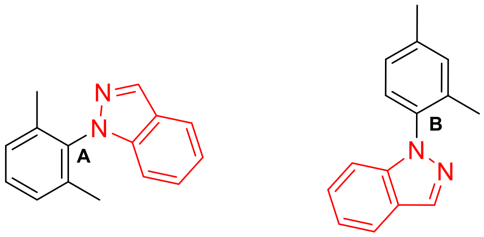

3s

Amination of *m*-xylene with 1*H*-indazole: Reaction was proceeded with 2.0 mL *m*-xylene and 3.0 mL CH_3CN. After 24 h, the reaction mixture was purified by silica gel column chromatography to afford the product **3s** as an inseparable mixture. The ratio of the mixture was 1:11.5 (C_A:C_B) as determined by ^{1}H NMR. 54.0 mg pale yellow liquid (yield: 81%, 0.3 mmol scale).

^1H NMR (400 MHz, $CDCl_3$) δ 8.08 – 8.06 (m, 0.92H), 8.00 – 7.97 (m, 0.08H), 7.83 – 7.76 (m, 1H), 7.73 (d, *J* = 8.5 Hz, 1H), 7.38 – 7.27 (m, 2H), 7.20 – 7.08 (m, 3H), 2.40 (s, 2.81H), 2.20 (s, 2.81H), 1.99 (s, 0.38H). ^{13}C NMR (101 MHz, $CDCl_3$) δ 149.27, 139.23, 138.02, 135.66, 133.63, 131.92, 129.49, 128.25, 127.26, 126.48, 126.38, 124.57, 124.51, 122.14, 122.00, 120.51, 120.38, 118.19, 117.98, 21.28, 17.89, 17.34. HRMS (ESI) calcd for $C_{15}H_{15}N_2^+$, $[M+H]^+$, 223.1230, found 223.1227.

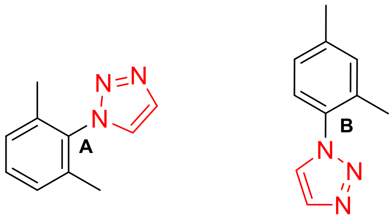

3t

Amination of *m*-xylene with 1*H*-1,2,3-triazole: Reaction was proceeded with 2.0 mL *m*-xylene and 3.0 mL CH_3CN. After 24 h, the reaction mixture was purified by silica gel column chromatography to afford the product **3t** as an inseparable mixture. The ratio of the mixture was 1:9 (C_A:C_B) as determined by ^1H NMR of the isolated product. 27.2 mg pale yellow liquid (yield: 53%, 0.3 mmol scale).

^1H NMR (400 MHz, $CDCl_3$) δ 7.91 (d, *J* = 0.9 Hz, 0.10H), 7.84 (d, *J* = 0.9 Hz, 0.90H), 7.74 (d, *J* = 0.9 Hz, 0.90H), 7.67 (d, *J* = 0.9 Hz, 0.10H), 7.20 (t, *J* = 7.9 Hz, 2H), 7.13 (d, *J* = 8.0 Hz, 1H), 2.40 (s, 2.89H), 2.15 (s, 2.89H), 1.98 (s, 1.22H). ^{13}C NMR (101 MHz, $CDCl_3$) δ 140.02, 135.53, 134.07, 133.75, 133.58, 133.45, 132.08, 130.07, 128.50, 127.45, 125.87, 125.35, 125.24, 21.21, 17.75, 17.37. HRMS (ESI) calcd for $C_{10}H_{12}N_3^+$, $[M+H]^+$, 174.1026, found 174.1019.

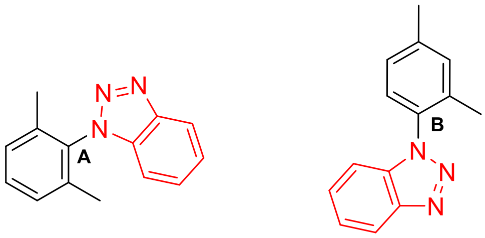

3u

Amination of *m*-xylene with 1*H*-benzo[d][1,2,3]triazole: Reaction was proceeded with 1.2 mmol *m*-xylene and 5.0 mL CH_3CN. After 24 h, the reaction mixture was purified by silica gel column chromatography to afford the product **3u** as an inseparable mixture. The ratio of the mixture was 1:12 (C_A:C_B) as determined by ^1H NMR. 54.3 mg colorless liquid (yield: 81%, 0.3 mmol scale).

^{1}H NMR (400 MHz, DMSO) δ 8.22 – 8.15 (m, 1H), 7.60 – 7.54 (m, 1H), 7.52 – 7.40 (m, 2H), 7.39 – 7.33 (m, 2H), 7.31 (d, *J* = 8.3 Hz, 0.10H), 7.26 (d, *J* = 8.0 Hz, 0.90H), 2.39 (s, 2.77H), 1.96 (s, 2.77H), 1.81 (s, 0.46H). ^{13}C NMR (101 MHz, DMSO) δ 144.87, 144.78, 139.95, 135.89, 134.24, 133.51, 132.23, 132.08, 130.42, 128.78, 128.68, 128.43, 127.78, 126.81, 124.42, 124.36, 119.70, 119.53, 110.40, 109.91, 20.76, 17.10, 16.91. HRMS (ESI) calcd for $C_{14}H_{14}N_3^+$, [M+H]$^+$, 224.1182, found 224.1179.

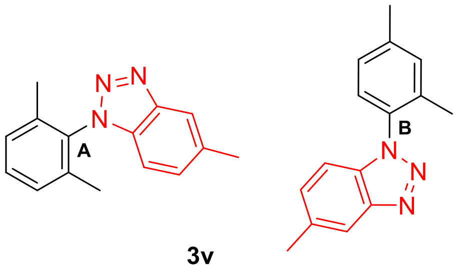

3v

Amination of *m*-xylene with 5-Methyl-1*H*-benzo[d][1,2,3]triazole: Reaction was proceeded with 1.2 mmol *m*-xylene and 5.0 mL CH_3CN. After 24 h, the reaction mixture was purified by silica gel column chromatography to afford the product **3t** as an inseparable mixture. The ratio of the mixture was 1:11 (C_A:C_B) as determined by ^{1}H NMR. 47.0 mg pale yellow liquid (yield: 66%, 0.3 mmol scale).

^{1}H NMR (400 MHz, DMSO) δ 8.03 – 7.78 (m, 1H), 7.36 – 7.07 (m, 5H), 2.41 (s, 1.81H), 2.37 (s, 1.25H), 2.33 (s, 2.75H), 1.89 (s, 2.75H), 1.74 (d, *J* = 2.9 Hz, 0.50H). ^{13}C NMR (101 MHz, DMSO) δ 145.44, 143.48, 139.87, 139.84, 138.79, 135.93, 135.86, 134.32, 134.16, 133.99, 132.35, 132.31, 132.06, 132.03, 130.39, 128.74, 127.76, 126.85, 126.72, 126.47, 118.99, 118.28, 109.95, 109.34, 17.11, 16.94, 16.90. HRMS (ESI) calcd for $C_{15}H_{16}N_3^+$, [M+H]$^+$, 238.1339, found 238.1334.

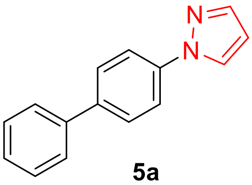

5a

Amination of biphenyl with pyrazole[3]: Reaction was proceeded with 1.2 mmol 1,1'-biphenyl and 5.0 mL CH_3CN. After 24 h, the reaction mixture was purified by silica gel column chromatography to afford product **5a**. 62.8 mg white solid (yield: 95%, 0.3 mmol scale).

^{1}H NMR (400 MHz, $CDCl_3$) δ 7.96 – 7.89 (m, 1H), 7.79 – 7.71 (m, 3H), 7.69 – 7.63 (m, 2H), 7.62 – 7.58 (m, 2H), 7.44 (t, *J* = 7.5 Hz, 2H), 7.38 – 7.32 (m, 1H), 6.47 (t, *J* = 2.0 Hz, 1H). ^{13}C NMR (101 MHz, CDCl3) δ 141.26, 140.15, 139.43, 139.37, 128.97, 128.13, 127.58, 127.04, 126.79, 119.50, 107.81.

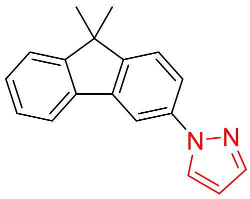

5b

Amination of 9,9-dimethyl-9*H*-fluorene with pyrazole: Reaction was proceeded with 1.2 mmol 9,9-dimethyl-9*H*-fluorene and 5.0 mL CH_3CN. After 24 h, the reaction mixture was purified by silica gel column chromatography to afford the product **5b**. 49.8 mg milky liquid (yield: 64%, 0.3 mmol scale).

^{1}H NMR (400 MHz, $CDCl_3$) δ 8.00 – 7.93 (d, *J* = 2.4 Hz, 1H), 7.82 (d, *J* = 1.9 Hz, 1H), 7.77 – 7.73 (m, 2H), 7.73 – 7.70 (m, 1H), 7.59 (dd, *J* = 8.0, 1.7 Hz, 1H), 7.47 – 7.41 (m, 1H), 7.38 – 7.27 (m, 2H), 6.48 (t, *J* = 1.8 Hz, 1H), 1.52 (s, 6H). ^{13}C NMR (101 MHz, $CDCl_3$) δ 155.26, 153.81, 141.08, 139.56, 138.40, 137.75, 127.44, 127.23, 127.09, 122.76, 120.75, 120.10, 118.17, 114.33, 107.66, 47.27, 27.20. HRMS (ESI) calcd for $C_{18}H_{17}N_2^+$, $[M+H]^+$, 261.1386, found 261.1382.

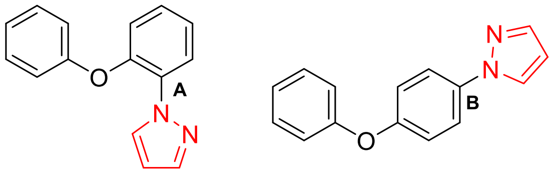

5c

Amination of oxydibenzene with pyrazole[2]: Reaction was proceeded with 1.2 mmol oxydibenzene and 5.0 mL CH_3CN. After 24 h, the reaction mixture was purified by silica gel column chromatography to afford the product **5c** as an inseparable mixture The ratio of the mixture was 1:7.3 (C_A:C_B) as determined by ^{1}H NMR. 45.4 mg pale yellow solid (yield: 64%, 0.3 mmol scale).

^{1}H NMR (400 MHz, DMSO) δ 8.44 (d, *J* = 2.4 Hz, 0.88H), 8.15 (d, *J* = 2.4 Hz, 0.12H), 7.88 – 7.82 (m, 1.78H), 7.80 (dd, *J* = 7.8, 1.8 Hz, 0.15H), 7.73 (d, *J* = 1.6 Hz, 0.86H), 7.70 (d, *J* = 1.6 Hz, 0.14H), 7.44 – 7.37 (m, 2H), 7.18 – 7.08 (m, 3H), 7.07 – 6.95 (m, 2H), 6.52 (dd, *J* = 2.3, 1.9 Hz, 0.87H), 6.44 (dd, *J* = 2.3, 1.9 Hz, 0.13H).^{13}C NMR (101 MHz, DMSO) δ 156.71, 156.26, 154.71, 147.47, 140.82, 140.43, 135.63, 131.32, 130.14, 130.09, 130.06, 128.47, 127.70, 125.47, 124.74, 123.60, 123.44, 120.68, 120.24, 119.59, 118.63, 118.56, 117.86, 107.75, 107.07. HRMS (ESI) calcd for $C_{15}H_{13}N_2O^+$, $[M+H]^+$, 237.1022, found 237.1019.

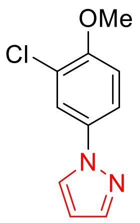

5d

Amination of 1-chloro-2-methoxybenzene with pyrazole[2]: Reaction was proceeded with 1.2 mmol 1-chloro-2-methoxybenzene and 5.0 mL CH_3CN. After 24 h, the reaction mixture was purified by silica gel column chromatography to afford the product **5d**. 25.7 mg pale yellow liquid (yield: 41%, 0.3 mmol scale).

^{1}H NMR (400 MHz, $CDCl_3$) δ 7.83 (d, J = 2.4 Hz, 1H), 7.74 (d, J = 2.7 Hz, 1H), 7.70 (d, J = 1.6 Hz, 1H), 7.54 (dd, J = 8.9, 2.7 Hz, 1H), 6.99 (d, J = 8.9 Hz, 1H), 6.45 (t, J = 2.1 Hz, 1H), 3.94 (s, 3H). ^{13}C NMR (101 MHz, $CDCl_3$) δ 153.74, 141.15, 134.24, 126.90, 123.27, 121.83, 121.02, 118.69, 114.61, 112.45, 107.76, 56.58. HRMS (ESI) calcd for $C_{10}H_{10}ClN_2O^+$, $[M+H]^+$, 209.0476, found 209.0474.

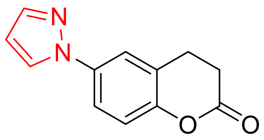

5e

Amination of chroman-2-one with pyrazole[2]: Reaction was proceeded with 1.2 mmol chroman-2-one and 5.0 mL CH_3CN. After 24 h, the reaction mixture was purified by silica gel column chromatography to afford the product **5e**. 28.3 mg pale yellow liquid (yield: 44%, 0.3 mmol scale).

^{1}H NMR (400 MHz, DMSO) δ 8.43 (d, J = 2.0 Hz, 1H), 7.77 (s, 1H), 7.70 (d, J = 8.1 Hz, 2H), 7.15 (dd, J = 8.7, 1.5 Hz, 1H), 6.53 – 6.50 (m, 1H)., 3.04 (t, J = 7.2 Hz, 2H), 2.80 (t, J = 7.2 Hz, 2H). ^{13}C NMR (101 MHz, DMSO) δ 168.17, 149.73, 140.91, 135.97, 127.75, 124.69, 118.36, 117.87, 117.22, 107.86, 28.37, 22.96.

Supplementary References

1. de Oliveira, A. L., Alves de Oliveira, C. H., Mairink, L. M., Pazini, F., Menegatti, R. & Lião, L. M. Complete assignment of NMR data of 22 phenyl-1H-pyrazoles' derivatives. *Magn. Reson. Chem.* **49**, 537–542 (2011).
2. Romero, N. A., Margrey, K. A., Tay, N. E. & Nicewicz, D. A. Site-selective arene C-H amination via photoredox catalysis. *Science* **349**, 1326-1330 (2015).

3. Taillefer, M., Xia, N. & Ouali, A. Efficient iron/copper co-catalyzed arylation of nitrogen nucleophiles. *Angew. Chem. In. Ed.* **46**, 934-936 (2007).
4. Asaumi, T., Matsuo, T., Fukuyama, T., Ie, Y., Kakiuchi, F., & Chatani, N. Ruthenium- and rhodium-catalyzed direct carbonylation of the ortho C−H bond in the benzene ring of N-arylpyrazoles. *J. Org. Chem.* **69**, 4433-4440 (2004).
